# Carbon Metabolism of Enterobacterial Human Pathogens Growing in Epithelial Colorectal Adenocarcinoma (Caco-2) Cells

**DOI:** 10.1371/journal.pone.0010586

**Published:** 2010-05-11

**Authors:** Andreas Götz, Eva Eylert, Wolfgang Eisenreich, Werner Goebel

**Affiliations:** 1 Lehrstuhl für Mikrobiologie, Biozentrum der Universität Würzburg, Würzburg, Germany; 2 Lehrstuhl für Biochemie, Center of Isotopologue Profiling, Technische Universität München, Garching, Germany; 3 Max-von-Pettenkofer Institut, Ludwig-Maximilian-Universität München, München, Germany; Max Planck Institute for Infection Biology, Germany

## Abstract

Analysis of the genome sequences of the major human bacterial pathogens has provided a large amount of information concerning their metabolic potential. However, our knowledge of the actual metabolic pathways and metabolite fluxes occurring in these pathogens under infection conditions is still limited. In this study, we analysed the intracellular carbon metabolism of enteroinvasive *Escherichia coli* (*EIEC* HN280 and *EIEC* 4608-58) and *Salmonella enterica* Serovar Typhimurium (*Stm* 14028) replicating in epithelial colorectal adenocarcinoma cells (Caco-2). To this aim, we supplied [U-^13^C_6_]glucose to Caco-2 cells infected with the bacterial strains or mutants thereof impaired in the uptake of glucose, mannose and/or glucose 6-phosphate. The ^13^C-isotopologue patterns of protein-derived amino acids from the bacteria and the host cells were then determined by mass spectrometry. The data showed that *EIEC* HN280 growing in the cytosol of the host cells, as well as *Stm* 14028 replicating in the *Salmonella*-containing vacuole (SCV) utilised glucose, but not glucose 6-phosphate, other phosphorylated carbohydrates, gluconate or fatty acids as major carbon substrates. *EIEC* 4608-58 used C_3_-compound(s) in addition to glucose as carbon source. The labelling patterns reflected strain-dependent carbon flux via glycolysis and/or the Entner-Doudoroff pathway, the pentose phosphate pathway, the TCA cycle and anapleurotic reactions between PEP and oxaloacetate. Mutants of all three strains impaired in the uptake of glucose switched to C_3_-substrate(s) accompanied by an increased uptake of amino acids (and possibly also other anabolic monomers) from the host cell. Surprisingly, the metabolism of the host cells, as judged by the efficiency of ^13^C-incorporation into host cell amino acids, was not significantly affected by the infection with either of these intracellular pathogens.

## Introduction

Enteroinvasive *Escherichia coli* (*EIEC)*, highly related to *Shigella* species [1], and *Salmonella enterica* Serovar Typhimurium (abbreviated *S*. Typhimurium in the following) are typical intracellular pathogens, causing bloody diarrhoea and gastroenteritis, respectively, in humans [2]. Both microorganisms are mainly food-borne pathogens and hence reach their target cells after crossing the intestinal epithelium. In the infected host cells, *EIEC* similar as *Shigella* escapes from the primary phagosome into the cytosol [3], whereas *S*. Typhimurium remains in a specialized phagosomal compartment [4], termed *Salmonella*-containing vacuole (SCV).

*EIEC/Shigella* and *S*. Typhimurium are typical heterotrophic and prototrophic bacteria. The genome sequences [5], [6] show that these pathogens share a rather similar metabolic setting including all genes for the major central metabolic pathways, i.e. for glycolysis, the Entner-Doudoroff pathway, the pentose phosphate cycle, the citrate (TCA) cycle, the biosyntheses for all amino acids, as well as for the main anapleurotic reactions including the glyoxylate shunt. Accordingly, both pathogens grow *in vitro* under similar conditions. Glucose is a preferred carbon source for growth, taken up by *EIEC/Shigella* and *S*. Typhimurium mainly by the two PEP-dependent phosphotransferase systems (PTS), PtsG/Crr and ManXYZ [7]. Glucose 6-phosphate (glucose-6P) can also be taken up by both pathogens via highly homologous UhpT transporters [8]. In both enterobacteria, the *uhpT* gene is under the control of a complex two-component system (*uhpABC*) [9] and carbon catabolite repression (CCR). Transcription of *uhpT* is therefore very low in the presence of glucose. Both pathogens are able to catabolise various C_2_-, C_3_-, C_4_- and C_5_-substrates and, hence, fatty acids, glycerol, pyruvate, lactate and C_4_-dicarboxylates may also be possible carbon substrates under certain conditions including growth within mammalian host cells.

First information on the intracellular carbon metabolism of *Shigella* and *S*. Typhimurium came from studies using various *in vivo* expression technologies (IVET), differential gene expression profiling (DGEP) and animal infection experiments with mutants defective in specific catabolic or anabolic reactions [10], [11]. These studies show that in cytosolically growing *Shigella flexneri* the genes encoding the glucose transporters (*ptsG/crr* and *manXYZ*) are repressed, while those of *uhpT, glpF* and *ugp* encoding transporters for glucose-6P, glycerol and glycerol 3-phosphate, respectively, are induced. The glycolysis genes are down-regulated, while those for gluconeogenesis (*fbp* and *pps*) are up-regulated. These data favour glycerol and/or glycerol-P as major carbon source for cytosolically replicating *Shigella.* The strong virulence attenuation of a*roB-D*, *gua*BA and *thyA* mutants of *S. flexneri* defective in the biosyntheses of aromatic amino acids, guanine and thymidine [12], [13], [14] further suggests that these anabolic monomers have to be synthesised by intracellular *Shigella*.

In *S*. Typhimurium replicating in J774 macrophages or HeLa cells, genes encoding key enzymes for glycolysis and the Entner-Doudoroff pathway are up-regulated, while most genes for the enzymes of the citrate (TCA) cycle are repressed, favouring C_6_-sugars as main carbon source for intracellular metabolism. Glucose, glucose-6P and gluconate are suggested as possible carbon substrates [10]. A recent study [15] analyzing the growth of *S*. Typhimurium mutants defective in glucose uptake and/or glycolyis in macrophages and in mice confirms the need of glucose and glycolysis for intracellular carbon metabolism. In systemically infected mice where *S*. Typhimurium mainly replicates in phagocytic cells, intracellular growth depends on a functional TCA cycle [16]. Specifically, mutants blocked in either one of the three successive enzymatic reactions converting succinate to oxaloacetate are avirulent [17], while mutants blocked in gluconeogenesis are fully virulent [16]. From these data, it is concluded that *S*. Typhimurium grows in mouse phagocytes on a limited supply of glycolytic carbon sources, presumably glucose and/or other C_6_-carbohydrates [17]. Upon systemic infections in mice, strong attenuation is observed for *S*. Typhimurium mutants defective in the production of purines (especially of adenine) and aromatic amino acids including histidine, while moderate attenuation is found for pyrimidine and methionine mutants [18], [19], [20]. This supports that these anabolic compounds have to be synthesised in intracellularly replicating *S*. Typhimurium.

Generally, the quantitative analysis of metabolic pathways and fluxes is crucial to understand the life style (e.g. nutrient usage) of pathogenic microorganisms under *in vivo* conditions, as well as their metabolic crosstalk with the respective host cells. One of the most important methods for identifying and quantifying reactions in central metabolism is steady-state metabolic flux analysis (MFA) using ^13^C-labelled precursors (e.g. glucose) [21], [22], [23], [24]. In this approach, the labelling patterns of stable products (e.g. amino acids) at isotopic and metabolic steady-state are determined by NMR and/or gas chromatography coupled to mass spectrometry (GC-MS). The labelling data are then used as constraints in calculations of flux rates on the basis of model metabolic networks. MFA is well established as a tool to analyse carbon metabolism and metabolite fluxes in bacteria including *E. coli* typically growing under chemostat conditions in minimal medium with known uptake, consumption and utilisation of substrates [25], [26], [27], [28], [29]. Since these controlled conditions can hardly be realized in host/pathogen interactions with undefined multiple nutrient usage, the same methodology can not be used for determining flux rates in organismic communities. Nevertheless, ^13^C-labelling of infected host cells followed by a model-free analysis of the ^13^C-label distribution in metabolites from intracellular bacteria and their respective host cells can provide substantial information about the nutrient usage and metabolic reactions occurring during infection. We have termed this observation-driven procedure “^13^C-isotopologue profiling”. The applicability and power, but also the limitations of ^13^C-isotopologue profiling have been shown recently in a study of the carbon metabolism of *Listeria monocytogenes* replicating within macrophage cells [30].

In this report, we use ^13^C-isotopologue profiling to study the intracellular carbon metabolism of enteroinvasive *E. coli* and *S*. Typhimurium wild-type strains and mutants defective in the uptake of glucose and/or glucose-6P. The results indicate strain-specific differences in the intracellular metabolism of these human pathogens and allow conclusions about the major carbon sources and metabolic pathways used by these pathogens when replicating in their specific host cell compartments.

## Results

### The analysed enterobacterial wild-type strains and mutants impaired in uptake of glucose, mannose and/or glucose 6-phosphate

For the study on the intracellular metabolism of enteroinvasive *Escherichia coli* (*EIEC*) and *Salmonella enterica* Serovar Typhimurium, we used two *EIEC* isolates, *EIEC* HN280 (in the following termed *EIEC*-1) and *EIEC* 4608-58 (subsequently termed *EIEC*-2) belonging to different groups [31], and the *S*. Typhimurium strain 14028 (abbreviated *Stm*). Mutants of all three strains impaired in the uptake of glucose (deletions in *ptsG* and *manXYZ* - in the following termed glucose-uptake mutant) and/or glucose-6P (deletion in *uhpT* - in the following termed glucose-6P uptake mutant) were recently constructed [32]. *In vitro* and *in vivo* growth of all strains was performed in RPMI medium. This medium which contains all amino acids except alanine in concentrations between 0.1 and 2 mM (Table S1) was supplemented with 10 mM glucose, mannose or glucose-6P. Replication within Caco-2 cells of the wild-type and mutant strains were determined as described recently [32]. The data relevant for the present study are summarized in the supplementary Table S2 (see also [32]). The results indicated that *in vitro* growth of the corresponding mutants of all three strains was strongly impaired in the presence of glucose and glucose-6P, respectively but all mutants were still able to grow in Caco-2 cells albeit with strain-specific reduced efficiencies compared to the wild-type strains (see Table S2 and [32]). To understand the intracellular carbon metabolism of these strains we applied the ^13^C-isotopologue profiling technique which we had already successfully used for studying the intracellular carbon metabolism of *Listeria monocytogenes* in J774 macrophages [30].

### Growth properties of the enterobacterial wild-type and mutant strains in Caco-2 cells

Caco-2 cells were used as host cells and cultured in RPMI medium supplemented with 10% foetal calf serum (FCS) and 10 mM glucose which is close to the average value of the intestinal glucose concentration [33], [34]. When these cells reached 80 to 90% confluency they were infected with the two *EIEC* and the *Stm* wild-type strains as well as with the various mutants. At a multiplicity of infection (MOI) of 20 bacteria per host cell about 2×10^6^ *EIEC*-1 wild-type bacteria were taken up by the 2×10^7^ Caco-2 cells used for each infection assay (internalization efficiency, 0.1; Table 1). Even at a MOI of 100 bacteria per host cell, the *EIEC-*2 wild-type strain was internalized by Caco-2 cells about 5 times less efficiently than *EIEC-*1, but grew inside the host cells with a similar rate as *EIEC*-1, i.e. after the 6.5 h infection period (time of harvest), there were 2×10^8^ intracellular *EIEC*-1 bacteria but only 3×10^7^ *EIEC*-2 bacteria (Table 1). *Stm* was taken up with similar efficiency as *EIEC*-1, but its intracellular doubling time was at least three fold longer than that of the *EIEC* isolates (Table 1 and Table S2). However, using a MOI of 100 bacteria per host cell and 8.5 h infection period, there were also about 1×10^8^ intracellular *Stm* bacteria at the time of harvest (Table 1).

**Table 1:** Infection conditions used in this study.

		WT	Δ*uhpT*	Δ*ptsG*, *manXYZ*	Δ*ptsG*, *manXYZ*, *uhpT*
*EIEC*-1	MOI	20	20	75	75
	cfu 1 h p.i.	1.9×10^6^			9.7×10^5^
	cfu 6.5 h p.i.	2.3×10^8^	1.7×10^8^	2.0×10^7^	3.0×10^7^
*EIEC*-2	MOI	100			100
	cfu 1 h p.i.	5.6×10^5^			5.6×10^5^
	cfu 6.5 h p.i.	3.4×10^7^			1.3×10^6^ †
*Stm*	MOI	100	100	100	100
	cfu 1 h p.i.	3.1×10^7^			2.3×10^7^
	cfu 8.5 h p.i.	1.1×10^8^	7.3×10^7^	4.0×10^7^	8.7×10^7^

The multiplicities of infection (MOI) used for this study to infect 2×10^7^ cells per 175 cm^2^ cell culture flask are shown in Table 1. Ten flasks were infected for each strain and experiment. The number of intracellular bacteria per flask at the beginning of the intracellular replication phase 1 h post infection (p.i.) and the number of bacteria at harvest time (6.5 or 8.5 h p.i.) corresponds approximately with the intracellular generation times shown in Table S2. More details on the infection process and the determination of replication rates are given in [32].

†indicates beginning autolysis.

The replication rates of the glucose-6P uptake mutants of all three pathogens were comparable to those of the corresponding wild-type strains. The glucose/glucose-6P uptake mutants of *EIEC*-1 and *EIEC*-2 replicated with about half of the rate of the corresponding wild-type strains. The replication rate of the glucose/glucose-6P uptake mutant of *Stm* was less reduced (about 20%) as compared to the *Stm* wild-type strain [32].

### Glucose consumption by the host cells and the bacterial strains during infection

**Figure 1:**
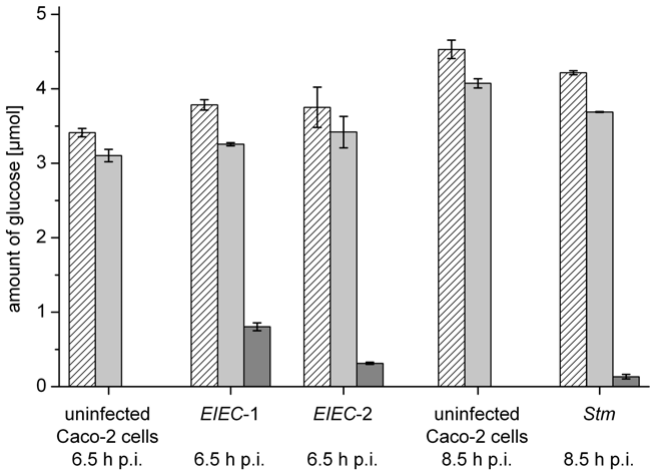
Glucose consumption by the host cells and the intracellular bacteria. Infection of Caco-2 cells with the three enterobacterial wild-type strains was carried out as described in Materials and Methods for 6 and 8 h, respectively, using 200 µmol of ^14^C-labelled glucose per 20 ml culture medium. The columns show the amount of ^14^C-label (corresponding to the consumed ^14^C-glucose in µmol) incorporated into the total host cells with or without bacterial infection (hatched bars), into the separated host cells (light gray bars) and into the isolated bacteria (dark grey bars).

For a concise interpretation of the ^13^C-labelling data (described below), we determined the consumption of uniformly ^14^C-labelled glucose by the three enterobacterial wild-type strains after their intracellular replication for 6.5 h (*EIEC*-1 and *EIEC*-2) or 8.5 h (*Stm*) in Caco-2 host cells (applying similar infection conditions as described above using again an external glucose concentration of 10 mM). The results shown in Fig. 1 indicate: (i) The 2×10^7^ Caco-2 cells (in a 175 cm^2^ culture flask) incorporated about 4 µmol, i.e. about 2% of the 200 µmol external ^14^C-glucose (specific activity of 0.5 MBq/mmol) during the infection period. This suggests that we are working in our infection assays with excess of external glucose throughout the infection period. (ii) The amount of ^14^C-label incorporated into the Caco-2 cells was similar with or without infection by the three enterobacterial pathogens, suggesting that these infections do not alter glucose uptake by the Caco-2 cells. (iii) After the 6.5 h infection period, the intracellular 2×10^8^ *EIEC*-1and 10^7^ *EIEC*-2 bacteria incorporated ^14^C-label corresponding to 0.8 µmol and 0.3 µmol glucose, respectively. The ^14^C-label incorporated into the 10^8^ intracellular *Stm* during the 8.5 h infection period corresponds to 0.1 µmol glucose. (iv) Related to the numbers of intracellular bacteria present in the Caco-2 host cells after these infection times the data suggest that the two *EIEC* strains incorporate ^14^C-label which corresponds to a roughly equal amount of glucose per bacterial cell while *Stm* incorporated 5–8 times less per bacterial cell. These calculated glucose consumption data thus concur with the observed intracellular replication rates of the three pathogens (Table S2).

### ^13^C-Isotopologue profiling procedure

The method applied in the following for the study of carbon metabolism of the three pathogens uses as major ^13^C-labelled carbon substrate uniformly labelled ^13^C-glucose ([U-^13^C_6_]glucose). This carbon substrate is degraded in the central catabolic pathways leading to specific distributions of the ^13^C-label in the arising catabolic intermediates and subsequently in many anabolic products originating from these intermediates (e.g. amino acids which we used in our study). Although the biosyntheses of amino acids follow highly conserved pathways, the catabolic intermediates acting as precursors for these biosyntheses may be produced by different reactions (e.g. pyruvate can be generated by glycolysis, the Entner-Doudoroff, the pentose-phosphate cycle from carbohydrates or by catabolism of certain amino acids and other carbon sources; likewise acetyl-CoA can be generated by oxidation of lactate, pyruvate, fatty acids etc.). As a consequence, specific ^13^C-isotopologue distributions (^13^C-isotopologue patterns or profiles) are generated in amino acids reflecting the metabolic history of their precursors. The ^13^C**-**profiles of the amino acids (and other possible stable metabolites that may be used for ^13^C**-**profiling) were routinely determined in our study by quantitative mass spectrometry (MS). Although more direct information regarding the ^13^C-distribution within ^13^C-labelled metabolites can be obtained by NMR, MS showing a higher sensitivity - as little as 10^7^ bacteria may be sufficient for MS-based ^13^C-isotopologue profiling [30] - was applied here, since we had to deal with rather low numbers of intracellular bacteria that could be isolated from the infected host cells. Although ^13^C-isotopologue profiling is in principle applicable to any labelled metabolite detectable by GC/MS or LC/MS, this method is particularly well established for amino acids [25], [26], [27], [28], [29]. After separation of host cells and bacteria, total protein isolated from each partner is hydrolysed under acidic conditions yielding free amino acids. Due to the acidic conditions, Asn and Gln are converted into Asp and Glu, respectively. The values reported below for Asp and Glu are therefore always average values for Asn/Asp respectively Gln/Glu. Moreover, Trp and Cys are destroyed by acidic hydrolysis. After silylation of the amino acids and separation of these derivatives by GC, the ^13^C-isotopologue composition is analysed by MS showing the abundance of molecular species exceeding the mass of the parent ion (M) by one or more mass units (M+1, M+2, M+3, etc.). Arg as well as Met can hardly be detected in typical GC/MS runs due to low signal to noise ratios (see also [21] for discussion of the method). The analysis of the ^13^C-isotopologue profiles in the ^13^C-labelled and hence *de novo* synthesised amino acids from the bacteria and host cells allows the determination of the ^13^C-incorporation rate (excess ^13^C) of the catabolic intermediate(s) derived from the supplied [U-^13^C_6_]glucose leading to the formation of a given ^13^C-labelled amino acid. From these data, the contribution of reactions and pathways leading to the formation of the precursors for a specific amino acid were deduced.

### Incorporation of ^13^C-label from external [U-^13^C_6_]glucose into amino acids of the intracellularly growing *EIEC* and *Stm* wild-type strains

For the determination of the intracellular carbon metabolism, we used a similar ^13^C-isotopologue profiling protocol as reported previously for *L. monocytogenes* [30]. In short, Caco-2 cells were again infected with *EIEC*-1, *EIEC*-2 or *Stm* with MOI of 20 and 100 bacteria, respectively per Caco-2 cell as described above (see also Table 1). The infected cells were incubated in RPMI medium (containing 10 mM [U-^13^C_6_]glucose) for 6 h (in case of the two *EIEC* strains) or 8 h (in case of *Stm*). At the time of harvest, there were again approximately 10^8^ *EIEC*-1 and *Stm* bacteria and 10^7^ *EIEC*-2 bacteria in 2×10^7^ host cells applied in each infection assay. Bacteria were separated from the host cells by differential centrifugation.

**Figure 2:**
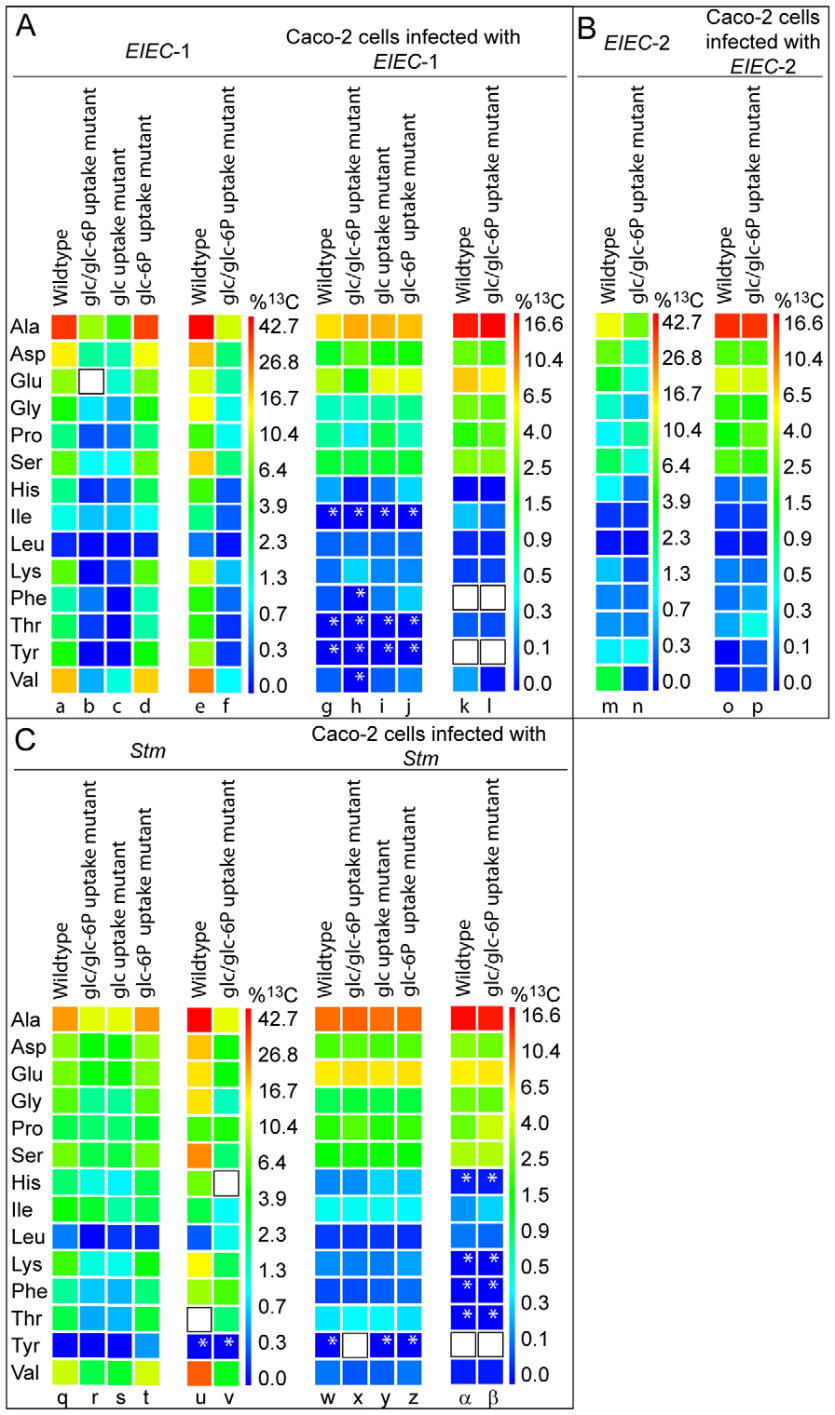
^13^C-Incorporation into amino acids of the intracellular bacteria and the infected Caco-2 host cells (see also Table S3). The colours (according to a quasi logarithmic scale) indicate the amount of ^13^C-incorporation in each amino acid. (A) *EIEC*-1 wild-type strain, the indicated mutants of this strain (lanes a-f), and the corresponding Caco-2 cells (g-l). Colours in (a-d)/(g-j) and (e,f)/(k,l) show ^13^C-incorporation into amino acids of slow and faster proliferating Caco-2 cells, respectively. (B) *EIEC*-2, isogenic glucose/glucose-6P uptake mutant (m-n), and the Caco-2 cells infected with these two strains (o-p). (C) *Stm* and the indicated mutants; (q-β) show the ^13^C-incorporation into amino acids of the bacterial strains and the infected Caco-2 cells in the same order as in (A). The small white stars mark values with high standard deviations (see also Tables S3 and S4). White boxes indicate amino acids that could not be detected.

In the *EIEC*-1 *strain*, all amino acids detectable by this method, except Leu, acquired significant ^13^C-label from exogenous [U-^13^C_6_]glucose. The ^13^C-incorporation rates varied, however, considerably in the individual amino acids ranging from 1% excess of ^13^C-label (in Ile) to 33% (in Ala). These data are shown by a colour-code in Fig. 2A (lane a) and as numerical values in Table S3.

In the *EIEC*-2 strain only six amino acids (Ala, Asp, Glu,Val, Ser and Gly) were ^13^C-labelled with incorporation rates ranging from 1% excess ^13^C-label (in Gly) to 12% (in Ala) as shown in Fig. 2B, lane m and Table S3.Notably, amino acids whose biosynthesis depends on intermediates of the pentose phosphate cycle (i.e. Tyr, Phe and His - requiring erythrose 4-phosphate and phosphoribosyl pyrophosphate, respectively), but also Lys and Thr, remained practically unlabelled. Thus, the *de novo* synthesis of amino acids (as typical anabolic monomers) appears to be considerably higher in *EIEC*-1 than in *EIEC*-2 when replicating in Caco-2 cells.

*Stm* growing in the SCV of Caco-2 cells exhibits still another pattern of ^13^C-labelled amino acids. More specifically, 10 amino acids acquired excess ^13^C-label from [U-^13^C_6_]glucose with ^13^C excess rates ranging from 2% (in Phe and His) to 21% (in Ala) (Fig. 2C, lane q, Table S3). The rather high ^13^C-enrichment values of the amino acids in the SCV-residing *Stm* indicate that active *de novo* synthesis of amino acids occurs in the intracellular *Stm* bacteria. Hence, transport of [U-^13^C_6_]glucose or derivative(s) of it generated by the host cell into the SCV must be quite efficient.

The nature of the ^13^C-labelled carbon source(s) provided by the host cells to these intracellular bacteria is analysed in the following section using the glucose and/or glucose-6P uptake mutants.

### ^13^C-Incorporation into amino acids of *EIEC* and *Stm* glucose and/or glucose-6P uptake mutants

To determine whether the ^13^C*-*label in amino acids of the three enterobacterial strains originates directly from [U-^13^C_6_]glucose or catabolic intermediates produced from [U-^13^C_6_]glucose (and hence also ^13^C-labelled) by the host and subsequently transferred to the bacteria, we performed labelling experiments with mutants that are impaired in the transport of glucose, glucose-6P or both, following the same experimental settings used before for the corresponding parental strains. As described above, all glucose- and glucose/glucose-6P- uptake mutants are still able to replicate, albeit at reduced rates, in Caco-2 cells [32]. This already suggests that the mutants can use - in the absence of glucose - (an) alternative carbon substrate(s).

The ^13^C-enrichment in the amino acids from the *EIEC*-1 glucose/glucose-6P uptake mutant was strongly reduced compared to that in the corresponding amino acids from the parental strain (reduction by 50% in Glu to 95% in Thr). Notably, only three amino acids acquired excess ^13^C-label above 1% (Ala>Glu>Asp) (Fig. 2A, lane b and Table S3). This result confirms that a large portion (> 50%) of *de novo* synthesised amino acids directly derived from [U-^13^C_6_]glucose (or [U-^13^C_6_]glucose-6P) in the parental strain.

To distinguish whether glucose and/or glucose-6P function as major carbon nutrients for intracellular *EIEC*-1, the glucose-6P- and the glucose-uptake mutants of this *EIEC* strain were compared. Whereas the ^13^C-incorporation rates into amino acids were similar for the parent strain and the glucose-6P uptake mutant (Fig. 2A, lanes a and d), the values for amino acids from the glucose uptake mutant were reduced to a similar extent as in the glucose/glucose-6P uptake mutant (Fig. 2A, lanes b and c). This provides clear evidence that glucose, but not glucose-6P, is the preferential carbon source for the intracellular metabolism of *EIEC*-1 under the applied conditions.

In contrast to the glucose/glucose-6P uptake mutant of *EIEC*-1 the corresponding mutant of *EIEC*-2 exhibited less reduction in ^13^C-incorporation into the *de novo* synthesised amino acids (Fig. 2B, lanes m and n; Table S3) suggesting that glucose (or glucose-6P) is less efficiently utilised in this strain as carbon source for the intracellular metabolism than in *EIEC*-1 and alternative carbon source(s) may be consumed in addition to glucose. As indicated by the more detailed analyses of the isotopologue distribution in amino acids (see below), C_3_-compounds are potential nutrients under these conditions.

The glucose/glucose-6P uptake mutant of *Stm* showed a strongly reduced ^13^C-incorporation (by >50%) into the (fewer) *de novo* synthesised amino acids as compared to the wild-type strain (Fig. 2C, lanes q and r; Table S3). The glucose-6P uptake mutant incorporated ^13^C-label into all amino acids as efficiently as the wild-type strain, while the glucose uptake mutant incorporated ^13^C-label with the same low efficiency as the glucose/glucose-6P uptake mutant (Fig. 2C, lanes s and t). This shows that glucose, but not glucose-6P, is a major carbon source for *Stm* when replicating in the SCV of Caco-2 cells.

### Faster proliferating Caco-2 cells show higher^ 13^C-incorporation into amino acids of the host cells and the bacterial wild-type strains but not of the glucose/glucose-6P uptake mutants

In faster proliferating Caco-2 cells (obtained by using a less dense monolayer of Caco-2 cells, i.e. about 60% confluency instead of about 90% used in the previous experiments), the ^13^C-incorporation into the *de novo* synthesised amino acids of the Caco-2 cells and the intracellularly replicating *EIEC*-1 and *Stm* wild-type strains (*EIEC*-2 was not tested) was significantly enhanced. However, this increased ^13^C-incorporation was not observed in the glucose/glucose-6P uptake mutants of these strains (Fig. 2A and C, lanes e, f and u, v). The reason for the higher ^13^C-incorporation is apparently an enhanced glucose uptake by these Caco-2 cells, also resulting in enhanced [U-^13^C_6_]glucose uptake by the bacteria. Subsequently, this leads to higher ^13^C-incorporation into amino acids of the host cells (Fig. 2A, lanes k compared to l and α compared to β) and the wild-type bacterial strains (compare Fig. 2A, lanes a compared to e and q compared to u), but not of the glucose/glucose-6P uptake mutants (compare Fig. 2A, lanes b compared to f and r compared to v). This observation further supports the assumption that glucose is the preferential carbon source for the intracellularly replicating *EIEC*-1 and *Stm* wild-type strains, but not of the glucose/glucose-6P uptake mutants.

### Comparison of the^ 13^C-isotopologue patterns of the amino acids in the wild-type strains and the glucose/glucose-6P uptake mutants of the three pathogens

The comparison of the ^13^C-incorporation rates between the wild-type strains and the glucose/glucose-6P uptake mutants already allows conclusions about the nature of the preferred carbon sources as described in the previous section. However, more specific insights into the intracellular metabolism can be obtained from the isotopologue distributions in each of the ^13^C-labelled amino acids.

To better follow our conclusions, the reader is also referred to Figs. S1 and S2 showing the major catabolic pathways and anapleurotic reactions operating in all three pathogens and the expected as well as the observed ^13^C-isotopologues when [U-^13^C_6_]glucose or its derivatives are channelled through these pathways.

### Amino acids derived from glycolytic intermediates (Ala, Val, Ser, Gly)

#### (a) in the wild-type strains

**Figure 3:**
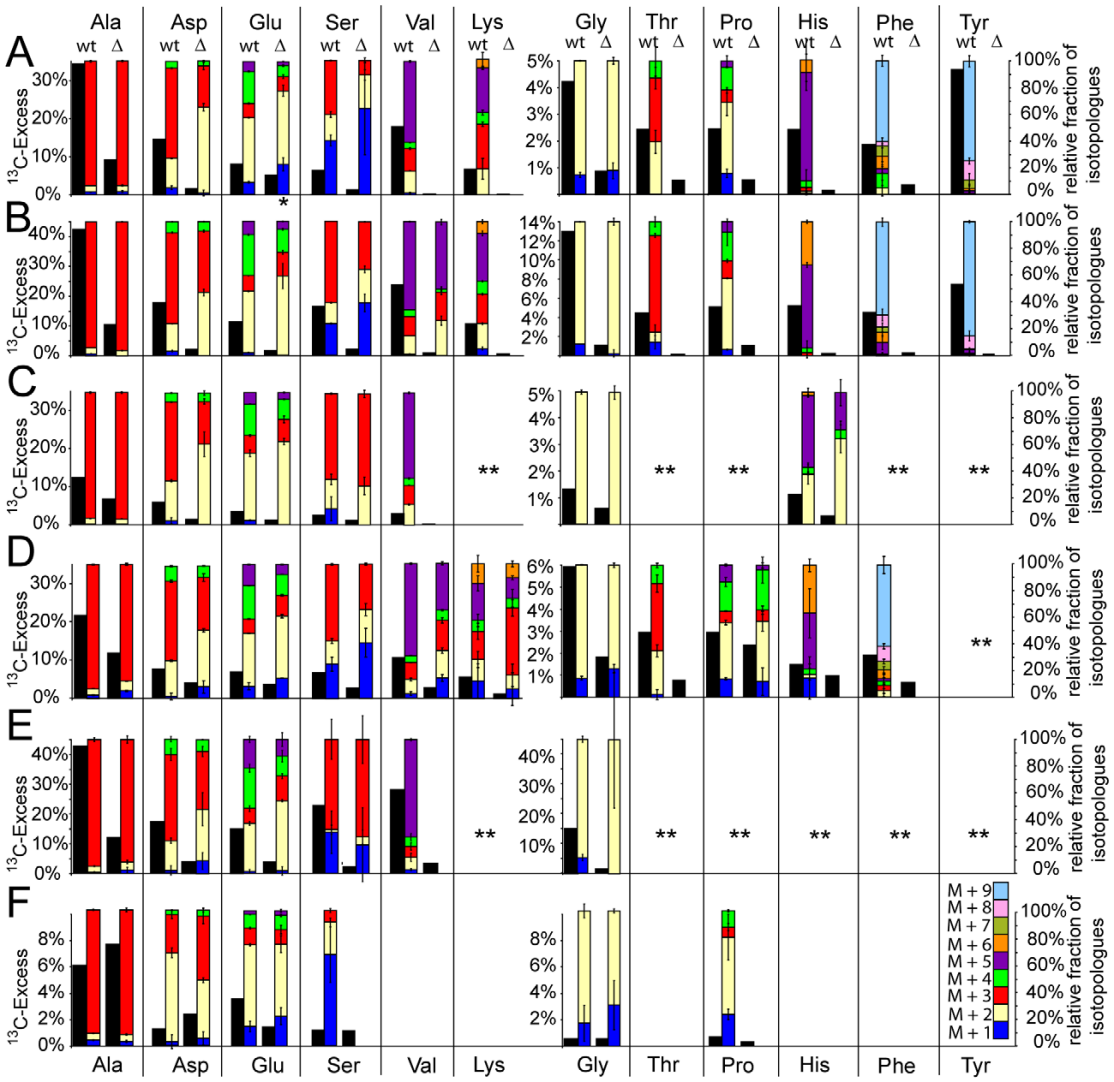
^13^C-Isotopologue distribution in the amino acids *de novo* synthesised in intracellular *EIEC*-1 (A), *EIEC*-2 (C) and *Stm* (D) replicating in slower proliferating Caco-2 cells as well as *EIEC*-1 (B) and *Stm* (E) replicating in faster proliferating Caco-2 cells (see text for details); Caco-2 cells infected with *EIEC*-1 (F) (coloured bars, see also Table S4). The ^13^C-distribution of the glucose-6P uptake mutants is similar to wt and that of the glucose uptake mutant similar to the glucose/glucose-6P uptake mutants (Δ) and therefore not shown (but see Table S4).The coloured boxes indicate the percentage of the ^13^C-isotopologues (M+1 to M+9) of the different amino acids (right scales). The black bars indicate the overall amount of ^13^C-incorporation (in mol%) into amino acids of the wild-type strains (wt) and their corresponding glucose/glucose-6P uptake mutants (Δ) as well as in the Caco-2 cells, infected with wt and Δ strains (left scales). Note that two left scales were used in A-F to better differentiate the ^13^C-incorporation into the various *de novo* synthesised amino acids of the three intracellular bacteria and their mutants. * indicates that data from glucose uptake mutant are displayed. ** These data are not shown in the figure due to high standard deviations or missing data points (see Table S4).

Ala, although *de novo* synthesised to a different extent in *EIEC*-1, *EIEC*-2 and *Stm* (Fig. 2 and Fig. 3A-E, black bars, scale at the left side), is characterised by the triple ^13^C-labelled isotopologue at high abundance in all three strains. The relative fractions of this isotopologue (M+3) are indicated by red bars in Fig. 3A-E; scale at right; for numerical values, see Table S4). The metabolic precursor for this ^13^C_3_-Ala isotopologue is ^13^C_3_-pyruvate which may derive in the bacteria either directly from [U-^13^C_6_]glucose by glycolysis, the Entner-Doudoroff pathway, the pentose phosphate cycle (via ^13^C_3_-glyceraldehyde phosphate) or a combination of these pathways. Alternatively, it can derive from ^13^C_3_-substrates (e.g. glycerol, glycerol 3-phosphate, lactate, or pyruvate) generated in the host cells from [U-^13^C_6_]glucose and subsequently imported (as ^13^C_3_-substrate) into the bacteria and then metabolised to ^13^C_3_-pyruvate/Ala. However, a major participation of host cell-derived ^13^C-labelled carbon substrates which predominantly would lead to ^13^C_2_-labelled acetyl-CoA (e.g. from host cell derived fatty acids, several amino acids and to some extent also lactate and pyruvate) can be excluded as source for bacterial pyruvate, since in this case ^13^C_2_- and ^13^C_1_-isotopologues of pyruvate (and hence Ala) should have mainly been generated via the glyoxylate shunt and PEP carboxykinase. The minor amounts of ^13^C_1_- and ^13^C_2_-Ala observed in all three strains (about 5–8% of the labelled Ala) may indeed be produced by these latter reactions (see below). A major contribution of ^13^CO_2_ (generated by decarboxylation of [U-^13^C_6_]glucose or its intermediates (e.g. pyruvate)) in the formation of ^13^C_1_-labelled components can be excluded since no ^13^C_1_-labelled Asp was detected which should derive from of ^13^C_1_-oxaloacetate generated by PEP carboxylation with ^13^CO_2_.

The ^13^C-isotopologue pattern of Val (produced by two pyruvate molecules with removal of one carbon atom by decarboxylation) mainly showed the ^13^C_5_-isotopologue (purple coloured bar in Fig. 3) which can be explained by the assembly of two ^13^C_3_-labelled pyruvate molecules. There was also a substantial amount of ^13^C_2_- and ^13^C_3_-Val which probably derives from one unlabelled and one ^13^C_3_-labelled pyruvate molecule. The other two branched chain amino acids, Ile and Leu, were not (or in case of *EIEC*-1 only weakly) ^13^C-labelled, suggesting that these amino acids are either not synthesised *de novo* but rather imported from the host cells.

The biosyntheses of Ser and Gly are closely linked. Ser is mainly generated from 3-phosphoglycerate (Fig. S1), while Gly derives from Ser by removal of one C-atom by Ser-hydroxymethyltransferase with the participation of tetrahydrofolate which is thereby converted to *N^5^*, *N^10^*-methylenetetrahydrofolate. However, the generation of Ser from Gly by this reaction is less likely in these pathogens under the applied conditions. In all strains, ^13^C_3_-Ser was the major isotopologue (red bar in Fig. 3), but ^13^C_1_- and ^13^C_2_-Ser (blue and yellow bars, respectively) were also observed in substantial (but different) amounts (Fig. 3A-E). As mentioned before, ^13^C_3_-Ser is apparently produced directly from ^13^C_3_-labelled 3-phosphoglycerate, the latter being generated from [U-^13^C_6_]glucose via the glycolytic pathway (Fig. S1). In contrast, ^13^C_1_- and ^13^C_2_-Ser isotopologues can not be produced by this route but must originate from the corresponding ^13^C-labelled PEP isotopologues which may be produced from ^13^C_2_-oxaloacetate by PEP carboxykinase (see Fig. S2). Since PEP-carboxykinase is induced only at low glucose concentration [35], [36], these data suggest that the amount of [U-^13^C_6_]glucose in the Caco-2 cells which becomes available to the intracellular bacteria must be quite low which is also evident from the data shown in Fig. 1. The observed ^13^C-isotopologues of Gly (mainly ^13^C_2_- and less ^13^C_1_-Gly; Fig. 3A-E) can be explained by their synthesis from the respective ^13^C-Ser isotopologues catalysed by Ser-hydroxymethyltransferase.

### Amino acids derived from glycolytic intermediates (Ala, Val, Ser, Gly)

#### (b) glucose/glucose-6P uptake mutants

In the *EIEC*-1 mutant, ^13^C_3_-Ala remained the predominant ^13^C-isotopologue similar as in the wild-type strain (Fig. 3A and B) suggesting that this mutant – in the absence of ^13^C-glucose – must have access to ^13^C-labelled substrate(s) which again mainly yield(s) ^13^C_3_-pyruvate. The ^13^C_3_-isotopologue of Ser in this mutant was much more reduced than the ^13^C_1_- and ^13^C_2_-isotopologues indicating that in the absence of glucose the residual *de novo* synthesis of Ser now originates more from ^13^C_1_- and ^13^C_2_-labelled 3-phosphoglycerate generated from ^13^C_1_- and ^13^C_2_-PEP (produced from oxaloacetate by PEP carboxykinase) (Fig. S2). The Gly isotopologues can again be explained by their generation from the Ser-isotopologues by elimination of the C_1_-unit. *De novo* synthesis of Val does not seem to occur in this mutant as no ^13^C-labelled Val was observed.

In the *EIEC*-2 mutant, ^13^C_3_-incorporation into Ala, Ser and Gly was less reduced (only by about 50% compared to the wild-type strain) than in the *EIEC*-1 mutant (black bars in Fig. 3C). *De novo* synthesis of Val, already low in the wild-type strain, reached background level in the mutant. Interestingly, the ^13^C-isotopologue patterns of Ala, but also of Ser and Gly, were similar to those of the wild-type strain (Fig. 3C), suggesting that 3-phosphoglycerate serving as precursor of the latter amino acids was generated in the mutant similarly as in the wild-type strain even in the absence of glucose. This confirms that this strain may use, in addition to glucose, a C_3_-substrate, such as glycerol.

In the *Stm* mutant, there were again remarkable differences between the ^13^C-isotopologues patterns observed with the mutant and the wild-type strain, respectively (Fig. 3D and E and Table S4). In Ala, the level of the major ^13^C_3_-isotopologue decreased by about 50% in the mutant, but the levels of the minor ^13^C_1_- and ^13^C_2_-isotopologues remained unchanged or were even higher in the mutant. Likewise, the ^13^C_3_-isotopologue of Ser was stronger reduced (>3 fold) than the ^13^C_1_- and ^13^C_2_-isotopologues (<1.5 fold), again suggesting its generation from ^13^C_1_- and ^13^C_2_-labelled 3-phosphoglycerate, as already discussed above for *EIEC*-1. Interestingly, ^13^C-labelled Val was observed in the *Stm* mutant, but the ^13^C_5_-isotopologue of Val, predominant in the wild-type strain was again more reduced (>5 fold) than the ^13^C_1_-, ^13^C_2_-, ^13^C_3_-, and ^13^C_4_-isotopologues (about 1.5 fold) again suggesting the involvement of ^13^C_1_- and ^13^C_2_-labelled pyruvate (and hence the generation of the corresponding PEP isotopologues by gluconeogenesis) for their production (together with unlabelled pyruvate).

### Amino acids derived from intermediates of the TCA cycle (Glu, Asp, Pro, Thr, Lys)

#### (a) in the wild-type strains

Although the ^13^C-incorporation into Glu (and hence the amount of *de novo* synthesised Glu) considerably differed among the three enterobacterial wild-type strains (Fig. 3A-E, black bars), ^13^C_2_-Glu (yellow bars) was the predominant isotopologue in all three strains followed by ^13^C_4_-Glu (green bars). ^13^C_2_-labelled α-ketoglutarate as precursor of ^13^C_2_-Glu is apparently generated in the TCA cycle from ^13^C_2_-acetyl-CoA and unlabelled oxaloacetate. ^13^C_4_-α-ketoglutarate as precursor of the ^13^C_4_-Glu isotopologue may be produced by a second round of the TCA cycle from ^13^C_2_-oxaloacetate and ^13^C_2_-acetyl-CoA. However, in this case an equal amount of ^13^C_3_-labelled α-ketoglutarate and hence ^13^C_3_-Glu should also have been generated which apparently is not the case. Therefore, a major portion of ^13^C_4_-α-ketoglutarate as precursor of ^13^C_4_-Glu must have been generated in the TCA cycle from a ^13^C_3_-oxaloacetate isotopologue and ^13^C_2_-acetyl-CoA (Fig. S1). Indeed, the major ^13^C-Asp isotopologue was found to be ^13^C_3_-Asp (red bars in Fig. 3A-E; Asp directly derives from oxaloacetate) in all three enterobacteria, followed by the ^13^C_2_-Asp isotopologue (yellow bars). The latter Asp isotopologue most likely derives from ^13^C_2_-oxaloacetate generated again in the TCA cycle from ^13^C_2_-acetyl-CoA and unlabelled oxaloacetate. ^13^C_3_-Oxaloacetate as precursor for the ^13^C_3_-Asp isotopologue could in principle be generated by a second round of the TCA cycle from ^13^C_2_-oxaloacetate and ^13^C_2_-acetyl-CoA. However, the higher amount of ^13^C_3_-Asp compared to ^13^C_2_-Asp (which should be produced in equal amounts in the TCA cycle) favours the predominant formation of ^13^C_3_-Asp from ^13^C_3_-oxaloacetate generated via carboxylation of ^13^C_3_-labelled PEP by PEP carboxylase (Fig. S1; there is no pyruvate carboxylase in *E. coli* and *S.* Typhimurium). The apparent formation of ^13^C_3_-labelled PEP can be explained by conversion of [U-^13^C_6_]glucose by glycolysis (and/or the Entner-Doudoroff pathway via ^13^C_3_-glyceraldehyde phosphate). However, also ^13^C_3_-labelled glycerol or glycerol-3P could serve as precursors, but not carbon substrates mainly yielding ^13^C_2_-acetyl-CoA, like ^13^C-labelled fatty acids. ^13^C_3_-Labelled pyruvate and lactate as major carbon substrates are also less likely for the same reason (see also discussion above for Ala).

Pro was ^13^C-labelled in appreciable amounts only in *EIEC*-1 and *Stm* (Fig. 3A-E, black bars). As expected, the ^13^C-isotopologue patterns of Pro were similar to those in Glu which is the direct precursor of Pro (coloured bars in Fig. 3A-E).

Asp is the precursor of Thr and Lys. These two amino acids were again ^13^C-labelled and hence *de novo* synthesised in significant and well reproducible amounts in *EIEC*-1 and *Stm* but not in *EIEC*-2 (Fig. 3A-E, black bars). Since the carbon backbone of Thr is identical to that of Asp, the isotopologue pattern of Thr is expected to be identical to that of Asp which was indeed the case in both strains (Fig. 3A-E, coloured bars). The biosynthesis of Lys follows (as in most bacteria) the diaminopimelic acid pathway and requires, in addition to Asp, pyruvate with the concomitant loss of one C-atom from the latter precursor by decarboxylation. The occurrence of ^13^C_3_- and ^13^C_5_-Lys as main isotopologues is therefore in accord with their generation from ^13^C_3_-Asp (the major ^13^C-Asp isotopologue) and unlabelled or ^13^C_3_-labelled pyruvate, respectively (in the latter case with the loss of ^13^CO_2_). The minor ^13^C_2_-Lys isotopologue may be the product of ^13^C_2_-Asp and unlabelled pyruvate or unlabelled Asp and ^13^C_3_-labelled pyruvate, respectively.

### Amino acids derived from intermediates of the TCA cycle (Glu, Asp, Pro, Thr, Lys)

#### (b) in the glucose/glucose-6P uptake mutants

In the *EIEC*-1 mutant, ^13^C-incorporation into Glu, Asp, Pro, Thr, and Lys was highly reduced as compared to the wild-type strain (Fig. 3A and B, black bars). The ^13^C-isotopologue patterns of Asp and Glu (similar amounts of ^13^C_2_- and ^13^C_3_-Asp, equal amounts of ^13^C_3_- and ^13^C_4_-Glu isotopologues and high amounts of ^13^C_2_-Glu) suggest that the corresponding oxaloacetate and α-ketoglutarate precursors are mainly derived via two rounds of the TCA cycle, fed by ^13^C_2_-acetyl-CoA (Fig. S2). Apparently, PEP carboxylation did not yield oxaloacetate at the same high rates as detected for the wild-type strain.

In the *EIEC*-2 mutant, the already lower ^13^C-incorporation into Glu and Asp (compared to *EIEC*-1) was only about 2-fold reduced compared to the wild-type (Fig. 3C, black bars). The ^13^C-isotopologue patterns of these two amino acids were similar to those of the *EIEC*-1 mutant, suggesting a similar generation of Glu and Asp in the *EIEC*-2 mutant, i.e. by channelling ^13^C_2_-acetyl-CoA through up to two rounds of the TCA cycle. There was an equally strong reduction of the ^13^C_3_-isotopologue of Asp in this mutant (compared to the wild-type strain) as in the corresponding *EIEC*-1 mutant, suggesting that generation of oxaloacetate by PEP carboxylation does also not occur in the *EIEC*-2 mutant.

In the *Stm* mutant, ^13^C-incorporation into Glu, Pro and Asp was less than 50% reduced, while ^13^C-incorporation into Thr and Lys was more than 70% lower than in the wild-type strain (Fig. 3D and E, black bars). The ^13^C-isotopologue patterns of Glu and Asp (equal amounts of ^13^C_2_- and ^13^C_3_-Asp, equal amounts of ^13^C_3_- and ^13^C_4_-Glu) resembled those of the two *EIEC* strains and suggest that the corresponding ^13^C_3_-labelled oxaloacetate and α-ketoglutarate precursors were again generated by channelling ^13^C_2_-acetyl-CoA through two rounds of the TCA cycle (Fig. S2). The major ^13^C-isotopologue of the weakly labelled Lys was ^13^C_3_-Lys, in contrast to the wild-type strain where more ^13^C_5_- than ^13^C_3_-Lys isotopologue was generated (see above). The preferential generation of the ^13^C_3_-Lys isotopologue might involve ^13^C_2_- and ^13^C_3_-Asp as precursors.

### Amino acids requiring intermediates of the pentose-phosphate cycle (His, Phe, Tyr)

#### (a) in the wild-type strains

^13^C-labelled His was produced in *EIEC*-1 and *Stm* in low (in *EIEC*-2 very low), but reproducible amounts (Fig. 3A-D, black bars; note that the scale for His, Gly, Thr, Phe, and Tyr differs from that for Ala, Asp, Glu, Ser, Val, and Lys). His biosynthesis requires phosphoribosyl pyrophosphate (PRPP) produced in the pentose phosphate cycle, and ATP which contributes one C-atom and the two N-atoms to the imidazol ring of His. In all strains, the major His isotopologue was ^13^C_5_-His (purple bar in Fig. 3A-D) which may be generated from unlabelled ATP and ^13^C_5_-labelled PRPP. The occurrence of ^13^C_5_-PRPP requires its direct synthesis from [U-^13^C_6_]glucose by the pentose phosphate cycle, again reflecting the use of glucose as major carbon source in all three strains (Fig. S1). The minor ^13^C-His isotopologues, which were different in the three strains, are also of interest: the occurrence of ^13^C_6_-His (orange bar) in *EIEC*-1 and *Stm* indicates that one additional ^13^C-atom derives from ^13^C-labelled ATP. This provides evidence for *de novo* biosynthesis of purines in these two intracellular enterobacteria (more in *Stm* than in *EIEC*-1; see Fig. 3A, B compared to D). In *EIEC*-2, there is an additional ^13^C_2_-His isotopologue (yellow bar). The ^13^C_2_-label can derive from ^13^C_2_-labelled PRPP suggesting that in *EIEC-*2 part of PRPP does not directly come from [U-^13^C_6_]glucose but rather from glucose produced by gluconeogenesis from a C_3_-substrate. The almost equal amounts of ^13^C_5_- and ^13^C_2_-His in *EIEC*-2 may therefore be taken as further evidence that this strain uses, in addition to glucose, a C_3_-substrate (most likely glycerol – see Ser), but not glucose-6P for its intracellular metabolism.

Phe and Tyr require for their biosynthesis two molecules of PEP and one molecule of erythrose-4P, the latter representing a product of the pentose phosphate cycle. Both aromatic amino acids were ^13^C-labelled in *EIEC*-1 at low, but reproducible amounts (Fig. 3A and B, black bars). In *Stm*, Phe (but not Tyr) was ^13^C-labelled at a similar rate as in *EIEC*-1 (Fig. 3D). The major isotopologues were ^13^C_9_-species in both strains (blue bars), suggesting their generation from two molecules of ^13^C_3_-PEP and one molecule of ^13^C_4_-erythrose-4P (with the loss of one ^13^C-atom of PEP by decarboxylation). ^13^C_4_-Erythrose-4P can again only derive directly from [U-^13^C_6_]glucose by the action of a transketolase in the pentose phosphate pathway. In *EIEC*-2, ^13^C-labelled Tyr and Phe were not reproducibly observed.

### Amino acids requiring intermediates of the pentose-phosphate cycle (His, Phe, Tyr)

#### (b) glucose/glucose-6P uptake mutants

A significant ^13^C-incorporation into Phe, Tyr and His did not occur in any of the three mutants, suggesting either the failure of *de novo* synthesis of these amino acids in the absence of glucose, or a lack of ^13^C-labelled intermediates of the pentose phosphate cycle due to production of glucose by gluconeogenesis from unlabelled or only weakly ^13^C-labelled carbon substrates utilised in the absence of [U-^13^C_6_]glucose uptake.

### Amino acids taken up by the intracellular pathogens from the host cells

**Figure 4:**
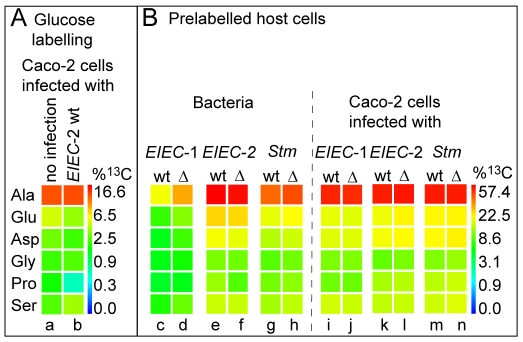
^13^C-Incorporation into amino acids of host cells (Caco-2) and intracellular bacteria (see also Table S5). (A) ^13^C-labelled amino acids in Caco-2 cells (not infected) grown in the presence of 10 mM [U-^13^C_6_]glucose for 6h (lane a) and with infection by *EIEC*-2 (infection by *EIEC*-1 led to similar results) (lane b). (B) ^13^C-labelled amino acids in Caco-2 cells and bacteria when the Caco-2 cells were grown for 10 generations in the presence of 10 mM [U-^13^C_6_]glucose and then infected with the bacteria. The data shows the uptake of the 6 labelled host cell amino acids by the three intracellular bacterial wild-type strains (wt) and the corresponding glucose/glucose-6P uptake mutants (indicated as Δ) during the 6.5 or 8.5 h period of infection (lanes c-h). Labelled amino acids of the Caco-2 host cells 6.5 or 8.5 h after infection by the wild-type strains (wt) or their glucose/glucose-6P uptake mutants (Δ) (lanes i-n). Colours indicate the amount of ^13^C-incorporation (% ^13^C) according to a quasi logarithmic scale.

The largest portion of each amino acid isolated from the bacteria after infection is unlabelled (Table S4) indicating that the major part of each amino acid is either synthesised from unlabelled carbon substrate(s) or imported from the host cell. Unlabelled host cell amino acids could be either taken up by the host cell from the culture medium or generated by proteolytic degradation of unlabelled host cell proteins. To answer the question whether amino acids from the host cell are equally accessible to cytosolic and phagosomal bacteria, the Caco-2 cells were ^13^C-prelabelled prior to infection (by growing Caco-2 cells in RPMI medium supplemented with 10% FCS and 10 mM [U-^13^C_6_]glucose for ten generations). The ^13^C-labelled host cells were then infected (in the presence of 10 mM unlabelled glucose) with the two *EIEC* and the *Stm* wild-type strains as well as the glucose/glucose-6P uptake mutants for 6.5 or 8.5 h. We found in all three intracellular pathogens only those amino acids ^13^C-labelled (Ala, Glu, Asp, Ser, Gly, and Pro) which were also ^13^C-enriched in the host cells (Fig. 4B). Moreover, the ^13^C-isotopologue profiles of the bacterial amino acids were identical with those from the host cells (Table S4). This suggests that these amino acids are taken up and directly incorporated into bacterial protein without any significant catabolic turnover by the bacterial metabolism. However, their amounts showed significant, strain-specific differences. Amino acid uptake was lowest by the *EIEC*-1 wild-type strain and highest by the *EIEC*-2 strain (Fig. 4B, lanes c and e; Table S5). The amino acid uptake rate of the glucose/glucose-6P uptake mutant of *EIEC*-1 was considerably higher (by up to 50%, depending on the amino acid) than that of the corresponding wild-type strain (lanes c and d, Table S5), while the glucose/glucose-6P uptake mutant of *EIEC*-2 was even slightly lower (by about 10%) than that of the wild-type strain (lanes e and f, Table S5). The amount of labelled amino acids taken up by *Stm* (lane g) was in between that of the two *EIEC* strains while the *Stm* glucose/glucose-6P uptake mutant showed again a slight but reproducible increase (about 13%) in the uptake of host cell-derived amino acids compared to the wild-type strain (lanes g and h). In summary, these data show (i) that cytosolic as well as phagosomal bacteria have access to host cell amino acids and (ii) that the amount of host cell-derived amino acids taken up by the three intracellular enterobacterial strains is reverse to the amount of amino acids *de novo* synthesised by these bacteria.

### Is the metabolism of Caco-2 host cells affected by the infection of the intracellular pathogens?

To determine the effect of the infection by the studied intracellular pathogens on the carbon metabolism of the host cells, we compared the ^13^C-incorporation into the *de novo* synthesised amino acids of the uninfected and infected Caco-2 host cells. Both, uninfected Caco-2 cells as well as Caco-2 cells infected by the three strains, incorporated ^13^C-label deriving from [U-^13^C_6_]glucose into six non-essential amino acids with similar rates. Excess ^13^C-label was about: for Ala 11%, for Glu 3%, for Asp 2%, for Ser 3%, for Gly 2%, and for Pro 1%, as shown for the *EIEC*-2 strain in Fig. 4A and Table S5. Among these amino acids, Ala, Glu, Asp and Ser derive from intermediates of glycolysis or the TCA cycle, while Gly and Pro are products of Ser and Glu, respectively. The large amount of *de novo* synthesised Ala is probably due to the absence of this amino acid in the used RPMI culture medium which contains all other amino acids in concentrations of about 0.1 to 2 mM (see also Table S1). The fact that the ^13^C-excess values of uninfected and infected Caco-2 cells are virtually identical (Fig. 4A, lanes a and b) suggests that the infection by the three pathogens does not significantly affect the glucose uptake and metabolite fluxes along glycolysis and the TCA cycle of the host cells.

Not only the rate of ^13^C-incorporation, but also the ^13^C-isotopologue patterns of these amino acids remained essentially unaltered by the infections (Fig. 3F; Table S4). Note that these patterns were quite different from those of the corresponding amino acids of the pathogens indicating that the procedure applied for the separation of host cells and bacteria worked well. The ^13^C-isotopologue patterns of the amino acids in part *de novo* synthesised by the host cells are in agreement with the assumption that glucose is catabolised mainly via glycolysis and the TCA cycle as indicated by the main ^13^C-isotopologues of Ala (^13^C_3_-Ala), Asp (^13^C_2_-Asp) and Glu (^13^C_2_-Glu). The occurrence of the ^13^C_3_-Asp isotopologue and of the ^13^C_3_- and ^13^C_4_-Glu isotopologues at equal amounts (especially observed in the faster proliferating Caco-2 cells) suggests that the host cell TCA cycle undergoes up to two rounds during the infection.

## Discussion

We have analysed by ^13^C-isotopologue profiling the intracellular carbon metabolism of enteroinvasive *E. coli* (*EIEC*; closely resembling *Shigella* species), and *S. enterica* Serovar Typhimurium (*S*. Typhimurium). These two intracellular enterobacterial pathogens replicate in two different compartments of the infected cells, namely the cytosol and the *Salmonella*-containing-vacuole (SCV), respectively. Hence, they may differently interfere with the metabolism of the host cells.

**Figure 5:**
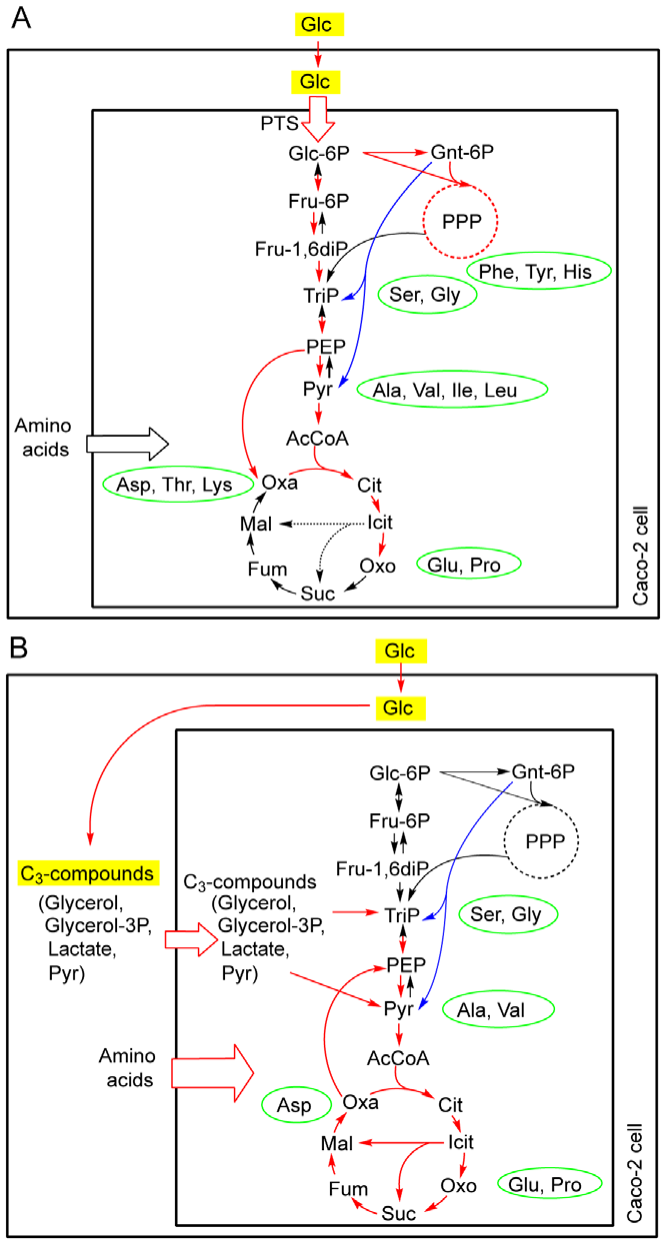
Proposed catabolic pathways and metabolite fluxes employed by the enterobacterial strains replicating in Caco-2 cells. Amino acids (in part) *de novo* synthesised by the bacteria are encircled in green. The import of ^13^C-labelled amino acids from the host cell is indicated by arrows, the magnitude of which roughly reflects the relative amount of imported amino acids under the conditions (A) and (B). *EIEC*-1 wild-type strain and *Stm* follow the metabolic pathways shown in A (red arrows) which is mainly fed by glucose (indicated in yellow), while the glucose/glucose-6P uptake mutants essentially follow the metabolic pathways shown in B (red arrows) mainly fed by C_3_-substrates. *EIEC*-2 appears to combine the pathways of model A and B.

Under the applied culture conditions, all three enterobacterial strains under study take up glucose (but not glucose-6P) directly from the host cell (however with strain-specific efficiencies) and catabolise it mainly through glycolysis and/or the Entner-Doudoroff pathway followed by the TCA cycle (Fig. 5A and Fig. S1). This conclusion is based on the observation that the bacterial mutants impaired in glucose uptake show strong (strain-dependent) reduction of ^13^C-incorporation into amino acids, while all mutants impaired in glucose-6P transport exhibit the same ^13^C-incorporation into all amino acids as the corresponding wild-type strains when growing within Caco-2 cells. Glucose uptake mutants of the three pathogens under study were generated by deleting the *ptsG* and *manXYZ* operons encoding the major PTS glucose permeases of *E. coli* and *S.* Typhimurium [37], [38], [39]. One has to consider, however that the PtsG permease is able to also transport other carbohydrates, such as glucosamine, N-acetylglucosamine, fructose, and α-glucosides, albeit at much lower efficiency than glucose [7], [39]. In principle, these latter carbohydrates could be generated from [U-^13^C_6_]glucose by the host cells, but due to their presumably low cellular concentrations it is very unlikely that a significant portion of the ^13^C-incorporation into the bacterial amino acids derives from these carbohydrates. The major substrate for ManXYZ is mannose. Hence, the uptake of host cell-derived ^13^C-mannose cannot be strictly excluded, but is also unlikely since the Δ*manXYZ* mutant (strongly impaired in mannose transport) shows the same ^13^C-incorporation into the amino acids as the wild-type strain (data not shown).

Uptake of glucose-6P was abolished in all three enterobacterial pathogens by the deletion of *uhpT*. However, the UhpT permease can not only accept ^13^C-glucose-6P as substrate, but also other ^13^C-labelled phosphorylated sugars, such as glucose-1P, mannose-6P, fructose-6P and ribose-5P [40], [41] which may also be formed from [U-^13^C_6_]glucose in the host cells. Since the *uhpT* mutants of all three strains show similar rates of ^13^C-incorporation into the amino acids as the corresponding wild-type strains, not only glucose-6P but also these additional phosphorylated carbohydrates can be excluded as major carbon substrates for the three enterobacterial strains studied.

The cytosolically replicating *EIEC* strains have direct access to [U-^13^C_6_]glucose that is transported from the external culture medium into the cytosol of the Caco-2 cells by the sodium-dependent D-glucose co-transporter SGLT1 residing in endosomes and by the apical GLUT2, a transporter allowing facilitated diffusion of external D-glucose into the cytosol [33]. Although glucose can be efficiently taken up by Caco-2 cells as by all cancer cells (see also below), the cellular pool of free glucose is probably low (see also Fig. 1) since glucose is quickly converted to glucose-6P and channelled into the glycolytic pathway and the pentose phosphate cycle [42], [43]. High-affinity glucose transporters may therefore be required by the intracellular bacteria to take up sufficient amounts of glucose for feeding their intracellular carbon metabolism. Differences in the affinity of the major glucose transporters (e.g. PtsG) could therefore be the reason for the observed striking difference in the efficiency by which the two *EIEC* strains utilise glucose although they grow in the same cytosolic environment (cf. models A and B in Fig. 5 and Figs. S1 and S2). The relatively low glucose affinity of the major glucose-transporting PTS in *Listeria monocytogenes* (compared to that of *E. coli* PtsG; A. Götz, unpublished) may also explain why *L. monocytogenes* uses glycerol rather than glucose as a major carbon substrate for its intracellular carbon metabolism when replicating in the cytosol of macrophages [30].

On the basis of our data, gluconate can be ruled out as major carbon substrate for all three enterobacterial strains under the applied conditions, since^ 13^C-labelled gluconate formed by the host cells from [U-^13^C_6_]glucose could still be taken up by the Δ*ptsG, manXYZ* mutants since the gluconate transporter (GntT) remains fully functional in these mutants. If ^13^C-gluconate acts as a major carbon source, the ^13^C-isotopologue patterns of the *de novo* synthesised amino acids should therefore be similar in these mutants as in the wild-type strains which definitely is not the case. This conclusion differs form an earlier suggestion that gluconate may be a preferred carbon source for intracellular *S*. Typhimurium. This suggestion is based on the up-regulation of *gntT* observed by transcript profiling of intracellularly grown *S*. Typhimurium [44]. The *gntT* gene similar as the *uhpT* gene is under glucose catabolite repression control [45] and the up-regulation of these genes by intracellular *S*. Typhimurium probably reflects the lower glucose concentration in the host cells compared to the *in vitro* culture media used for growing S. Typhimurium from which the reference RNA was isolated. This may also explain the observed down-regulation of the glycolysis and TCA genes in intracellularly grown S. Typhimurium observed in the same transcriptome analysis [44].

The utilisation of glucose by *S*. Typhimurium (Fig. 5A and Fig. S1) residing in the membrane-surrounded SCV is rather unexpected if one assumes that glucose has to be transported from the host cells' cytosol across the SCV membrane to reach the bacteria. Possibly, the endosomal SGLT1 as major glucose transporter of Caco-2 cells [33] may fuse with the SCV membrane thus providing sufficient amounts of [U-^13^C_6_]glucose to the SCV in the Caco-2 cells. The glucose concentration in the SCV - at least of some host cells - seems to be even high enough to activate non-PTS glucose transporters of *S*. Typhimurium [15].

In all glucose uptake mutants, fewer amino acids acquire ^13^C-label. The incorporation rates are highly reduced and the ^13^C-isotopologue profiles are different for most amino acids as compared to the data from the respective wild-type strains. This change is apparently accompanied by an increased uptake of amino acids from the host cells (cf. models A and B in Fig. 5 and Figs. S1 and S2). Significantly enhanced degradation of amino acids in these mutants does not seem to occur since the ^13^C-prelabelled amino acids taken from the host cells retain their (host cell)-specific ^13^C-isotopologue patterns in the bacteria, suggesting that these amino acids are directly incorporated into protein without significant metabolic turnover. The ^13^C-isotopologue patterns of the amino acids *de novo* synthesised in the mutants rather suggest that they now preferentially utilise ^13^C_3_-substrates (presumably glycerol, pyruvate or lactate) generated in the host cells from [U-^13^C_6_]glucose (model B in Fig. 5 and Fig. S2). Carbon compounds (mainly) degraded to ^13^C_2_-acetyl-CoA, such as fatty acids can be excluded as glucose substitute on the basis of the observed ^13^C-isotopologue patterns of certain amino acids, especially Ala. Even the three enterobacterial wild-type strains may use with a strain-dependent extent ^13^C_3_-substrates in addition to glucose, since they apparently rely (in part) on gluconeogenesis which especially becomes evident by the ^13^C-isotopologue pattern of Ser.

By our experimental approach (we analyse the bacterial metabolism only at one time point, i.e. 6.5 or 8.5 h post infection, respectively), we cannot rigorously distinguish whether the intracellular bacterial wild-type strains metabolise these carbon substrates simultaneously or successively. However, external glucose appears to be present in excess throughout the infection period (less than 10% of the external glucose is consumed at the time when the intracellular bacteria are harvested). For this reason, simultaneous utilisation of glucose and a ^13^C_3_-substrate seems to be rather likely.

The largest portion of all amino acids in both types of intracellular bacteria (cytosolic and phagosomal) is unlabelled, indicating that most amino acids are imported from the host cells. For *S*. Typhimurium, this fact shows that amino acids as anabolic monomers can also be efficiently transported across the membrane of the SCV. These amino acids are probably mainly taken up by the host cells from the culture medium (containing all amino acids except Ala) but may in part also originate from unlabelled host cell protein by proteolytic turnover (see e.g. Ala). The latter possibility is also suggested by the observation that substantial amounts of host cell-derived ^13^C-labelled (“non-essential”) amino acids are imported into the intracellular bacteria when the host cell protein is prelabelled with [U-^13^C_6_]glucose for several generations prior to infection. Surprisingly, even amino acids “essential” for the host cells, like Ile, Leu, Phe, Tyr are not or only to a small part *de novo* synthesised by the intracellular bacteria (as judged by the missing or only low ^13^C-label in these amino acids isolated from the bacteria) suggesting that even these amino acids (present in the culture medium) are transported through the host cells into the intracellular bacteria. However, there are strain-specific differences between the three enterobacterial wild-type strains and their glucose transport mutants in the ratio of the amino acids *de novo* synthesised and those taken up from the host cells. From these differences one may conclude that the utilisation of energy-rich glucose by the intracellular bacteria allows increased *de novo* synthesis of amino acids (possibly also other anabolic monomers) (model A in Fig. 5 and Fig. S1), while consumption of the less energy-rich C_3_-substrates needs increased uptake of amino acids from the host cells (model B in Fig. 5 and Fig. S2). This balance between the bacterial anabolic metabolism and the utilised carbon source may be the reason for the still relatively efficient intracellular replication of the glucose uptake mutants. This metabolic flexibility may also be crucial for the survival and persistence of *S*. Typhimurium during systemic infections in mice where the bacteria are encountering conditions with varying nutrient supply [16], [17], [46], [47].

Finally, the infection of the Caco-2 host cells neither by the *EIEC* strains nor the *S*. Typhimurium isolate causes significant changes in the carbon metabolism of the host cell as indicated by the unchanged ^13^C-patterns of the amino acids deriving from intermediates of glycolysis (Ala, Ser, Gly) or the TCA cycle (Glu, Pro, Asp). This is different to recent findings in mammalian cells infected with human cytomegalovirus (HCMV) [48] or *Chlamydia pneumoniae* [49]. Infection with HCMV markedly up-regulates flux through much of the central carbon metabolism, including glycolysis and the TCA cycle of infected MRC-fibroblasts. *C. pneumoniae* has an additive effect on hypoxia-inducible factor-1 alpha (HIF-1α) stabilization resulting in enhanced glucose uptake of Hep-2 cells during the early phase of infection.

In conclusion, the data indicate that glucose is a preferred carbon source for the intracellular metabolism of *Escherichia coli* (*EIEC* HN280) and *Salmonella enterica* Serovar Typhimurium isolate 14028. *EIEC* 4608-58 also uses glucose but appears to simultaneously utilise a C_3_-substrate, probably glycerol. In all strains under study, the prime carbon source glucose can be replaced, if it becomes unavailable, by alternative C_3_-substrates providing less energy. The use of these alternative low-energy nutrients seems to be counterbalanced by an increased uptake of amino acids (and possibly also of other anabolic monomers) from the host cells.

Our experiments are done at an external glucose concentration resembling the physiological concentrations in the human blood and the intestines. However, one should keep in mind that our results are obtained with a tumour cell line as a model host cell. Tumour cells are known to exhibit a specific metabolism which is characterise d by an increased uptake of glucose and by enhanced glycolysis (“Warburg-effect” - for a recent review see [43]). Only further studies with actual target cells, tissues and animals will show whether our conclusions drawn from these model studies are also valid under infection conditions.

## Materials and Methods

### Bacterial strains and cell line

*EIEC* HN280 and *EIEC* 4608-58 were gifts from M. Nicoletti (Chieti, Italy) and P. Sansonetti (Paris, France) [1], [50]. *S*. Typhimurium 14028 was obtained from M. Hensel (Erlangen, Germany). Extra- and intracellular growth properties of these strains and mutants are summarised in Table S2. The Caco-2 cell line (ACC 169) was obtained from the German Collection of Microorganisms and Cell Cultures (DSMZ, Braunschweig, Germany). Caco-2 cells were cultured at 37°C and 5% CO_2_ in RPMI 1640 medium supplemented with 10% FCS.

### Bacterial growth conditions prior to infection

The *EIEC* strains were grown overnight in 20 ml Tryptic Soy Broth (TSB) at 30°C and then diluted 1∶100 (v/v) in 20 ml of fresh TSB and further incubated for 2 h at 37°C with aeration. An overnight culture of *Stm* 14028 grown in 20 ml LB with 0.5% NaCl at 37°C was diluted 1∶100 (v/v) into 20 ml of fresh LB with 1% NaCl and further shaken for 2.5 h at 37°C. When the cultures reached a late logarithmic growth phase (OD_600_≈1), bacteria were diluted in RPMI 1640 containing 10% FCS and added to the Caco-2 cells at different multiplicities of infection (MOI) (see below).

### Infection of Caco-2 cells and ^13^C-labelling

1×10^7^ cells were seeded into 20 ml cell culture medium in a 175 cm^2^ cell culture flask and grown for 2 days to obtain a 90% confluent cell layer containing about 2×10^7^ cells. Ten flasks were used in the infection experiments for each strain. For infection, the culture medium was replaced by 20 ml of RPMI 1640 medium containing 10% FCS and the bacteria. Due to different invasion and replication rates of the bacteria, the MOI was adjusted to 20 bacteria per Caco-2 cell (for the *EIEC* HN280 wild-type strain and the isogenic Δ*uhpT* mutant) and to 100 (for all other strains) to obtain a total of approximately 10^8^ colony forming units (cfu) for *EIEC* 4608-58 and 10^9^ cfu for *EIEC* HN280 and *Stm* 14028 at the time point of harvest. Infection was allowed to proceed for 1 h. Then, the medium was withdrawn and replaced by RPMI 1640 medium with 10% FCS containing 100 µg/ml gentamicin to kill extracellular bacteria prior to ^13^C-labelling. This was defined as time point zero hour post infection (h p.i.). After 30 min incubation, this medium was again replaced by fresh RPMI 1640 medium containing 10% FCS, 100 µg/ml gentamicin and 10 mM [U-^13^C_6_]glucose. After 30 min, the gentamicin concentration was lowered to 20 µg/ml. The infected cells were washed with 10 ml of ice-cold PBS 6.5 h p.i. (for the two *EIEC* strains) or 8.5 h p.i. (for *Stm* 14028), and lysed in 10 ml of ice-cold distilled water containing 0.1% TritonX-100, 20 mM Na-azide, 5 µg/ml tetracycline and 50 µg/ml chloramphenicol for 20 min. Host cell debris and nuclei were removed by three centrifugation steps at 200×g for 5 min at 4°C. The supernatant containing the bacteria and the soluble host cell material was centrifuged at 5500×g and 4°C for 15 min to pellet the bacteria. The resulting supernatant was stored at −80°C and used as probe for the analysis of the host cell amino acids (derived from soluble host cell protein). The bacteria were washed with 5 ml of RIPA-buffer (150 mM NaCl, 50 mM Tris, 0.1% SDS, 0.5% deoxycholate, 1% Igepal non-idet P-40) and centrifuged again. The resulting pellet was resuspended in 500 µl of PBS and stored at −80°C. This fraction was used as probe for the analysis of the ^13^C-labelled bacterial amino acids. The contamination of the host cell protein fraction with bacterial proteins and vice versa was less than 10% on the basis of the differential ^13^C-labelling patterns.

### Determination of glucose consumption by the host cells and the intracellular bacteria with ^14^C-labelled glucose

Infection of Caco-2 cells with the three enterobacterial wild-type strains was carried out as described above. After the internalization period the remaining extracellular bacteria were killed by treatment with 100 µg/ml gentamicin (0 h p.i.) for 30 min; then the medium was replaced by 20 ml fresh RPMI 1640 containing 10% FCS, 20 µg/ml gentamicin and 200 µmol [U-^14^C]glucose (specific activity, 0.5 MBq/mmol). The infected host cells were further incubated for 6 h (for both *EIEC strains*) or 8 h (for *Stm* 14028). Thereafter, the cells were washed 4 times with 15 ml PBS, lysed as described above, and the cfu were determined by plating serial dilutions on LB-agar plates. Host cell and bacterial fractions were separated as described above. Bacteria were resuspended in 1 ml PBS and the cfu were determined again. ^14^C-Label in each fraction was measured in a scintillation counter and the consumption of ^14^C-glucose by the host cells and the bacteria was calculated (on a molar basis) by the incorporated ^14^C-label.

### ^13^C-Isotopologue profiling

Bacterial and host cell protein fractions were hydrolyzed with 6 M HCl for 24 h at 105°C and the resulting amino acids were converted into their tert-butyl-dimethylsilyl (TBDMS)-derivatives as described earlier [30]. ^13^C-Excess and the isotopologue composition was determined as described earlier [30], [51] using a GC/MS Shimadzu QP 2010 Plus.

## Supporting Information

Figure S1 Metabolic model for enteroinvasive *Escherichia coli* (*EIEC* HN280 and *EIEC* 4608-58) and *Salmonella enterica* Serovar Typhimurium (*Stm* 14028) wild-type strains replicating in Caco-2 cells in the presence of 10 mM [U-^13^C_6_]glucose. The bacteria are indicated by the large boxes. Host cell metabolites and reactions are shown outside these boxes. Contiguous ^13^C-label is indicated by red bars. Single labelled isotopologues are indicated by red dots. Metabolic flux observed under the various conditions is indicated by blue arrows. The relative flux rates (on the basis of the detected ^13^C-enrichments in amino acids) are symbolised by the widths of the arrows. ^13^C-enriched amino acids detected are shown in boxes. The numbers indicate the molar contributions (%) of the major ^13^C-isotopologues detected by MS. The positional ^13^C-label distributions (as indicated by the bars and dots) are predicted on the basis of the metabolic reactions in the model. Glucose is utilized by the Entner-Doudoroff pathway. Notably, degradation of [U-^13^C_6_]glucose by glycolysis or the pentose phosphate pathway leads to identical labelling patterns. In *EIEC* 4608-58, C_3_-compounds are utilised in addition to glucose (indicated by the green arrows). The labelling patterns detected in amino acids suggest that the citrate cycle is mainly used for the formation of alpha-ketoglutarate and that oxaloacetate mainly derives by PEP carboxylation.
(16.52 MB TIF)

Figure S2 Metabolic model for delta *ptsG, manXYZ, uhpT* mutants (impaired in the uptake of glucose and glucose 6-phosphate) of enteroinvasive *Escherichia coli* (*EIEC* HN280 and *EIEC* 4608-58) and *Salmonella enterica* Serovar Typhimurium (*Stm* 14028) replicating in Caco-2 cells in the presence of 10 mM [U-^13^C_6_]glucose. The bacteria are indicated by the large boxes. Host cell metabolites and reactions are shown outside these boxes. Contiguous ^13^C-label is indicated by red bars. Single labelled isotopologues are indicated by red dots. Metabolic flux observed under the various conditions is indicated by blue arrows. The relative flux rates (on the basis of the detected ^13^C-enrichments in amino acids) are symbolised by the widths of the arrows. ^13^C-enriched amino acids detected are shown in boxes. The numbers indicate the molar contributions (%) of the major ^13^C-isotopologues detected by MS. The positional ^13^C-label distributions (as indicated by the bars and dots) are predicted on the basis of the metabolic reactions in the model. This model is based on the lack of efficient glucose uptake. In this case, intracellular metabolism is shifted to an increased uptake of C_3_-compounds which may be shuffled into the central carbon metabolism at the level of pyruvate. alpha-Ketoglutarate as well as oxaloacetate are derived in this case from the citrate cycle. Labelling patterns due to two rounds of citrate cycling are given in purple. Under these conditions, amino acids are imported from the host cells more efficiently (indicated by the arrow widths).
(14.04 MB TIF)

Table S1 Composition of the standard RPMI 1640 medium used in the present study. (Gibco: http://www.invitrogen.com/site/us/en/home/support/Product-Technical-Resources/media_formulation.116.html).
(0.06 MB DOC)

Table S2 Generation times of the studied enterobacterial pathogens during extra- and intracellular growth. The doubling times (DT in min) during growth of the bacteria in RPMI 1640, supplemented with 10 mM glucose (Glc), mannose (Man) or glucose 6-phosphate (Glc6P), or in Caco-2 cells were determined by measuring the bacterial cell density (at OD600) and the colony forming units (cfu), respectively. Also shown (right to the doubling times) are the highest bacterial densities reached during growth. Note that there is a low growth background of about OD600  =  0.2 even in the absence (w/o) of Glc, Man or Glc6P. Doubling times during infection of Caco-2 cells were averaged from time point 1 to 6 h post infection (p.i.). for the *EIEC* strains and from 0 to 8 h p.i. for *Stm* 14028. The construction of the mutants and further details of their properties are published in [32].
(0.06 MB DOC)

Table S3 ^13^C-Excess (%) per C-atom in amino acids from experiments a-β, for details see Figure 2.
(0.10 MB DOC)

Table S4 ^13^C-Isotopologue abundance of amino acids from all experiments in mol%. The labelling pattern is given in terms of XY-groups (Römisch-Margl et al., 2007). This notation is based on digits for each C-atom. The first digit represents C-1, the second C-2, etc.; 1 signifies ^13^C, 0 signifies ^12^C, and X and Y signify either ^13^C or ^12^C. While the labelling status of X is totally undefined, the overall number of ^13^C labelled atoms of Y is known and written outside the brackets. Two independent labelling experiments were analyzed if not indicated otherwise.
(1.38 MB DOC)

Table S5 13C-Excess (%) per C-atom in amino acids from experiments a-n, for details see Figure 4.
(0.04 MB DOC)
