# Supplementary figures and images for: Carbon Metabolism of Enterobacterial Human Pathogens Growing in Epithelial Colorectal Adenocarcinoma (Caco-2) Cells
Source: PLoS One. 2010 May 11;5(5):e10586. doi: 10.1371/journal.pone.0010586 (PMC2868055; doi:10.1371/journal.pone.0010586)

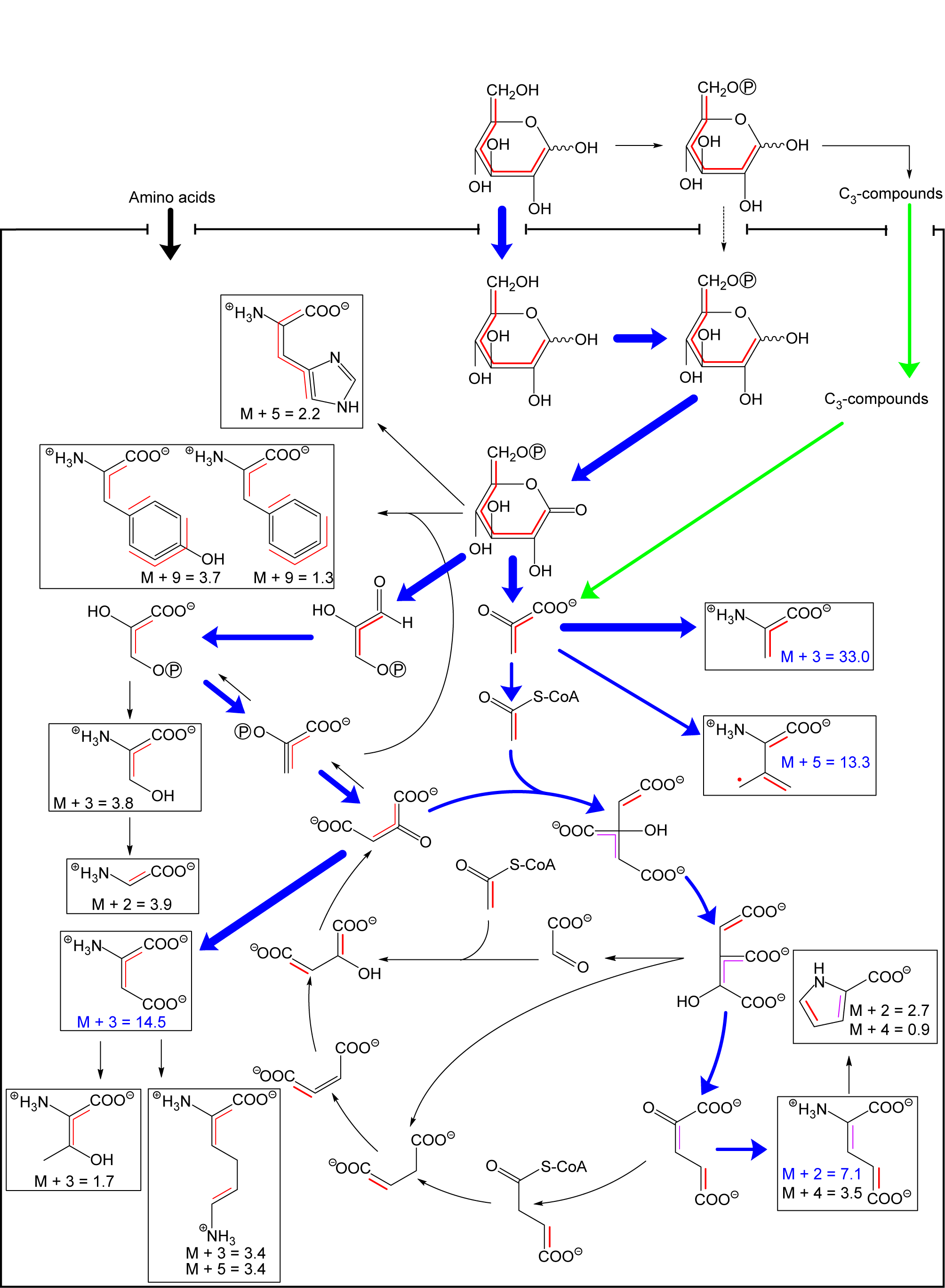

Supplement: Figure S1 — Metabolic model for enteroinvasive Escherichia coli (EIEC HN280 and EIEC 4608-58) and Salmonella enterica Serovar Typhimurium (Stm 14028) wild-type strains replicating in Caco-2 cells in the presence of 10 mM [U-13C6]glucose. The bacteria are indicated by the large boxes. Host cell metabolites and reactions are shown outside these boxes. Contiguous 13C-label is indicated by red bars. Single labelled isotopologues are indicated by red dots. Metabolic flux observed under the various conditions is indicated by blue arrows. The relative flux rates (on the basis of the detected 13C-enrichments in amino acids) are symbolised by the widths of the arrows. 13C-enriched amino acids detected are shown in boxes. The numbers indicate the molar contributions (%) of the major 13C-isotopologues detected by MS. The positional 13C-label distributions (as indicated by the bars and dots) are predicted on the basis of the metabolic reactions in the model. Glucose is utilized by the Entner-Doudoroff pathway. Notably, degradation of [U-13C6]glucose by glycolysis or the pentose phosphate pathway leads to identical labelling patterns. In EIEC 4608-58, C3-compounds are utilised in addition to glucose (indicated by the green arrows). The labelling patterns detected in amino acids suggest that the citrate cycle is mainly used for the formation of alpha-ketoglutarate and that oxaloacetate mainly derives by PEP carboxylation. (16.52 MB TIF) [file pone.0010586.s001.tif]

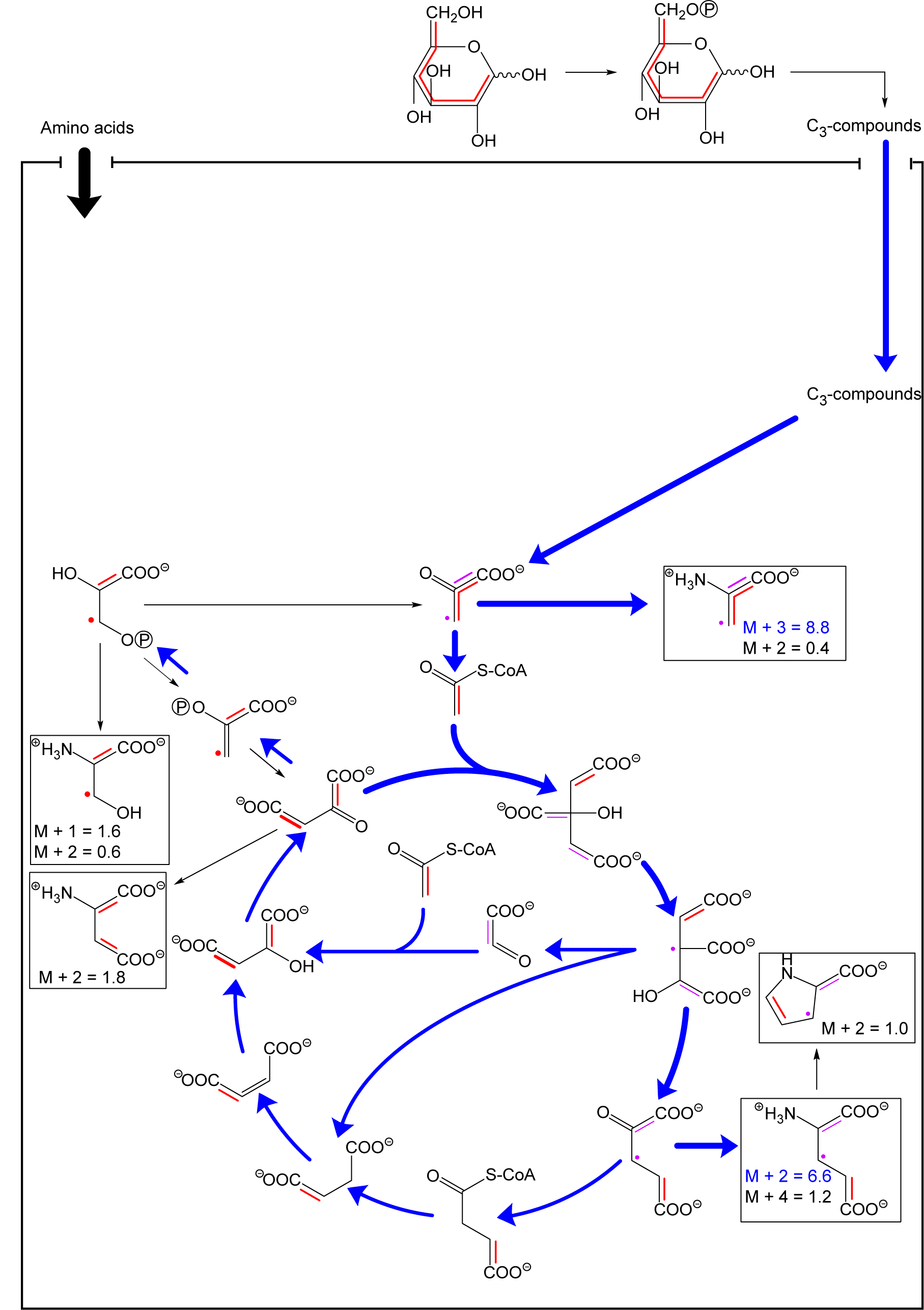

Supplement: Figure S2 — Metabolic model for delta ptsG, manXYZ, uhpT mutants (impaired in the uptake of glucose and glucose 6-phosphate) of enteroinvasive Escherichia coli (EIEC HN280 and EIEC 4608-58) and Salmonella enterica Serovar Typhimurium (Stm 14028) replicating in Caco-2 cells in the presence of 10 mM [U-13C6]glucose. The bacteria are indicated by the large boxes. Host cell metabolites and reactions are shown outside these boxes. Contiguous 13C-label is indicated by red bars. Single labelled isotopologues are indicated by red dots. Metabolic flux observed under the various conditions is indicated by blue arrows. The relative flux rates (on the basis of the detected 13C-enrichments in amino acids) are symbolised by the widths of the arrows. 13C-enriched amino acids detected are shown in boxes. The numbers indicate the molar contributions (%) of the major 13C-isotopologues detected by MS. The positional 13C-label distributions (as indicated by the bars and dots) are predicted on the basis of the metabolic reactions in the model. This model is based on the lack of efficient glucose uptake. In this case, intracellular metabolism is shifted to an increased uptake of C3-compounds which may be shuffled into the central carbon metabolism at the level of pyruvate. alpha-Ketoglutarate as well as oxaloacetate are derived in this case from the citrate cycle. Labelling patterns due to two rounds of citrate cycling are given in purple. Under these conditions, amino acids are imported from the host cells more efficiently (indicated by the arrow widths). (14.04 MB TIF) [file pone.0010586.s002.tif]
