# Supplementary material for: Carbon Metabolism of Enterobacterial Human Pathogens Growing in Epithelial Colorectal Adenocarcinoma (Caco-2) Cells
Source: PLoS One. 2010 May 11;5(5):e10586. doi: 10.1371/journal.pone.0010586 (PMC2868055; doi:10.1371/journal.pone.0010586)
Supplement: Table S1 — Composition of the standard RPMI 1640 medium used in the present study. (Gibco: http://www.invitrogen.com/site/us/en/home/support/Product-Technical-Resources/media_formulation.116.html). (0.06 MB DOC) [file pone.0010586.s003.doc]

**Table S1: Generation times of the studied enterobacterial pathogens during extra- and intracellular growth.**

|  |  | WT | | Δ*uhpT* | | Δ*ptsG*, *manXYZ* | | Δ*ptsG*, *manXYZ*, *uhpT* | |
| --- | --- | --- | --- | --- | --- | --- | --- | --- | --- |
|  | RPMI + | DT | OD_600_ | DT | OD_600_ | DT | OD_600_ | DT | OD_600_ |
| *EIEC* HN280 | w/o |  | 0.2 ± 0.01 |  | 0.2 ± 0.02 |  | 0.2 ± 0.01 |  | 0.1 ± 0.01 |
|  | Glc | 49 ± 1 | 1.5 ± 0.01 | 49 ± 1 | 1.5 ± 0.01 | 118 ± 5 | 0.3 ± 0.01 | 132 ± 15 | 0.2 ± 0.01 |
|  | Man | 85 ± 2 | 0.5 ± 0.05 | 85 ± 1 | 0.6 ± 0.05 | 99 ± 5 | 0.2 ± 0.01 | 107 ± 5 | 0.2 ± 0.01 |
|  | Glc6P | 47 ± 1 | 1.4 ± 0.02 | 123 ± 5 | 0.3 ± 0.01 | 49 ± 3 | 1.2 ± 0.03 | 148 ± 22 | 0.2 ± 0.01 |
|  | Caco-2 | 55 ± 3 |  | 53 ± 1 |  | 68 ± 6 |  | 70 ± 9 |  |
| *EIEC* 4608-58 | w/o |  | 0.2 ± 0.01 |  | 0.2 ± 0.01 |  | 0.2 ± 0.01 |  | 0.2 ± 0.01 |
|  | Glc | 48 ± 0.2 | 1.6 ± 0.02 | 49 ± 0.4 | 1.6 ± 0.01 | 88 ± 2.0 | 0.4 ± 0.05 | 103 ± 3.0 | 0.3 ± 0.01 |
|  | Man | 68 ± 1 | 0.8 ± 0.01 | 68 ± 1 | 0.8 ± 0.01 | 87 ± 3 | 0.2 ± 0.01 | 105 ± 1 | 0.2 ± 0.01 |
|  | Glc6P | 48 ± 1.0 | 1.4 ± 0.02 | 81 ± 0.4 | 1.0 ± 0.02 | 48 ± 1.0 | 1.3 ± 0.02 | 86 ± 2.0 | 1.1 ± 0.03 |
|  | Caco-2 | 47 ± 8 |  |  |  |  |  | 72 ± 7 |  |
| *Stm* 14028 | w/o |  | 0.3 ± 0.01 |  | 0.3 ± 0.01 |  | 0.3 ± 0.01 |  | 0.3 ± 0.01 |
|  | Glc | 38 ± 1 | 1.5 ± 0.01 | 38 ± 1 | 1.5 ± 0.01 | 63 ± 1 | 1.4 ± 0.03 | 61 ± 1 | 1.4 ± 0.03 |
|  | Man | 72 ± 6 | 1.1 ± 0.02 | 68 ± 3 | 1.1 ± 0.07 | 93 ± 4 | 0.4 ± 0.02 | 99 ± 14 | 0.4 ± 0.04 |
|  | Glc6P | 41 ± 2 | 1.4 ± 0.03 | 84 ± 1 | 0.5 ± 0.02 | 42 ± 1 | 1.2 ± 0.01 | 79 ± 9 | 0.5 ± 0.1 |
|  | Caco-2 | 146 ± 18 |  | 135 ± 5 |  | 169 ± 18 |  | 165 ± 24 |  |

The doubling times (DT in min) during growth of the bacteria in RPMI 1640, supplemented with 10 mM glucose (Glc), mannose (Man) or glucose 6-phosphate (Glc6P), or in Caco-2 cells were determined by measuring the bacterial cell density (at OD_600_) and the colony forming units (cfu), respectively. Also shown (right to the doubling times) are the highest bacterial densities reached during growth. Note that there is a low growth background of about OD_600_ = 0.2 even in the absence (w/o) of Glc, Man or Glc6P. Doubling times during infection of Caco-2 cells were averaged from time point 1 to 6 h post infection (p. i.). for the *EIEC* strains and from 0 to 8 h p. i. for *Stm* 14028*.* The construction of the mutants and further details of their properties are published in Götz and Goebel, 2010.
