# Supplementary material for: Carbon Metabolism of Enterobacterial Human Pathogens Growing in Epithelial Colorectal Adenocarcinoma (Caco-2) Cells
Source: PLoS One. 2010 May 11;5(5):e10586. doi: 10.1371/journal.pone.0010586 (PMC2868055; doi:10.1371/journal.pone.0010586)
Supplement: Table S2 — Generation times of the studied enterobacterial pathogens during extra- and intracellular growth. The doubling times (DT in min) during growth of the bacteria in RPMI 1640, supplemented with 10 mM glucose (Glc), mannose (Man) or glucose 6-phosphate (Glc6P), or in Caco-2 cells were determined by measuring the bacterial cell density (at OD600) and the colony forming units (cfu), respectively. Also shown (right to the doubling times) are the highest bacterial densities reached during growth. Note that there is a low growth background of about OD600 = 0.2 even in the absence (w/o) of Glc, Man or Glc6P. Doubling times during infection of Caco-2 cells were averaged from time point 1 to 6 h post infection (p.i.). for the EIEC strains and from 0 to 8 h p.i. for Stm 14028. The construction of the mutants and further details of their properties are published in [32]. (0.06 MB DOC) [file pone.0010586.s004.doc]

**Table S2: Generation times of the studied enterobacterial pathogens during extra- and intracellular growth.**

The doubling times (DT in min) during growth of the bacteria in RPMI 1640, supplemented with 10 mM glucose (Glc), mannose (Man) or glucose 6-phosphate (Glc6P), or in Caco-2 cells were determined by measuring the bacterial cell density (at OD_600_) and the colony forming units (cfu), respectively. Also shown (right to the doubling times) are the highest bacterial densities reached during growth. Note that there is a low growth background of about OD_600_ = 0.2 even in the absence (w/o) of Glc, Man or Glc6P. Doubling times during infection of Caco-2 cells were averaged from time point 1 to 6 h post infection (p.i.) for the *EIEC* strains and from 0 to 8 h p.i. for *Stm* 14028*.* The construction of the mutants and further details of their properties are published in [32].

|  |  | WT | | Δ*uhpT* | | Δ*ptsG*, *manXYZ* | | Δ*ptsG*, *manXYZ*, *uhpT* | |
| --- | --- | --- | --- | --- | --- | --- | --- | --- | --- |
|  | RPMI + | DT | OD_600_ | DT | OD_600_ | DT | OD_600_ | DT | OD_600_ |
| *EIEC* HN280 | w/o |  | 0.2 ± 0.01 |  | 0.2 ± 0.02 |  | 0.2 ± 0.01 |  | 0.1 ± 0.01 |
|  | Glc | 49 ± 1 | 1.5 ± 0.01 | 49 ± 1 | 1.5 ± 0.01 | 118 ± 5 | 0.3 ± 0.01 | 132 ± 15 | 0.2 ± 0.01 |
|  | Man | 85 ± 2 | 0.5 ± 0.05 | 85 ± 1 | 0.6 ± 0.05 | 99 ± 5 | 0.2 ± 0.01 | 107 ± 5 | 0.2 ± 0.01 |
|  | Glc6P | 47 ± 1 | 1.4 ± 0.02 | 123 ± 5 | 0.3 ± 0.01 | 49 ± 3 | 1.2 ± 0.03 | 148 ± 22 | 0.2 ± 0.01 |
|  | Caco-2 | 55 ± 3 |  | 53 ± 1 |  | 68 ± 6 |  | 70 ± 9 |  |
| *EIEC* 4608-58 | w/o |  | 0.2 ± 0.01 |  | 0.2 ± 0.01 |  | 0.2 ± 0.01 |  | 0.2 ± 0.01 |
|  | Glc | 48 ± 0.2 | 1.6 ± 0.02 | 49 ± 0.4 | 1.6 ± 0.01 | 88 ± 2.0 | 0.4 ± 0.05 | 103 ± 3.0 | 0.3 ± 0.01 |
|  | Man | 68 ± 1 | 0.8 ± 0.01 | 68 ± 1 | 0.8 ± 0.01 | 87 ± 3 | 0.2 ± 0.01 | 105 ± 1 | 0.2 ± 0.01 |
|  | Glc6P | 48 ± 1.0 | 1.4 ± 0.02 | 81 ± 0.4 | 1.0 ± 0.02 | 48 ± 1.0 | 1.3 ± 0.02 | 86 ± 2.0 | 1.1 ± 0.03 |
|  | Caco-2 | 47 ± 8 |  |  |  |  |  | 72 ± 7 |  |
| *Stm* 14028 | w/o |  | 0.3 ± 0.01 |  | 0.3 ± 0.01 |  | 0.3 ± 0.01 |  | 0.3 ± 0.01 |
|  | Glc | 38 ± 1 | 1.5 ± 0.01 | 38 ± 1 | 1.5 ± 0.01 | 63 ± 1 | 1.4 ± 0.03 | 61 ± 1 | 1.4 ± 0.03 |
|  | Man | 72 ± 6 | 1.1 ± 0.02 | 68 ± 3 | 1.1 ± 0.07 | 93 ± 4 | 0.4 ± 0.02 | 99 ± 14 | 0.4 ± 0.04 |
|  | Glc6P | 41 ± 2 | 1.4 ± 0.03 | 84 ± 1 | 0.5 ± 0.02 | 42 ± 1 | 1.2 ± 0.01 | 79 ± 9 | 0.5 ± 0.1 |
|  | Caco-2 | 146 ± 18 |  | 135 ± 5 |  | 169 ± 18 |  | 165 ± 24 |  |
