# Supplementary material for: Carbon Metabolism of Enterobacterial Human Pathogens Growing in Epithelial Colorectal Adenocarcinoma (Caco-2) Cells
Source: PLoS One. 2010 May 11;5(5):e10586. doi: 10.1371/journal.pone.0010586 (PMC2868055; doi:10.1371/journal.pone.0010586)
Supplement: Table S3 — 13C-Excess (%) per C-atom in amino acids from experiments a-β, for details see Figure 2. (0.10 MB DOC) [file pone.0010586.s005.doc]

**Table S3: ^13^C-Excess (%) per C-atom in amino acids from experiments a-β, for details see Figure 2**

|  | *EIEC* HN280 | | | | | | Caco-2 cells infected with *EIEC* HN280 | | | | | |
| --- | --- | --- | --- | --- | --- | --- | --- | --- | --- | --- | --- | --- |
|  | a | b | c | d | e | f | g | h | i | j | k | l |
| Ala | 33.32% | 8.27% | 4.85% | 31.40% | 42.49 % | 10.57 % | 5.92 % | 7.49 % | 7.29 % | 6.85 % | 14.85 % | 16.55 % |
| Asp | 14.06% | 1.77% | 1.54% | 12.55% | 17.91 % | 2.10 % | 1.25 % | 2.35 % | 1.60 % | 1.62 % | 2.52 % | 2.07 % |
| Glu | 7.80% | 3.81% | 1.26% | 7.07% | 11.25% | 1.60% | 3.51 % | 1.41 % | 4.58 % | 4.59 % | 6.57 % | 5.58 % |
| Gly | 4.26% | 0.80% | 0.52% | 4.27% | 12.98 % | 1.10 % | 0.54 % | 0.53 % | 0.69 % | 0.74 % | 2.63 % | 2.25 % |
| Pro | 2.01% | 0.18% | 0.30% | 1.99% | 5.10% | 1.02% | 0.70 % | 0.31 % | 1.01 % | 0.54 % | 1.78 % | 2.30 % |
| Ser | 6.09% | 0.99% | 0.94% | 6.37% | 16.44 % | 2.08 % | 1.15 % | 1.11 % | 1.17 % | 1.20 % | 2.82 % | 2.79 % |
| His | 1.88% | 0.09% | 0.27% | 2.47% | 5.20% | 0.20% | 0.19 % | 0.02 % | 0.12 % | 0.28 % | 1.04 % | 0.74 % |
| Ile | 1.11% | 0.61% | 0.61% | 1.00% | 2.00% | 0.22% | 0.52 % | 2.34 % | 0.49 % | 0.51 % | 0.24 % | 0.10 % |
| Leu | 0.08% | 0.01% | 0.01% | 0.06% | 0.32% | 0.03% | 0.09 % | 0.10 % | 0.11 % | 0.11 % | 0.03 % | 0.03 % |
| Lys | 5.88% | 0.00% | 0.15% | 5.71% | 10.65% | 0.61% | 0.10 % | 0.27 % | 0.14 % | 0.14 % | 0.05 % | 0.06 % |
| Phe | 1.59% | 0.32% | 0.00% | 1.46% | 4.50 % | 0.27 % | 0.08 % | 0.75 % | 0.13 % | 0.26 % | 0.00 % | 0.00 % |
| Thr | 2.42% | 0.13% | 0.00% | 1.59% | 4.48 % | 0.10 % | 0.47 % | 0.44 % | 0.48 % | 0.52 % | 0.09 % | 0.07 % |
| Tyr | 4.23% | 0.00% | 0.00% | 3.99% | 7.40 % | 0.12 % | 1.84 % | 4.00 % | 2.03 % | 1.79 % | 0.00 % | 0.00 % |
| Val | 17.49% | 0.52% | 1.13% | 16.33% | 23.73% | 0.92% | 0.09 % | 1.61 % | 0.09 % | 0.14 % | 0.18 % | 0.01 % |

|  | *EIEC* 4608-58 | | Caco-2 cells infected with *EIEC* 4608-58 | |
| --- | --- | --- | --- | --- |
|  | m | n | o | p |
| Ala | 12.38 % | 6.78 % | 13.58 % | 13.24 % |
| Asp | 6.23 % | 1.36 % | 2.21 % | 2.13 % |
| Glu | 3.41% | 1.19% | 4.39 % | 4.01 % |
| Gly | 1.35 % | 0.61 % | 1.85 % | 1.64 % |
| Pro | 0.89% | 1.86% | 1.53 % | 2.36 % |
| Ser | 2.50 % | 1.16 % | 2.17 % | 1.82 % |
| His | 0.97% | 0.26% | 0.11 % | 0.13 % |
| Ile | 0.12% | 0.11% | 0.05 % | 0.06 % |
| Leu | 0.03% | 0.02% | 0.02 % | 0.02 % |
| Lys | 0.61% | 0.16% | 0.13 % | 0.11 % |
| Phe | 0.37 % | 0.27 % | 0.11 % | 0.18 % |
| Thr | 0.41 % | 0.33 % | 0.19 % | 0.43 % |
| Tyr | 0.84 % | 1.01 % | 0.00 % | 0.09 % |
| Val | 2.86% | 0.09% | 0.02 % | 0.07 % |

|  | *Stm* 14028 | | | | | | Caco-2 cells infected with *Stm* 14028 | | | | | |
| --- | --- | --- | --- | --- | --- | --- | --- | --- | --- | --- | --- | --- |
|  | q | r | s | t | u | v | w | x | y | z | α | β |
| Ala | 21.16% | 11.56% | 11.71% | 21.16% | 42.66 % | 11.98 % | 10.07 % | 10.66 % | 9.96 % | 10.40 % | 15.98 % | 14.84 % |
| Asp | 7.29% | 3.88% | 3.87% | 7.71% | 17.28 % | 4.10 % | 2.14 % | 2.30 % | 2.09 % | 2.27 % | 2.89 % | 2.57 % |
| Glu | 6.85% | 3.71% | 3.72% | 7.13% | 14.95 % | 3.80 % | 5.55 % | 5.96 % | 5.64 % | 5.84 % | 5.39 % | 5.64 % |
| Gly | 5.76% | 1.83% | 1.80% | 5.85% | 15.03 % | 1.44 % | 0.99 % | 1.15 % | 1.08 % | 1.10 % | 2.59 % | 2.41 % |
| Pro | 2.67% | 2.21% | 2.23% | 3.16% | 5.28 % | 4.36 % | 1.80 % | 2.24 % | 1.74 % | 1.98 % | 2.50 % | 3.98 % |
| Ser | 6.69% | 2.68% | 2.81% | 6.61% | 22.67 % | 2.22 % | 1.50 % | 1.64 % | 1.57 % | 1.45 % | 3.42 % | 3.51 % |
| His | 2.20% | 1.15% | 0.87% | 2.67% | 6.73 % | 0.00 % | 0.14 % | 0.15 % | 0.28 % | 0.25 % | 0.99 % | 5.19 % |
| Ile | 4.07% | 3.15% | 1.65% | 2.64% | 2.73 % | 0.96 % | 0.39 % | 0.39 % | 0.40 % | 0.35 % | 0.16 % | 0.27 % |
| Leu | 0.33% | 0.00% | 0.12% | 0.09% | 0.22 % | 1.08 % | 0.05 % | 0.04 % | 0.04 % | 0.04 % | 0.12 % | 0.10 % |
| Lys | 5.19% | 1.17% | 1.07% | 3.73% | 13.37 % | 2.52 % | 0.15 % | 0.13 % | 0.13 % | 0.18 % | 2.78 % | 0.69 % |
| Phe | 1.72% | 0.64% | 0.55% | 2.10% | 8.25 % | 5.45 % | 0.06 % | 0.07 % | 0.09 % | 0.11 % | 3.46 % | 2.91 % |
| Thr | 2.70% | 0.50% | 0.56% | 2.97% | 0.00 % | 2.15 % | 0.31 % | 0.37 % | 0.36 % | 0.30 % | 1.49 % | 5.33 % |
| Tyr | 0.00% | 0.00% | 0.00% | 0.43% | 12.60 % | 2.51 % | 0.72 % | 0.00 % | 3.56 % | 4.64 % | 0.00 % | 0.00 % |
| Val | 10.25% | 2.67% | 3.16% | 10.62% | 28.15 % | 3.43 % | 0.12 % | 0.08 % | 0.09 % | 0.12 % | 0.81 % | 0.56 % |
