# Supplementary material for: Carbon Metabolism of Enterobacterial Human Pathogens Growing in Epithelial Colorectal Adenocarcinoma (Caco-2) Cells
Source: PLoS One. 2010 May 11;5(5):e10586. doi: 10.1371/journal.pone.0010586 (PMC2868055; doi:10.1371/journal.pone.0010586)
Supplement: Table S4 — 13C-Isotopologue abundance of amino acids from all experiments in mol%. The labelling pattern is given in terms of XY-groups (Römisch-Margl et al., 2007). This notation is based on digits for each C-atom. The first digit represents C-1, the second C-2, etc.; 1 signifies 13C, 0 signifies 12C, and X and Y signify either 13C or 12C. While the labelling status of X is totally undefined, the overall number of 13C labelled atoms of Y is known and written outside the brackets. Two independent labelling experiments were analyzed if not indicated otherwise. (1.38 MB DOC) [file pone.0010586.s006.doc]

**Table S4: ^13^C-Isotopologue abundance of amino acids from all experiments in mol%.** The labelling pattern is given in terms of XY-groups (Römisch-Margl *et al.*, 2007). This notation is based on digits for each C-atom. The first digit represents C-1, the second C-2, etc.; 1 signifies ^13^C, 0 signifies ^12^C, and X and Y signify either ^13^C or ^12^C. While the labelling status of X is totally undefined, the overall number of ^13^C labelled atoms of Y is known and written outside the brackets. Two independent labelling experiments were analyzed if not indicated otherwise.

**Reference:**

Römisch-Margl W, Schramek N, Radykewicz T, Ettenhuber C, Eylert E, Römisch-Margl L, Schwarz C, Dobner M, Demmel N, Winzenhörlein B, Bacher A, Eisenreich W (2007) ^13^CO_2_ as a universal metabolic tracer in isotopologue perturbation experiments. *Phytochemistry* 68: 2273-2289.

|  |  | *EIEC* HN280 | | | | | | | | | | | | | | | | | | | | | | | | | | | | | | | | | | |
| --- | --- | --- | --- | --- | --- | --- | --- | --- | --- | --- | --- | --- | --- | --- | --- | --- | --- | --- | --- | --- | --- | --- | --- | --- | --- | --- | --- | --- | --- | --- | --- | --- | --- | --- | --- | --- |
|  |  | wildtype | | | | | Δ*uhpT, ptsG*, *manXYZ* | | | | | | Δ*ptsG*, *manXYZ* | | | | | |  Δ*uhpT* | | | | | | wildtype | | | | | | Δ*uhpT, ptsG,* *manXYZ* | | | | | |
| Ala-260 | {000} | 64.65% | ± | | 0.21% | | 90.57% | | ± | | 0.11% | | 94.78% | | ± | | 0.13% | | 66.78% | | ± | | 0.13% | | 56.43% | | ± | | 0.06% | | 89.28% | | | ± | 0.06% | |
|  | {YYY}1 | 0.76% | ± | | 0.01% | | 0.22% | | ± | | 0.07% | | 0.24% | | ± | | 0.06% | | 0.69% | | ± | | 0.07% | | 0.64% | | ± | | 0.04% | | 0.00% | | | ± | 0.01% | |
|  | {YYY}2 | 1.61% | ± | | 0.02% | | 0.41% | | ± | | 0.06% | | 0.23% | | ± | | 0.03% | | 1.51% | | ± | | 0.02% | | 1.95% | | ± | | 0.03% | | 0.42% | | | ± | 0.02% | |
|  | {111} | 32.97% | ± | | 0.21% | | 8.80% | | ± | | 0.06% | | 4.75% | | ± | | 0.07% | | 31.02% | | ± | | 0.16% | | 40.97% | | ± | | 0.01% | | 10.29% | | | ± | 0.03% | |
| Asp-418 | {0000} | 78.58% | ± | | 0.26% | | 97.19% | | ± | | 0.11% | | 97.46% | | ± | | 0.05% | | 80.62% | | ± | | 0.45% | | 74.49% | | ± | | 0.07% | | 96.77% | | | ± | 0.07% | |
|  | {YYYY}1 | 1.14% | ± | | 0.31% | | 0.04% | | ± | | 0.06% | | 0.06% | | ± | | 0.08% | | 1.22% | | ± | | 0.30% | | 0.87% | | ± | | 0.18% | | 0.00% | | | ± | 0.00% | |
|  | {YYYY}2 | 4.77% | ± | | 0.08% | | 1.80% | | ± | | 0.08% | | 1.15% | | ± | | 0.04% | | 4.86% | | ± | | 0.08% | | 5.22% | | ± | | 0.03% | | 1.53% | | | ± | 0.08% | |
|  | {YYYY}3 | 14.46% | ± | | 0.06% | | 0.87% | | ± | | 0.07% | | 1.27% | | ± | | 0.07% | | 12.22% | | ± | | 0.16% | | 17.37% | | ± | | 0.11% | | 1.48% | | | ± | 0.02% | |
|  | {1111} | 1.06% | ± | | 0.03% | | 0.11% | | ± | | 0.02% | | 0.06% | | ± | | 0.02% | | 1.08% | | ± | | 0.05% | | 2.06% | | ± | | 0.05% | | 0.23% | | | ± | 0.01% | |
| Glu-432 | {00000} | 85.40% | ± | | 0.05% | |  | |  | |  | | 97.01% | | ± | | 0.24% | | 86.69% | | ± | | 0.10% | | 81.31% | | ± | | 0.21% | | 97.03% | | | ± | 0.26% | |
|  | {YYYYY}1 | 1.33% | ± | | 0.11% | |  | |  | |  | | 0.68% | | ± | | 0.15% | | 0.98% | | ± | | 0.09% | | 0.32% | | ± | | 0.08% | | 0.00% | | | ± | 0.00% | |
|  | {YYYYY}2 | 7.10% | ± | | 0.05% | |  | |  | |  | | 1.65% | | ± | | 0.12% | | 6.92% | | ± | | 0.06% | | 8.62% | | ± | | 0.07% | | 1.76% | | | ± | 0.27% | |
|  | {YYYYY}3 | 1.54% | ± | | 0.09% | |  | |  | |  | | 0.32% | | ± | | 0.04% | | 1.48% | | ± | | 0.04% | | 2.16% | | ± | | 0.04% | | 0.52% | | | ± | 0.04% | |
|  | {YYYYY}4 | 3.52% | ± | | 0.06% | |  | |  | |  | | 0.25% | | ± | | 0.02% | | 2.96% | | ± | | 0.03% | | 5.73% | | ± | | 0.08% | | 0.51% | | | ± | 0.02% | |
|  | {11111} | 1.10% | ± | | 0.01% | |  | |  | |  | | 0.09% | | ± | | 0.01% | | 0.98% | | ± | | 0.01% | | 1.86% | | ± | | 0.01% | | 0.18% | | | ± | 0.01% | |
| Ser-390 | {000} | 90.47% | ± | | 0.58% | | 97.44% | | ± | | 0.92% | | 97.93% | | ± | | 0.88% | | 90.24% | | ± | | 0.38% | | 79.18% | | ± | | 0.19% | | 96.81% | | | ± | 0.14% | |
|  | {YYY}1 | 3.84% | ± | | 0.38% | | 1.65% | | ± | | 0.89% | | 1.37% | | ± | | 0.85% | | 3.84% | | ± | | 0.29% | | 4.94% | | ± | | 0.13% | | 1.27% | | | ± | 0.21% | |
|  | {YYY}2 | 1.85% | ± | | 0.21% | | 0.64% | | ± | | 0.10% | | 0.46% | | ± | | 0.09% | | 1.72% | | ± | | 0.06% | | 3.26% | | ± | | 0.09% | | 0.79% | | | ± | 0.08% | |
|  | {111} | 3.84% | ± | | 0.02% | | 0.28% | | ± | | 0.05% | | 0.25% | | ± | | 0.06% | | 4.19% | | ± | | 0.06% | | 12.61% | | ± | | 0.01% | | 1.13% | | | ± | 0.01% | |
| Val-288 | {00000} | 78.02% | ± | | 0.28% | | 99.73% | | ± | | 0.05% | | 98.18% | | ± | | 0.11% | | 79.26% | | ± | | 0.13% | | 71.87% | | ± | | 0.19% | | 98.78% | | | ± | 0.07% | |
|  | {YYYYY}1 | 0.31% | ± | | 0.10% | | 0.25% | | ± | | 0.04% | | 0.43% | | ± | | 0.17% | | 0.38% | | ± | | 0.18% | | 0.17% | | ± | | 0.09% | | 0.00% | | | ± | 0.00% | |
|  | {YYYYY}2 | 3.61% | ± | | 0.04% | | 0.00% | | ± | | 0.00% | | 0.32% | | ± | | 0.06% | | 3.53% | | ± | | 0.09% | | 3.88% | | ± | | 0.02% | | 0.32% | | | ± | 0.04% | |
|  | {YYYYY}3 | 3.71% | ± | | 0.15% | | 0.01% | | ± | | 0.01% | | 0.39% | | ± | | 0.02% | | 3.59% | | ± | | 0.01% | | 4.10% | | ± | | 0.01% | | 0.26% | | | ± | 0.02% | |
|  | {YYYYY}4 | 1.02% | ± | | 0.03% | | 0.00% | | ± | | 0.00% | | 0.02% | | ± | | 0.01% | | 0.97% | | ± | | 0.07% | | 1.45% | | ± | | 0.01% | | 0.03% | | | ± | 0.02% | |
|  | {11111} | 13.33% | ± | | 0.15% | | 0.01% | | ± | | 0.02% | | 0.67% | | ± | | 0.02% | | 12.27% | | ± | | 0.10% | | 18.53% | | ± | | 0.10% | | 0.61% | | | ± | 0.03% | |
| Lys-431 | {000000} | 89.69% | ± | | 0.91% | | 99.67% | | ± | | 0.34% | | 99.49% | | ± | | 0.01% | | 89.97% | | ± | | 0.19% | | 83.12% | | ± | | 0.27% | | 96.98% | | | ± | 0.40% | |
|  | {YYYYYY}1 | 0.00% | ± | | 0.00% | | 0.12% | | ± | | 0.20% | | 0.05% | | ± | | 0.09% | | 0.01% | | ± | | 0.02% | | 0.88% | | ± | | 0.22% | | 2.56% | | | ± | 0.55% | |
|  | {YYYYYY}2 | 1.96% | ± | | 0.79% | | 0.00% | | ± | | 0.00% | | 0.00% | | ± | | 0.00% | | 2.66% | | ± | | 0.16% | | 3.14% | | ± | | 0.14% | | 0.36% | | | ± | 0.33% | |
|  | {YYYYYY}3 | 3.41% | ± | | 0.18% | | 0.19% | | ± | | 0.12% | | 0.40% | | ± | | 0.03% | | 3.08% | | ± | | 0.30% | | 3.72% | | ± | | 0.13% | | 0.05% | | | ± | 0.08% | |
|  | {YYYYYY}4 | 0.90% | ± | | 0.20% | | 0.00% | | ± | | 0.00% | | 0.00% | | ± | | 0.00% | | 0.88% | | ± | | 0.07% | | 1.61% | | ± | | 0.09% | | 0.01% | | | ± | 0.02% | |
|  | {YYYYYY}5 | 3.39% | ± | | 0.10% | | 0.00% | | ± | | 0.00% | | 0.00% | | ± | | 0.00% | | 2.75% | | ± | | 0.04% | | 6.06% | | ± | | 0.31% | | 0.03% | | | ± | 0.04% | |
|  | {111111} | 0.65% | ± | | 0.53% | | 0.02% | | ± | | 0.04% | | 0.07% | | ± | | 0.06% | | 0.65% | | ± | | 0.07% | | 1.48% | | ± | | 0.23% | | 0.01% | | | ± | 0.01% | |
|  |  | *EIEC* HN280 | | | | | | | | | | | | | | | | | | | | | | | | | | | | | | | | | |  |
|  |  | wildtype | | | | Δ*uhpT, ptsG*, *manXYZ* | | | | | | Δ*ptsG*, *manXYZ* | | | | | |  Δ*uhpT* | | | | | | wildtype | | | | | | Δ*uhpT, ptsG,* *manXYZ* | | | | | |  |
| Gly-246 | {00} | 95.44% | ± | 0.09% | | 99.03% | | ± | | 0.07% | | 99.52% | | ± | | 0.02% | | 95.46% | | ± | | 0.19% | | 86.42% | | ± | | 0.05% | | 98.89% | | ± | 0.06% | | |  |
|  | {YY}1 | 0.66% | ± | 0.08% | | 0.18% | | ± | | 0.06% | | 0.07% | | ± | | 0.01% | | 0.48% | | ± | | 0.16% | | 1.19% | | ± | | 0.01% | | 0.02% | | ± | 0.03% | | |  |
|  | {11} | 3.89% | ± | 0.01% | | 0.79% | | ± | | 0.02% | | 0.41% | | ± | | 0.01% | | 4.06% | | ± | | 0.05% | | 12.39% | | ± | | 0.05% | | 1.09% | | ± | 0.03% | | |  |
| Thr-404 | {0000} | 96.44% | ± | 0.38% | | 98.93% | | ± | | 0.20% | | 99.23% | | ± | | 0.29% | | 96.88% | | ± | | 0.36% | | 93.66% | | ± | | 0.10% | | 99.85% | | ± | 0.02% | | |  |
|  | {YYYY}1 | 0.00% | ± | 0.00% | | 0.00% | | ± | | 0.00% | | 0.00% | | ± | | 0.00% | | 0.00% | | ± | | 0.00% | | 0.64% | | ± | | 0.31% | | 0.03% | | ± | 0.02% | | |  |
|  | {YYYY}2 | 1.40% | ± | 0.31% | | 1.07% | | ± | | 0.20% | | 0.77% | | ± | | 0.29% | | 1.75% | | ± | | 0.45% | | 0.47% | | ± | | 0.36% | | 0.01% | | ± | 0.01% | | |  |
|  | {YYYY}3 | 1.70% | ± | 0.29% | | 0.00% | | ± | | 0.00% | | 0.00% | | ± | | 0.00% | | 1.12% | | ± | | 0.20% | | 4.57% | | ± | | 0.10% | | 0.08% | | ± | 0.03% | | |  |
|  | {1111} | 0.45% | ± | 0.40% | | 0.00% | | ± | | 0.00% | | 0.00% | | ± | | 0.00% | | 0.26% | | ± | | 0.10% | | 0.65% | | ± | | 0.24% | | 0.03% | | ± | 0.03% | | |  |
| Pro-286 | {00000} | 94.90% | ± | 0.60% | | 98.63% | | ± | | 0.38% | | 98.61% | | ± | | 0.51% | | 94.90% | | ± | | 0.37% | | 90.74% | | ± | | 0.96% | | 97.67% | | ± | 0.06% | | |  |
|  | {YYYYY}1 | 0.80% | ± | 0.16% | | 0.21% | | ± | | 0.36% | | 0.30% | | ± | | 0.26% | | 0.54% | | ± | | 0.07% | | 0.41% | | ± | | 0.04% | | 0.02% | | ± | 0.03% | | |  |
|  | {YYYYY}2 | 2.72% | ± | 0.50% | | 0.99% | | ± | | 0.35% | | 0.63% | | ± | | 0.35% | | 2.67% | | ± | | 0.31% | | 4.92% | | ± | | 0.04% | | 1.98% | | ± | 0.12% | | |  |
|  | {YYYYY}3 | 0.47% | ± | 0.11% | | 0.09% | | ± | | 0.16% | | 0.06% | | ± | | 0.05% | | 0.51% | | ± | | 0.10% | | 1.22% | | ± | | 0.11% | | 0.26% | | ± | 0.03% | | |  |
|  | {YYYYY}4 | 0.88% | ± | 0.01% | | 0.08% | | ± | | 0.07% | | 0.41% | | ± | | 0.40% | | 1.18% | | ± | | 0.08% | | 1.98% | | ± | | 0.91% | | 0.00% | | ± | 0.00% | | |  |
|  | {11111} | 0.23% | ± | 0.20% | | 0.00% | | ± | | 0.00% | | 0.00% | | ± | | 0.00% | | 0.20% | | ± | | 0.02% | | 0.73% | | ± | | 0.02% | | 0.07% | | ± | 0.02% | | |  |
| His-440 | {000000} | 97.27% | ± | 0.54% | | 99.42% | | ± | | 0.18% | | 98.99% | | ± | | 0.51% | | 96.67% | | ± | | 0.57% | | 94.05% | | ± | | 0.18% | | 99.63% | | ± | 0.17% | | |  |
|  | {YYYYYY}1 | 0.03% | ± | 0.05% | | 0.49% | | ± | | 0.08% | | 0.60% | | ± | | 0.50% | | 0.32% | | ± | | 0.52% | | 0.00% | | ± | | 0.00% | | 0.00% | | ± | 0.00% | | |  |
|  | {YYYYYY}2 | 0.03% | ± | 0.06% | | 0.00% | | ± | | 0.00% | | 0.03% | | ± | | 0.06% | | 0.00% | | ± | | 0.00% | | 0.00% | | ± | | 0.00% | | 0.19% | | ± | 0.09% | | |  |
|  | {YYYYYY}3 | 0.08% | ± | 0.13% | | 0.02% | | ± | | 0.02% | | 0.00% | | ± | | 0.00% | | 0.07% | | ± | | 0.06% | | 0.14% | | ± | | 0.24% | | 0.05% | | ± | 0.05% | | |  |
|  | {YYYYYY}4 | 0.13% | ± | 0.23% | | 0.02% | | ± | | 0.02% | | 0.00% | | ± | | 0.00% | | 0.09% | | ± | | 0.15% | | 0.20% | | ± | | 0.31% | | 0.00% | | ± | 0.00% | | |  |
|  | {YYYYYY}5 | 2.21% | ± | 0.37% | | 0.01% | | ± | | 0.01% | | 0.37% | | ± | | 0.15% | | 2.49% | | ± | | 0.13% | | 3.70% | | ± | | 0.11% | | 0.14% | | ± | 0.13% | | |  |
|  | {111111} | 0.25% | ± | 0.44% | | 0.05% | | ± | | 0.06% | | 0.02% | | ± | | 0.03% | | 0.37% | | ± | | 0.06% | | 1.92% | | ± | | 0.06% | | 0.00% | | ± | 0.00% | | |  |
| Phe-336 | {000000000} | 97.81% | ± | 0.38% | | 99.25% | | ± | | 0.54% | | 99.77% | | ± | | 0.26% | | 98.08% | | ± | | 0.11% | | 95.05% | | ± | | 0.18% | | 99.70% | | ± | 0.05% | | |  |
|  | {YYYYYYYYY}1 | 0.00% | ± | 0.00% | | 0.25% | | ± | | 0.27% | | 0.00% | | ± | | 0.00% | | 0.06% | | ± | | 0.11% | | 0.00% | | ± | | 0.00% | | 0.00% | | ± | 0.00% | | |  |
|  | {YYYYYYYYY}2 | 0.11% | ± | 0.13% | | 0.04% | | ± | | 0.07% | | 0.11% | | ± | | 0.18% | | 0.06% | | ± | | 0.10% | | 0.00% | | ± | | 0.00% | | 0.02% | | ± | 0.03% | | |  |
|  | {YYYYYYYYY}3 | 0.00% | ± | 0.00% | | 0.02% | | ± | | 0.03% | | 0.00% | | ± | | 0.00% | | 0.03% | | ± | | 0.03% | | 0.03% | | ± | | 0.06% | | 0.00% | | ± | 0.00% | | |  |
|  | {YYYYYYYYY}4 | 0.24% | ± | 0.19% | | 0.22% | | ± | | 0.24% | | 0.00% | | ± | | 0.00% | | 0.05% | | ± | | 0.08% | | 0.03% | | ± | | 0.06% | | 0.00% | | ± | 0.00% | | |  |
|  | {YYYYYYYYY}5 | 0.08% | ± | 0.07% | | 0.02% | | ± | | 0.04% | | 0.02% | | ± | | 0.03% | | 0.02% | | ± | | 0.03% | | 0.43% | | ± | | 0.27% | | 0.00% | | ± | 0.00% | | |  |
|  | {YYYYYYYYY}6 | 0.21% | ± | 0.17% | | 0.06% | | ± | | 0.07% | | 0.01% | | ± | | 0.01% | | 0.25% | | ± | | 0.09% | | 0.38% | | ± | | 0.16% | | 0.00% | | ± | 0.00% | | |  |
|  | {YYYYYYYYY}7 | 0.17% | ± | 0.03% | | 0.01% | | ± | | 0.02% | | 0.00% | | ± | | 0.01% | | 0.11% | | ± | | 0.03% | | 0.20% | | ± | | 0.16% | | 0.06% | | ± | 0.06% | | |  |
|  | {YYYYYYYYY}8 | 0.07% | ± | 0.06% | | 0.10% | | ± | | 0.17% | | 0.05% | | ± | | 0.06% | | 0.10% | | ± | | 0.05% | | 0.45% | | ± | | 0.22% | | 0.12% | | ± | 0.10% | | |  |
|  | {111111111} | 1.33% | ± | 0.09% | | 0.03% | | ± | | 0.04% | | 0.05% | | ± | | 0.05% | | 1.25% | | ± | | 0.03% | | 3.43% | | ± | | 0.16% | | 0.11% | | ± | 0.08% | | |  |

|  |  | *EIEC* HN280 | | | | | | | | | | | | | | | | | |
| --- | --- | --- | --- | --- | --- | --- | --- | --- | --- | --- | --- | --- | --- | --- | --- | --- | --- | --- | --- |
|  |  | wildtype | | | Δ*uhpT*, *ptsG*, *manXYZ* | | | Δ*ptsG*, *manXYZ* | | |  Δ*uhpT* | | | wildtype | | | Δ*uhpT*, *ptsG*, *manXYZ* | | |
| Tyr-466 | {000000000} | 95.08% | ± | 0.24% | 78.40% | ± | 10.61% | 100.00% | ± | 0.00% | 95.42% | ± | 0.12% | 92.33% | ± | 0.19% | 99.82% | ± | 0.04% |
|  | {YYYYYYYYY}1 | 0.00% | ± | 0.00% | 0.00% | ± | 0.00% | 0.00% | ± | 0.00% | 0.05% | ± | 0.09% | 0.00% | ± | 0.00% | 0.00% | ± | 0.00% |
|  | {YYYYYYYYY}2 | 0.00% | ± | 0.00% | 0.00% | ± | 0.00% | 0.00% | ± | 0.00% | 0.00% | ± | 0.00% | 0.04% | ± | 0.06% | 0.00% | ± | 0.00% |
|  | {YYYYYYYYY}3 | 0.02% | ± | 0.04% | 4.78% | ± | 4.25% | 0.00% | ± | 0.00% | 0.00% | ± | 0.00% | 0.07% | ± | 0.07% | 0.06% | ± | 0.06% |
|  | {YYYYYYYYY}4 | 0.00% | ± | 0.00% | 4.54% | ± | 7.64% | 0.00% | ± | 0.00% | 0.00% | ± | 0.00% | 0.02% | ± | 0.03% | 0.02% | ± | 0.03% |
|  | {YYYYYYYYY}5 | 0.10% | ± | 0.02% | 4.70% | ± | 2.52% | 0.00% | ± | 0.00% | 0.00% | ± | 0.00% | 0.20% | ± | 0.09% | 0.01% | ± | 0.02% |
|  | {YYYYYYYYY}6 | 0.09% | ± | 0.15% | 0.00% | ± | 0.00% | 0.00% | ± | 0.00% | 0.09% | ± | 0.12% | 0.00% | ± | 0.00% | 0.00% | ± | 0.00% |
|  | {YYYYYYYYY}7 | 0.32% | ± | 0.23% | 0.00% | ± | 0.00% | 0.00% | ± | 0.00% | 0.26% | ± | 0.06% | 0.06% | ± | 0.04% | 0.00% | ± | 0.00% |
|  | {YYYYYYYYY}8 | 0.69% | ± | 0.11% | 2.70% | ± | 4.68% | 0.00% | ± | 0.00% | 0.45% | ± | 0.08% | 0.74% | ± | 0.29% | 0.00% | ± | 0.00% |
|  | {111111111} | 3.69% | ± | 0.20% | 4.88% | ± | 5.32% | 0.00% | ± | 0.00% | 3.73% | ± | 0.07% | 6.55% | ± | 0.06% | 0.08% | ± | 0.03% |
| Ile-200 | {X00000} | 96.90% | ± | 0.10% | 98.79% | ± | 0.72% | 98.33% | ± | 0.13% | 98.29% | ± | 0.67% | 96.90% | ± | 0.06% | 99.64% | ± | 0.10% |
|  | {XYYYYY}1 | 0.24% | ± | 0.03% | 0.11% | ± | 0.05% | 0.13% | ± | 0.05% | 0.15% | ± | 0.03% | 0.00% | ± | 0.00% | 0.00% | ± | 0.00% |
|  | {XYYYYY}2 | 0.16% | ± | 0.06% | 0.00% | ± | 0.00% | 0.00% | ± | 0.00% | 0.07% | ± | 0.05% | 1.39% | ± | 0.02% | 0.13% | ± | 0.02% |
|  | {XYYYYY}3 | 0.17% | ± | 0.04% | 0.00% | ± | 0.00% | 0.00% | ± | 0.01% | 0.12% | ± | 0.02% | 0.37% | ± | 0.01% | 0.15% | ± | 0.01% |
|  | {XYYYYY}4 | 0.15% | ± | 0.00% | 0.00% | ± | 0.00% | 0.00% | ± | 0.00% | 0.12% | ± | 0.01% | 0.61% | ± | 0.02% | 0.02% | ± | 0.01% |
|  | {X11111} | 2.38% | ± | 0.09% | 1.10% | ± | 0.75% | 1.54% | ± | 0.11% | 1.23% | ± | 0.66% | 0.73% | ± | 0.05% | 0.07% | ± | 0.08% |
| Leu-274 | {X00000} | 99.51% | ± | 0.08% | 99.76% | ± | 0.20% | 99.70% | ± | 0.09% | 99.63% | ± | 0.18% | 99.65% | ± | 0.01% | 99.96% | ± | 0.00% |
|  | {XYYYYY}1 | 0.31% | ± | 0.08% | 0.21% | ± | 0.21% | 0.28% | ± | 0.07% | 0.23% | ± | 0.17% | 0.00% | ± | 0.00% | 0.00% | ± | 0.00% |
|  | {XYYYYY}2 | 0.07% | ± | 0.00% | 0.02% | ± | 0.03% | 0.00% | ± | 0.01% | 0.05% | ± | 0.04% | 0.00% | ± | 0.00% | 0.00% | ± | 0.00% |
|  | {XYYYYY}3 | 0.02% | ± | 0.01% | 0.01% | ± | 0.02% | 0.00% | ± | 0.00% | 0.02% | ± | 0.03% | 0.05% | ± | 0.01% | 0.00% | ± | 0.00% |
|  | {XYYYYY}4 | 0.03% | ± | 0.01% | 0.01% | ± | 0.02% | 0.01% | ± | 0.01% | 0.03% | ± | 0.01% | 0.04% | ± | 0.02% | 0.00% | ± | 0.00% |
|  | {X11111} | 0.07% | ± | 0.02% | 0.00% | ± | 0.00% | 0.00% | ± | 0.00% | 0.04% | ± | 0.01% | 0.25% | ± | 0.02% | 0.03% | ± | 0.00% |

|  |  | *Caco-2 cells infected with EIEC* HN280 | | | | | | | | | | | | | | | | | | | | | | | | | | | | | | | | | | |
| --- | --- | --- | --- | --- | --- | --- | --- | --- | --- | --- | --- | --- | --- | --- | --- | --- | --- | --- | --- | --- | --- | --- | --- | --- | --- | --- | --- | --- | --- | --- | --- | --- | --- | --- | --- | --- |
|  |  | wildtype | | | | | Δ*uhpT*, *ptsG*, *manXYZ* | | | | | | Δ*ptsG*, *manXYZ* | | | | | |  Δ*uhpT* | | | | | | wildtype | | | | | | Δ*uhpT*, *ptsG*, *manXYZ* | | | | | |
| Ala-260 | {000} | 93.81% | ± | | 0.04% | | 92.21% | | ± | | 0.17% | | 92.37% | | ± | | 0.09% | | 92.79% | | ± | | 0.08% | | 84.85% | | ± | | 0.07% | | 83.18% | | | ± | 0.05% | |
|  | {YYY}1 | 0.26% | ± | | 0.03% | | 0.24% | | ± | | 0.07% | | 0.33% | | ± | | 0.10% | | 0.36% | | ± | | 0.02% | | 0.10% | | ± | | 0.03% | | 0.06% | | | ± | 0.01% | |
|  | {YYY}2 | 0.30% | ± | | 0.01% | | 0.40% | | ± | | 0.04% | | 0.35% | | ± | | 0.03% | | 0.38% | | ± | | 0.03% | | 0.68% | | ± | | 0.01% | | 0.69% | | | ± | 0.02% | |
|  | {111} | 5.63% | ± | | 0.04% | | 7.15% | | ± | | 0.12% | | 6.95% | | ± | | 0.10% | | 6.47% | | ± | | 0.08% | | 14.36% | | ± | | 0.05% | | 16.07% | | | ± | 0.03% | |
| Asp-418 | {0000} | 97.83% | ± | | 0.11% | | 96.25% | | ± | | 0.19% | | 97.21% | | ± | | 0.07% | | 97.08% | | ± | | 0.32% | | 95.94% | | ± | | 0.10% | | 96.67% | | | ± | 0.13% | |
|  | {YYYY}1 | 0.06% | ± | | 0.11% | | 0.20% | | ± | | 0.18% | | 0.14% | | ± | | 0.13% | | 0.33% | | ± | | 0.42% | | 0.08% | | ± | | 0.11% | | 0.08% | | | ± | 0.12% | |
|  | {YYYY}2 | 1.42% | ± | | 0.08% | | 1.61% | | ± | | 0.07% | | 1.77% | | ± | | 0.05% | | 1.72% | | ± | | 0.10% | | 2.25% | | ± | | 0.01% | | 1.81% | | | ± | 0.00% | |
|  | {YYYY}3 | 0.62% | ± | | 0.07% | | 1.78% | | ± | | 0.22% | | 0.80% | | ± | | 0.06% | | 0.77% | | ± | | 0.09% | | 1.40% | | ± | | 0.03% | | 1.17% | | | ± | 0.01% | |
|  | {1111} | 0.07% | ± | | 0.01% | | 0.16% | | ± | | 0.05% | | 0.08% | | ± | | 0.02% | | 0.09% | | ± | | 0.03% | | 0.33% | | ± | | 0.01% | | 0.26% | | | ± | 0.00% | |
| Glu-432 | {00000} | 92.23% | ± | | 0.27% | | 96.80% | | ± | | 0.00% | | 89.83% | | ± | | 0.11% | | 89.79% | | ± | | 0.16% | | 87.73% | | ± | | 0.16% | | 89.52% | | | ± | 0.10% | |
|  | {YYYYY}1 | 1.12% | ± | | 0.30% | | 0.71% | | ± | | 0.20% | | 1.55% | | ± | | 0.21% | | 1.71% | | ± | | 0.16% | | 0.19% | | ± | | 0.12% | | 0.00% | | | ± | 0.00% | |
|  | {YYYYY}2 | 4.69% | ± | | 0.07% | | 1.71% | | ± | | 0.20% | | 6.08% | | ± | | 0.11% | | 5.88% | | ± | | 0.08% | | 7.26% | | ± | | 0.10% | | 6.52% | | | ± | 0.11% | |
|  | {YYYYY}3 | 0.96% | ± | | 0.05% | | 0.34% | | ± | | 0.10% | | 1.28% | | ± | | 0.04% | | 1.26% | | ± | | 0.04% | | 1.93% | | ± | | 0.04% | | 1.65% | | | ± | 0.02% | |
|  | {YYYYY}4 | 0.78% | ± | | 0.01% | | 0.34% | | ± | | 0.03% | | 0.99% | | ± | | 0.03% | | 1.05% | | ± | | 0.01% | | 2.07% | | ± | | 0.01% | | 1.63% | | | ± | 0.00% | |
|  | {11111} | 0.21% | ± | | 0.01% | | 0.11% | | ± | | 0.04% | | 0.28% | | ± | | 0.00% | | 0.30% | | ± | | 0.00% | | 0.81% | | ± | | 0.01% | | 0.68% | | | ± | 0.00% | |
| Ser-390 | {000} | 97.56% | ± | | 0.55% | | 97.77% | | ± | | 1.55% | | 97.65% | | ± | | 0.90% | | 97.64% | | ± | | 0.84% | | 95.85% | | ± | | 0.43% | | 95.35% | | | ± | 0.06% | |
|  | {YYY}1 | 1.64% | ± | | 0.51% | | 1.46% | | ± | | 1.26% | | 1.46% | | ± | | 0.80% | | 1.43% | | ± | | 0.76% | | 1.55% | | ± | | 0.44% | | 2.22% | | | ± | 0.01% | |
|  | {YYY}2 | 0.58% | ± | | 0.08% | | 0.45% | | ± | | 0.39% | | 0.59% | | ± | | 0.17% | | 0.63% | | ± | | 0.14% | | 0.87% | | ± | | 0.02% | | 1.12% | | | ± | 0.07% | |
|  | {111} | 0.21% | ± | | 0.03% | | 0.33% | | ± | | 0.10% | | 0.29% | | ± | | 0.06% | | 0.30% | | ± | | 0.06% | | 1.72% | | ± | | 0.01% | | 1.30% | | | ± | 0.02% | |
| Val-288 | {00000} | 99.71% | ± | | 0.15% | | 97.40% | | ± | | 0.12% | | 99.58% | | ± | | 0.08% | | 99.65% | | ± | | 0.08% | | 99.81% | | ± | | 0.01% | | 99.99% | | | ± | 0.01% | |
|  | {YYYYY}1 | 0.24% | ± | | 0.17% | | 0.37% | | ± | | 0.11% | | 0.41% | | ± | | 0.07% | | 0.26% | | ± | | 0.07% | | 0.00% | | ± | | 0.00% | | 0.00% | | | ± | 0.00% | |
|  | {YYYYY}2 | 0.00% | ± | | 0.00% | | 0.64% | | ± | | 0.05% | | 0.00% | | ± | | 0.00% | | 0.00% | | ± | | 0.00% | | 0.00% | | ± | | 0.01% | | 0.00% | | | ± | 0.00% | |
|  | {YYYYY}3 | 0.01% | ± | | 0.02% | | 0.75% | | ± | | 0.08% | | 0.01% | | ± | | 0.01% | | 0.03% | | ± | | 0.02% | | 0.01% | | ± | | 0.01% | | 0.00% | | | ± | 0.00% | |
|  | {YYYYY}4 | 0.00% | ± | | 0.00% | | 0.03% | | ± | | 0.03% | | 0.00% | | ± | | 0.00% | | 0.00% | | ± | | 0.00% | | 0.02% | | ± | | 0.00% | | 0.00% | | | ± | 0.00% | |
|  | {11111} | 0.03% | ± | | 0.00% | | 0.81% | | ± | | 0.03% | | 0.00% | | ± | | 0.00% | | 0.07% | | ± | | 0.01% | | 0.15% | | ± | | 0.00% | | 0.01% | | | ± | 0.01% | |
| Lys-431 | {000000} | 99.81% | ± | | 0.04% | | 99.40% | | ± | | 0.30% | | 99.76% | | ± | | 0.07% | | 99.73% | | ± | | 0.08% | | 99.85% | | ± | | 0.09% | | 99.82% | | | ± | 0.15% | |
|  | {YYYYYY}1 | 0.03% | ± | | 0.05% | | 0.12% | | ± | | 0.20% | | 0.00% | | ± | | 0.00% | | 0.03% | | ± | | 0.05% | | 0.08% | | ± | | 0.13% | | 0.15% | | | ± | 0.15% | |
|  | {YYYYYY}2 | 0.00% | ± | | 0.00% | | 0.00% | | ± | | 0.00% | | 0.00% | | ± | | 0.00% | | 0.00% | | ± | | 0.00% | | 0.05% | | ± | | 0.09% | | 0.00% | | | ± | 0.00% | |
|  | {YYYYYY}3 | 0.16% | ± | | 0.09% | | 0.38% | | ± | | 0.24% | | 0.23% | | ± | | 0.06% | | 0.23% | | ± | | 0.04% | | 0.00% | | ± | | 0.01% | | 0.00% | | | ± | 0.00% | |
|  | {YYYYYY}4 | 0.00% | ± | | 0.00% | | 0.00% | | ± | | 0.00% | | 0.00% | | ± | | 0.00% | | 0.00% | | ± | | 0.00% | | 0.02% | | ± | | 0.03% | | 0.03% | | | ± | 0.00% | |
|  | {YYYYYY}5 | 0.00% | ± | | 0.00% | | 0.02% | | ± | | 0.03% | | 0.00% | | ± | | 0.00% | | 0.00% | | ± | | 0.00% | | 0.00% | | ± | | 0.00% | | 0.00% | | | ± | 0.00% | |
|  | {111111} | 0.00% | ± | | 0.00% | | 0.08% | | ± | | 0.05% | | 0.01% | | ± | | 0.02% | | 0.01% | | ± | | 0.02% | | 0.00% | | ± | | 0.00% | | 0.00% | | | ± | 0.00% | |
|  |  | *Caco-2 cells infected with EIEC* HN280 | | | | | | | | | | | | | | | | | | | | | | | | | | | | | | | | | |  |
|  |  | wildtype | | | | Δ*uhpT*, *ptsG*, *manXYZ* | | | | | | Δ*ptsG*, *manXYZ* | | | | | |  Δ*uhpT* | | | | | | wildtype | | | | | | Δ*uhpT*, *ptsG*, *manXYZ* | | | | | |  |
| Gly-246 | {00} | 99.40% | ± | 0.10% | | 99.37% | | ± | | 0.12% | | 99.25% | | ± | | 0.01% | | 99.20% | | ± | | 0.09% | | 97.37% | | ± | | 0.05% | | 97.74% | | ± | 0.02% | | |  |
|  | {YY}1 | 0.10% | ± | 0.08% | | 0.19% | | ± | | 0.11% | | 0.13% | | ± | | 0.01% | | 0.13% | | ± | | 0.09% | | 0.00% | | ± | | 0.00% | | 0.01% | | ± | 0.02% | | |  |
|  | {11} | 0.49% | ± | 0.03% | | 0.44% | | ± | | 0.01% | | 0.63% | | ± | | 0.02% | | 0.67% | | ± | | 0.00% | | 2.63% | | ± | | 0.05% | | 2.25% | | ± | 0.02% | | |  |
| Thr-404 | {0000} | 99.06% | ± | 0.38% | | 99.13% | | ± | | 0.52% | | 99.04% | | ± | | 0.63% | | 98.95% | | ± | | 0.18% | | 99.76% | | ± | | 0.08% | | 99.87% | | ± | 0.04% | | |  |
|  | {YYYY}1 | 0.00% | ± | 0.00% | | 0.00% | | ± | | 0.00% | | 0.01% | | ± | | 0.02% | | 0.00% | | ± | | 0.00% | | 0.17% | | ± | | 0.15% | | 0.00% | | ± | 0.00% | | |  |
|  | {YYYY}2 | 0.94% | ± | 0.38% | | 0.85% | | ± | | 0.55% | | 0.95% | | ± | | 0.64% | | 1.05% | | ± | | 0.18% | | 0.05% | | ± | | 0.09% | | 0.13% | | ± | 0.04% | | |  |
|  | {YYYY}3 | 0.00% | ± | 0.00% | | 0.00% | | ± | | 0.00% | | 0.00% | | ± | | 0.00% | | 0.00% | | ± | | 0.00% | | 0.00% | | ± | | 0.00% | | 0.00% | | ± | 0.00% | | |  |
|  | {1111} | 0.00% | ± | 0.00% | | 0.02% | | ± | | 0.03% | | 0.00% | | ± | | 0.00% | | 0.00% | | ± | | 0.00% | | 0.02% | | ± | | 0.02% | | 0.00% | | ± | 0.00% | | |  |
| Pro-286 | {00000} | 98.32% | ± | 0.26% | | 99.23% | | ± | | 0.59% | | 97.95% | | ± | | 0.51% | | 98.70% | | ± | | 0.52% | | 96.06% | | ± | | 0.18% | | 95.31% | | ± | 0.15% | | |  |
|  | {YYYYY}1 | 0.39% | ± | 0.07% | | 0.06% | | ± | | 0.10% | | 0.30% | | ± | | 0.32% | | 0.15% | | ± | | 0.18% | | 0.08% | | ± | | 0.09% | | 0.05% | | ± | 0.02% | | |  |
|  | {YYYYY}2 | 0.95% | ± | 0.28% | | 0.68% | | ± | | 0.55% | | 1.06% | | ± | | 0.43% | | 0.96% | | ± | | 0.35% | | 3.08% | | ± | | 0.10% | | 3.36% | | ± | 0.12% | | |  |
|  | {YYYYY}3 | 0.12% | ± | 0.04% | | 0.01% | | ± | | 0.02% | | 0.12% | | ± | | 0.02% | | 0.10% | | ± | | 0.08% | | 0.63% | | ± | | 0.02% | | 0.57% | | ± | 0.01% | | |  |
|  | {YYYYY}4 | 0.20% | ± | 0.01% | | 0.02% | | ± | | 0.02% | | 0.56% | | ± | | 0.21% | | 0.09% | | ± | | 0.10% | | 0.00% | | ± | | 0.00% | | 0.52% | | ± | 0.04% | | |  |
|  | {11111} | 0.00% | ± | 0.00% | | 0.00% | | ± | | 0.00% | | 0.00% | | ± | | 0.00% | | 0.00% | | ± | | 0.00% | | 0.15% | | ± | | 0.03% | | 0.20% | | ± | 0.01% | | |  |
| His-440 | {000000} | 99.36% | ± | 0.24% | | 99.95% | |  | |  | | 99.55% | | ± | | 0.28% | | 99.50% | | ± | | 0.45% | | 97.92% | | ± | | 0.20% | | 98.27% | | ± | 0.17% | | |  |
|  | {YYYYYY}1 | 0.53% | ± | 0.17% | | 0.00% | |  | |  | | 0.40% | | ± | | 0.29% | | 0.22% | | ± | | 0.24% | | 0.00% | | ± | | 0.00% | | 0.00% | | ± | 0.00% | | |  |
|  | {YYYYYY}2 | 0.00% | ± | 0.00% | | 0.05% | |  | |  | | 0.00% | | ± | | 0.00% | | 0.02% | | ± | | 0.03% | | 1.23% | | ± | | 0.11% | | 1.27% | | ± | 0.24% | | |  |
|  | {YYYYYY}3 | 0.00% | ± | 0.00% | | 0.00% | |  | |  | | 0.01% | | ± | | 0.02% | | 0.09% | | ± | | 0.16% | | 0.29% | | ± | | 0.39% | | 0.39% | | ± | 0.04% | | |  |
|  | {YYYYYY}4 | 0.03% | ± | 0.05% | | 0.00% | |  | |  | | 0.00% | | ± | | 0.00% | | 0.17% | | ± | | 0.26% | | 0.20% | | ± | | 0.18% | | 0.00% | | ± | 0.00% | | |  |
|  | {YYYYYY}5 | 0.05% | ± | 0.05% | | 0.00% | |  | |  | | 0.03% | | ± | | 0.02% | | 0.00% | | ± | | 0.00% | | 0.17% | | ± | | 0.16% | | 0.00% | | ± | 0.00% | | |  |
|  | {111111} | 0.03% | ± | 0.04% | | 0.00% | |  | |  | | 0.01% | | ± | | 0.01% | | 0.00% | | ± | | 0.00% | | 0.19% | | ± | | 0.21% | | 0.07% | | ± | 0.05% | | |  |
| Phe-336 | {000000000} | 99.78% | ± | 0.13% | | 98.77% | | ± | | 0.57% | | 99.45% | | ± | | 0.51% | | 99.48% | | ± | | 0.33% | |  | |  | |  | |  | |  |  | | |  |
|  | {YYYYYYYYY}1 | 0.00% | ± | 0.00% | | 0.00% | | ± | | 0.00% | | 0.36% | | ± | | 0.62% | | 0.00% | | ± | | 0.00% | |  | |  | |  | |  | |  |  | | |  |
|  | {YYYYYYYYY}2 | 0.15% | ± | 0.13% | | 0.23% | | ± | | 0.17% | | 0.10% | | ± | | 0.09% | | 0.14% | | ± | | 0.13% | |  | |  | |  | |  | |  |  | | |  |
|  | {YYYYYYYYY}3 | 0.00% | ± | 0.00% | | 0.07% | | ± | | 0.12% | | 0.01% | | ± | | 0.02% | | 0.02% | | ± | | 0.03% | |  | |  | |  | |  | |  |  | | |  |
|  | {YYYYYYYYY}4 | 0.02% | ± | 0.04% | | 0.38% | | ± | | 0.30% | | 0.03% | | ± | | 0.05% | | 0.26% | | ± | | 0.16% | |  | |  | |  | |  | |  |  | | |  |
|  | {YYYYYYYYY}5 | 0.00% | ± | 0.00% | | 0.03% | | ± | | 0.05% | | 0.00% | | ± | | 0.00% | | 0.00% | | ± | | 0.00% | |  | |  | |  | |  | |  |  | | |  |
|  | {YYYYYYYYY}6 | 0.00% | ± | 0.01% | | 0.10% | | ± | | 0.09% | | 0.03% | | ± | | 0.03% | | 0.08% | | ± | | 0.06% | |  | |  | |  | |  | |  |  | | |  |
|  | {YYYYYYYYY}7 | 0.02% | ± | 0.02% | | 0.14% | | ± | | 0.15% | | 0.01% | | ± | | 0.01% | | 0.01% | | ± | | 0.02% | |  | |  | |  | |  | |  |  | | |  |
|  | {YYYYYYYYY}8 | 0.02% | ± | 0.03% | | 0.00% | | ± | | 0.00% | | 0.00% | | ± | | 0.00% | | 0.00% | | ± | | 0.00% | |  | |  | |  | |  | |  |  | | |  |
|  | {111111111} | 0.00% | ± | 0.00% | | 0.28% | | ± | | 0.24% | | 0.01% | | ± | | 0.01% | | 0.02% | | ± | | 0.03% | |  | |  | |  | |  | |  |  | | |  |

|  |  | *Caco-2 cells infected with EIEC* HN280 | | | | | | | | | | | | | | | | | |
| --- | --- | --- | --- | --- | --- | --- | --- | --- | --- | --- | --- | --- | --- | --- | --- | --- | --- | --- | --- |
|  |  | wildtype | | | Δ*uhpT*, *ptsG*, *manXYZ* | | | Δ*ptsG*, *manXYZ* | | |  Δ*uhpT* | | | wildtype | | | Δ*uhpT*, *ptsG*, *manXYZ* | | |
| Tyr-466 | {000000000} | 97.29% | ± | 2.09% | 93.96% | ± | 11.30% | 97.33% | ± | 2.35% | 97.29% | ± | 2.96% |  |  |  |  |  |  |
|  | {YYYYYYYYY}1 | 0.32% | ± | 0.55% | 0.00% | ± | 0.00% | 0.00% | ± | 0.00% | 1.04% | ± | 1.14% |  |  |  |  |  |  |
|  | {YYYYYYYYY}2 | 0.00% | ± | 0.00% | 0.10% | ± | 0.19% | 0.00% | ± | 0.00% | 0.00% | ± | 0.00% |  |  |  |  |  |  |
|  | {YYYYYYYYY}3 | 0.00% | ± | 0.00% | 2.15% | ± | 3.98% | 0.00% | ± | 0.00% | 0.00% | ± | 0.00% |  |  |  |  |  |  |
|  | {YYYYYYYYY}4 | 0.95% | ± | 1.65% | 0.00% | ± | 0.00% | 0.00% | ± | 0.00% | 0.00% | ± | 0.00% |  |  |  |  |  |  |
|  | {YYYYYYYYY}5 | 0.00% | ± | 0.00% | 0.67% | ± | 1.35% | 0.00% | ± | 0.00% | 0.00% | ± | 0.00% |  |  |  |  |  |  |
|  | {YYYYYYYYY}6 | 0.00% | ± | 0.00% | 0.00% | ± | 0.00% | 0.39% | ± | 0.68% | 0.00% | ± | 0.00% |  |  |  |  |  |  |
|  | {YYYYYYYYY}7 | 0.72% | ± | 1.26% | 0.00% | ± | 0.00% | 2.28% | ± | 2.22% | 0.00% | ± | 0.00% |  |  |  |  |  |  |
|  | {YYYYYYYYY}8 | 0.09% | ± | 0.15% | 2.03% | ± | 4.06% | 0.00% | ± | 0.00% | 0.00% | ± | 0.00% |  |  |  |  |  |  |
|  | {111111111} | 0.63% | ± | 1.09% | 1.08% | ± | 1.99% | 0.00% | ± | 0.00% | 1.67% | ± | 2.89% |  |  |  |  |  |  |
| Ile-200 | {X00000} | 99.37% | ± | 0.40% | 97.54% | ± | 2.09% | 99.41% | ± | 0.10% | 99.40% | ± | 0.07% | 99.67% | ± | 0.02% | 99.85% | ± | 0.02% |
|  | {XYYYYY}1 | 0.13% | ± | 0.03% | 0.13% | ± | 0.03% | 0.14% | ± | 0.04% | 0.11% | ± | 0.06% | 0.00% | ± | 0.00% | 0.00% | ± | 0.00% |
|  | {XYYYYY}2 | 0.00% | ± | 0.00% | 0.00% | ± | 0.00% | 0.00% | ± | 0.00% | 0.00% | ± | 0.00% | 0.10% | ± | 0.01% | 0.05% | ± | 0.02% |
|  | {XYYYYY}3 | 0.00% | ± | 0.00% | 0.04% | ± | 0.03% | 0.00% | ± | 0.00% | 0.00% | ± | 0.00% | 0.08% | ± | 0.00% | 0.03% | ± | 0.01% |
|  | {XYYYYY}4 | 0.00% | ± | 0.00% | 0.00% | ± | 0.00% | 0.00% | ± | 0.00% | 0.00% | ± | 0.00% | 0.02% | ± | 0.01% | 0.01% | ± | 0.00% |
|  | {X11111} | 0.49% | ± | 0.43% | 2.29% | ± | 2.09% | 0.46% | ± | 0.06% | 0.48% | ± | 0.10% | 0.14% | ± | 0.01% | 0.05% | ± | 0.01% |
| Leu-274 | {X00000} | 99.60% | ± | 0.08% | 99.61% | ± | 0.04% | 99.53% | ± | 0.27% | 99.53% | ± | 0.07% | 99.96% | ± | 0.01% | 99.97% | ± | 0.00% |
|  | {XYYYYY}1 | 0.35% | ± | 0.05% | 0.32% | ± | 0.04% | 0.42% | ± | 0.24% | 0.43% | ± | 0.07% | 0.00% | ± | 0.00% | 0.00% | ± | 0.00% |
|  | {XYYYYY}2 | 0.03% | ± | 0.04% | 0.03% | ± | 0.03% | 0.04% | ± | 0.03% | 0.03% | ± | 0.05% | 0.00% | ± | 0.00% | 0.00% | ± | 0.00% |
|  | {XYYYYY}3 | 0.01% | ± | 0.01% | 0.01% | ± | 0.01% | 0.00% | ± | 0.00% | 0.00% | ± | 0.00% | 0.00% | ± | 0.01% | 0.00% | ± | 0.00% |
|  | {XYYYYY}4 | 0.01% | ± | 0.01% | 0.02% | ± | 0.02% | 0.01% | ± | 0.01% | 0.01% | ± | 0.01% | 0.01% | ± | 0.00% | 0.01% | ± | 0.00% |
|  | {X11111} | 0.00% | ± | 0.00% | 0.00% | ± | 0.01% | 0.00% | ± | 0.00% | 0.00% | ± | 0.00% | 0.02% | ± | 0.00% | 0.02% | ± | 0.00% |

|  |  | *EIEC* 4608-58 | | | | | | | | | | | *Caco-2 cells infected with EIEC* 4608-58 | | | | | | | | | | | |
| --- | --- | --- | --- | --- | --- | --- | --- | --- | --- | --- | --- | --- | --- | --- | --- | --- | --- | --- | --- | --- | --- | --- | --- | --- |
|  |  | wildtype | | | | | Δ*uhpT, ptsG, manXYZ* | | | | | | wildtype | | | | | | Δ*uhpT, ptsG, manXYZ* | | | | | |
| Ala-260 | {000} | 87.40% | ± | | 0.08% | | 93.12% | | ± | | 0.08% | | 86.19% | | ± | | 0.02% | | 86.55% | | ± | | 0.08% | |
|  | {YYY}1 | 0.05% | ± | | 0.02% | | 0.03% | | ± | | 0.05% | | 0.05% | | ± | | 0.02% | | 0.05% | | ± | | 0.05% | |
|  | {YYY}2 | 0.54% | ± | | 0.01% | | 0.25% | | ± | | 0.01% | | 0.58% | | ± | | 0.03% | | 0.55% | | ± | | 0.03% | |
|  | {111} | 12.01% | ± | | 0.06% | | 6.60% | | ± | | 0.03% | | 13.18% | | ± | | 0.02% | | 12.86% | | ± | | 0.06% | |
| Asp-418 | {0000} | 90.80% | ± | | 0.23% | | 97.78% | | ± | | 0.15% | | 96.18% | | ± | | 0.21% | | 96.44% | | ± | | 0.06% | |
|  | {YYYY}1 | 0.25% | ± | | 0.21% | | 0.00% | | ± | | 0.00% | | 0.29% | | ± | | 0.16% | | 0.19% | | ± | | 0.12% | |
|  | {YYYY}2 | 2.80% | ± | | 0.07% | | 1.36% | | ± | | 0.21% | | 2.23% | | ± | | 0.08% | | 2.03% | | ± | | 0.09% | |
|  | {YYYY}3 | 5.53% | ± | | 0.03% | | 0.72% | | ± | | 0.05% | | 1.06% | | ± | | 0.03% | | 1.11% | | ± | | 0.02% | |
|  | {1111} | 0.62% | ± | | 0.02% | | 0.14% | | ± | | 0.04% | | 0.23% | | ± | | 0.02% | | 0.24% | | ± | | 0.01% | |
| Glu-432 | {00000} | 94.00% | ± | | 0.21% | | 97.73% | | ± | | 0.10% | | 91.48% | | ± | | 0.11% | | 92.32% | | ± | | 0.16% | |
|  | {YYYYY}1 | 0.19% | ± | | 0.03% | | 0.00% | | ± | | 0.00% | | 0.54% | | ± | | 0.09% | | 0.45% | | ± | | 0.15% | |
|  | {YYYYY}2 | 3.06% | ± | | 0.15% | | 1.43% | | ± | | 0.05% | | 4.88% | | ± | | 0.05% | | 4.36% | | ± | | 0.09% | |
|  | {YYYYY}3 | 0.81% | ± | | 0.03% | | 0.38% | | ± | | 0.06% | | 1.25% | | ± | | 0.03% | | 1.13% | | ± | | 0.03% | |
|  | {YYYYY}4 | 1.42% | ± | | 0.01% | | 0.34% | | ± | | 0.01% | | 1.32% | | ± | | 0.02% | | 1.23% | | ± | | 0.01% | |
|  | {11111} | 0.52% | ± | | 0.01% | | 0.12% | | ± | | 0.00% | | 0.52% | | ± | | 0.01% | | 0.52% | | ± | | 0.00% | |
| Ser-390 | {000} | 97.04% | ± | | 0.36% | | 98.71% | | ± | | 0.05% | | 97.36% | | ± | | 0.54% | | 97.89% | | ± | | 0.26% | |
|  | {YYY}1 | 0.35% | ± | | 0.27% | | 0.00% | | ± | | 0.00% | | 0.30% | | ± | | 0.48% | | 0.10% | | ± | | 0.14% | |
|  | {YYY}2 | 0.67% | ± | | 0.12% | | 0.38% | | ± | | 0.09% | | 0.81% | | ± | | 0.08% | | 0.68% | | ± | | 0.18% | |
|  | {111} | 1.94% | ± | | 0.02% | | 0.91% | | ± | | 0.04% | | 1.52% | | ± | | 0.03% | | 1.33% | | ± | | 0.04% | |
| Val-288 | {00000} | 96.59% | ± | | 0.06% | | 99.87% | | ± | | 0.08% | | 99.97% | | ± | | 0.01% | | 99.89% | | ± | | 0.06% | |
|  | {YYYYY}1 | 0.00% | ± | | 0.00% | | 0.00% | | ± | | 0.00% | | 0.00% | | ± | | 0.00% | | 0.00% | | ± | | 0.00% | |
|  | {YYYYY}2 | 0.53% | ± | | 0.02% | | 0.05% | | ± | | 0.05% | | 0.01% | | ± | | 0.01% | | 0.06% | | ± | | 0.02% | |
|  | {YYYYY}3 | 0.49% | ± | | 0.00% | | 0.01% | | ± | | 0.01% | | 0.00% | | ± | | 0.00% | | 0.00% | | ± | | 0.00% | |
|  | {YYYYY}4 | 0.18% | ± | | 0.02% | | 0.01% | | ± | | 0.01% | | 0.00% | | ± | | 0.00% | | 0.01% | | ± | | 0.02% | |
|  | {11111} | 2.21% | ± | | 0.02% | | 0.06% | | ± | | 0.02% | | 0.02% | | ± | | 0.01% | | 0.04% | | ± | | 0.03% | |
| Lys-431 | {000000} | 98.94% | ± | | 0.21% | | 99.59% | | ± | | 0.14% | | 99.64% | | ± | | 0.10% | | 99.71% | | ± | | 0.07% | |
|  | {YYYYYY}1 | 0.06% | ± | | 0.08% | | 0.12% | | ± | | 0.10% | | 0.00% | | ± | | 0.00% | | 0.00% | | ± | | 0.00% | |
|  | {YYYYYY}2 | 0.46% | ± | | 0.05% | | 0.22% | | ± | | 0.03% | | 0.33% | | ± | | 0.09% | | 0.28% | | ± | | 0.05% | |
|  | {YYYYYY}3 | 0.01% | ± | | 0.01% | | 0.00% | | ± | | 0.00% | | 0.00% | | ± | | 0.00% | | 0.00% | | ± | | 0.00% | |
|  | {YYYYYY}4 | 0.06% | ± | | 0.02% | | 0.00% | | ± | | 0.00% | | 0.00% | | ± | | 0.00% | | 0.00% | | ± | | 0.00% | |
|  | {YYYYYY}5 | 0.40% | ± | | 0.12% | | 0.04% | | ± | | 0.04% | | 0.03% | | ± | | 0.03% | | 0.01% | | ± | | 0.01% | |
|  | {111111} | 0.07% | ± | | 0.06% | | 0.03% | | ± | | 0.02% | | 0.00% | | ± | | 0.00% | | 0.00% | | ± | | 0.01% | |
|  |  | *EIEC* 4608-58 | | | | | | | | | | *Caco-2 cells infected with EIEC* 4608-58 | | | | | | | | | | | |  |
|  |  | wildtype | | | | Δ*uhpT, ptsG, manXYZ* | | | | | | wildtype | | | | | | Δ*uhpT, ptsG, manXYZ* | | | | | |  |
| Gly-246 | {00} | 98.65% | ± | 0.02% | | 99.39% | | ± | | 0.03% | | 98.15% | | ± | | 0.01% | | 98.36% | | ± | | 0.06% | |  |
|  | {YY}1 | 0.00% | ± | 0.00% | | 0.00% | | ± | | 0.00% | | 0.00% | | ± | | 0.00% | | 0.00% | | ± | | 0.00% | |  |
|  | {11} | 1.35% | ± | 0.02% | | 0.61% | | ± | | 0.03% | | 1.85% | | ± | | 0.01% | | 1.64% | | ± | | 0.06% | |  |
| Thr-404 | {0000} | 98.84% | ± | 0.09% | | 98.77% | | ± | | 0.52% | | 99.34% | | ± | | 0.33% | | 98.34% | | ± | | 0.62% | |  |
|  | {YYYY}1 | 0.93% | ± | 0.09% | | 1.19% | | ± | | 0.49% | | 0.64% | | ± | | 0.32% | | 1.64% | | ± | | 0.65% | |  |
|  | {YYYY}2 | 0.00% | ± | 0.00% | | 0.00% | | ± | | 0.00% | | 0.00% | | ± | | 0.00% | | 0.00% | | ± | | 0.00% | |  |
|  | {YYYY}3 | 0.19% | ± | 0.07% | | 0.01% | | ± | | 0.01% | | 0.00% | | ± | | 0.00% | | 0.00% | | ± | | 0.00% | |  |
|  | {1111} | 0.03% | ± | 0.03% | | 0.03% | | ± | | 0.03% | | 0.03% | | ± | | 0.03% | | 0.02% | | ± | | 0.03% | |  |
| Pro-286 | {00000} | 97.96% | ± | 0.35% | | 96.85% | | ± | | 0.70% | | 96.77% | | ± | | 1.12% | | 95.82% | | ± | | 0.25% | |  |
|  | {YYYYY}1 | 0.00% | ± | 0.00% | | 0.04% | | ± | | 0.05% | | 0.07% | | ± | | 0.06% | | 0.11% | | ± | | 0.15% | |  |
|  | {YYYYY}2 | 1.68% | ± | 0.40% | | 1.50% | | ± | | 0.13% | | 2.35% | | ± | | 0.43% | | 2.16% | | ± | | 0.51% | |  |
|  | {YYYYY}3 | 0.36% | ± | 0.08% | | 0.27% | | ± | | 0.09% | | 0.43% | | ± | | 0.07% | | 0.43% | | ± | | 0.10% | |  |
|  | {YYYYY}4 | 0.00% | ± | 0.00% | | 1.26% | | ± | | 0.77% | | 0.33% | | ± | | 0.56% | | 1.30% | | ± | | 0.30% | |  |
|  | {11111} | 0.00% | ± | 0.00% | | 0.08% | | ± | | 0.04% | | 0.06% | | ± | | 0.10% | | 0.18% | | ± | | 0.06% | |  |
| His-440 | {000000} | 98.48% | ± | 0.06% | | 99.47% | | ± | | 0.07% | | 99.78% | | ± | | 0.06% | | 99.76% | | ± | | 0.11% | |  |
|  | {YYYYYY}1 | 0.00% | ± | 0.00% | | 0.00% | | ± | | 0.00% | | 0.00% | | ± | | 0.00% | | 0.00% | | ± | | 0.00% | |  |
|  | {YYYYYY}2 | 0.57% | ± | 0.11% | | 0.34% | | ± | | 0.06% | | 0.16% | | ± | | 0.05% | | 0.17% | | ± | | 0.10% | |  |
|  | {YYYYYY}3 | 0.01% | ± | 0.01% | | 0.00% | | ± | | 0.00% | | 0.00% | | ± | | 0.00% | | 0.00% | | ± | | 0.00% | |  |
|  | {YYYYYY}4 | 0.08% | ± | 0.05% | | 0.04% | | ± | | 0.03% | | 0.00% | | ± | | 0.00% | | 0.00% | | ± | | 0.00% | |  |
|  | {YYYYYY}5 | 0.82% | ± | 0.02% | | 0.15% | | ± | | 0.05% | | 0.04% | | ± | | 0.03% | | 0.07% | | ± | | 0.03% | |  |
|  | {111111} | 0.04% | ± | 0.05% | | 0.00% | | ± | | 0.00% | | 0.02% | | ± | | 0.02% | | 0.01% | | ± | | 0.01% | |  |
| Phe-336 | {000000000} | 99.03% | ± | 0.51% | | 98.64% | | ± | | 0.49% | | 99.33% | | ± | | 0.55% | | 98.86% | | ± | | 0.39% | |  |
|  | {YYYYYYYYY}1 | 0.64% | ± | 0.52% | | 1.14% | | ± | | 0.42% | | 0.61% | | ± | | 0.53% | | 1.04% | | ± | | 0.36% | |  |
|  | {YYYYYYYYY}2 | 0.00% | ± | 0.00% | | 0.00% | | ± | | 0.00% | | 0.00% | | ± | | 0.00% | | 0.00% | | ± | | 0.00% | |  |
|  | {YYYYYYYYY}3 | 0.00% | ± | 0.00% | | 0.04% | | ± | | 0.04% | | 0.01% | | ± | | 0.01% | | 0.02% | | ± | | 0.02% | |  |
|  | {YYYYYYYYY}4 | 0.00% | ± | 0.00% | | 0.00% | | ± | | 0.00% | | 0.00% | | ± | | 0.00% | | 0.01% | | ± | | 0.01% | |  |
|  | {YYYYYYYYY}5 | 0.01% | ± | 0.01% | | 0.05% | | ± | | 0.04% | | 0.00% | | ± | | 0.01% | | 0.01% | | ± | | 0.02% | |  |
|  | {YYYYYYYYY}6 | 0.07% | ± | 0.06% | | 0.06% | | ± | | 0.06% | | 0.02% | | ± | | 0.00% | | 0.01% | | ± | | 0.02% | |  |
|  | {YYYYYYYYY}7 | 0.01% | ± | 0.02% | | 0.01% | | ± | | 0.01% | | 0.00% | | ± | | 0.01% | | 0.02% | | ± | | 0.02% | |  |
|  | {YYYYYYYYY}8 | 0.05% | ± | 0.02% | | 0.02% | | ± | | 0.02% | | 0.02% | | ± | | 0.00% | | 0.02% | | ± | | 0.01% | |  |
|  | {111111111} | 0.20% | ± | 0.02% | | 0.03% | | ± | | 0.03% | | 0.01% | | ± | | 0.01% | | 0.01% | | ± | | 0.02% | |  |

|  |  | *EIEC* 4608-58 | | | | | | *Caco-2 cells infected with EIEC* 4608-58 | | | | | |
| --- | --- | --- | --- | --- | --- | --- | --- | --- | --- | --- | --- | --- | --- |
|  |  | wildtype | | | Δ*uhpT, ptsG, manXYZ* | | | wildtype | | | Δ*uhpT, ptsG, manXYZ* | | |
| Tyr-466 | {000000000} | 99.14% | ± | 0.08% | 98.98% | ± | 1.10% |  |  |  | 99.75% | ± | 0.11% |
|  | {YYYYYYYYY}1 | 0.00% | ± | 0.00% | 0.00% | ± | 0.00% |  |  |  | 0.00% | ± | 0.00% |
|  | {YYYYYYYYY}2 | 0.00% | ± | 0.00% | 0.00% | ± | 0.00% |  |  |  | 0.14% | ± | 0.20% |
|  | {YYYYYYYYY}3 | 0.01% | ± | 0.01% | 0.01% | ± | 0.02% |  |  |  | 0.02% | ± | 0.03% |
|  | {YYYYYYYYY}4 | 0.00% | ± | 0.00% | 0.01% | ± | 0.01% |  |  |  | 0.00% | ± | 0.00% |
|  | {YYYYYYYYY}5 | 0.01% | ± | 0.01% | 0.00% | ± | 0.00% |  |  |  | 0.09% | ± | 0.12% |
|  | {YYYYYYYYY}6 | 0.00% | ± | 0.00% | 0.00% | ± | 0.00% |  |  |  | 0.00% | ± | 0.00% |
|  | {YYYYYYYYY}7 | 0.00% | ± | 0.00% | 0.00% | ± | 0.00% |  |  |  | 0.00% | ± | 0.00% |
|  | {YYYYYYYYY}8 | 0.05% | ± | 0.03% | 0.02% | ± | 0.02% |  |  |  | 0.00% | ± | 0.00% |
|  | {111111111} | 0.79% | ± | 0.08% | 0.98% | ± | 1.11% |  |  |  | 0.00% | ± | 0.00% |
| Ile-200 | {X00000} | 99.81% | ± | 0.03% | 99.84% | ± | 0.04% | 99.91% | ± | 0.01% | 99.91% | ± | 0.03% |
|  | {XYYYYY}1 | 0.00% | ± | 0.00% | 0.00% | ± | 0.00% | 0.00% | ± | 0.00% | 0.00% | ± | 0.00% |
|  | {XYYYYY}2 | 0.09% | ± | 0.02% | 0.05% | ± | 0.00% | 0.06% | ± | 0.00% | 0.03% | ± | 0.01% |
|  | {XYYYYY}3 | 0.04% | ± | 0.01% | 0.05% | ± | 0.01% | 0.02% | ± | 0.00% | 0.03% | ± | 0.01% |
|  | {XYYYYY}4 | 0.01% | ± | 0.00% | 0.01% | ± | 0.01% | 0.00% | ± | 0.00% | 0.00% | ± | 0.00% |
|  | {X11111} | 0.05% | ± | 0.01% | 0.06% | ± | 0.03% | 0.01% | ± | 0.02% | 0.02% | ± | 0.03% |
| Leu-274 | {X00000} | 99.96% | ± | 0.02% | 99.98% | ± | 0.01% | 99.98% | ± | 0.01% | 99.98% | ± | 0.01% |
|  | {XYYYYY}1 | 0.00% | ± | 0.00% | 0.00% | ± | 0.00% | 0.00% | ± | 0.00% | 0.00% | ± | 0.00% |
|  | {XYYYYY}2 | 0.00% | ± | 0.00% | 0.00% | ± | 0.00% | 0.00% | ± | 0.00% | 0.00% | ± | 0.00% |
|  | {XYYYYY}3 | 0.00% | ± | 0.00% | 0.00% | ± | 0.00% | 0.00% | ± | 0.00% | 0.00% | ± | 0.00% |
|  | {XYYYYY}4 | 0.02% | ± | 0.01% | 0.00% | ± | 0.01% | 0.02% | ± | 0.01% | 0.02% | ± | 0.01% |
|  | {X11111} | 0.01% | ± | 0.01% | 0.01% | ± | 0.01% | 0.01% | ± | 0.01% | 0.00% | ± | 0.00% |

|  |  | *Stm* 14028 | | | | | | | | | | | | | | | | | | | | | | | | | | | | | | | | | | |
| --- | --- | --- | --- | --- | --- | --- | --- | --- | --- | --- | --- | --- | --- | --- | --- | --- | --- | --- | --- | --- | --- | --- | --- | --- | --- | --- | --- | --- | --- | --- | --- | --- | --- | --- | --- | --- |
|  |  | wildtype | | | | | Δ*uhpT*, *ptsG*, *manXYZ* | | | | | | Δ*ptsG, manXYZ* | | | | | |  Δ*uhpT* | | | | | | wildtype | | | | | | Δ*uhpT, ptsG*, *manXYZ* | | | | | |
| Ala-260 | {000} | 77.62% | ± | | 0.11% | | 87.32% | | ± | | 0.13% | | 87.22% | | ± | | 0.24% | | 77.58% | | ± | | 0.24% | | 55.04% | | ± | | 0.23% | | 86.75% | | | ± | 0.73% | |
|  | {YYY}1 | 0.55% | ± | | 0.10% | | 0.71% | | ± | | 0.04% | | 0.70% | | ± | | 0.03% | | 0.48% | | ± | | 0.03% | | 0.32% | | ± | | 0.34% | | 0.33% | | | ± | 0.27% | |
|  | {YYY}2 | 1.07% | ± | | 0.04% | | 0.94% | | ± | | 0.03% | | 0.88% | | ± | | 0.03% | | 1.02% | | ± | | 0.03% | | 2.08% | | ± | | 0.17% | | 0.80% | | | ± | 0.17% | |
|  | {111} | 20.77% | ± | | 0.04% | | 11.03% | | ± | | 0.10% | | 11.20% | | ± | | 0.19% | | 20.92% | | ± | | 0.19% | | 42.57% | | ± | | 0.65% | | 12.12% | | | ± | 0.36% | |
| Asp-418 | {0000} | 89.14% | ± | | 0.35% | | 93.53% | | ± | | 0.36% | | 93.34% | | ± | | 0.32% | | 88.53% | | ± | | 0.32% | | 75.99% | | ± | | 2.08% | | 93.46% | | | ± | 0.88% | |
|  | {YYYY}1 | 0.16% | ± | | 0.28% | | 0.55% | | ± | | 0.31% | | 0.92% | | ± | | 0.32% | | 0.37% | | ± | | 0.32% | | 0.47% | | ± | | 0.82% | | 0.62% | | | ± | 0.42% | |
|  | {YYYY}2 | 2.92% | ± | | 0.07% | | 2.76% | | ± | | 0.09% | | 2.57% | | ± | | 0.10% | | 2.70% | | ± | | 0.10% | | 5.34% | | ± | | 0.44% | | 2.51% | | | ± | 0.81% | |
|  | {YYYY}3 | 6.54% | ± | | 0.11% | | 2.59% | | ± | | 0.18% | | 2.67% | | ± | | 0.05% | | 7.10% | | ± | | 0.05% | | 15.42% | | ± | | 1.08% | | 2.84% | | | ± | 0.24% | |
|  | {1111} | 1.23% | ± | | 0.06% | | 0.57% | | ± | | 0.02% | | 0.49% | | ± | | 0.03% | | 1.30% | | ± | | 0.03% | | 2.78% | | ± | | 0.48% | | 0.57% | | | ± | 0.02% | |
| Glu-432 | {00000} | 88.24% | ± | | 0.25% | | 92.56% | | ± | | 0.01% | | 92.32% | | ± | | 0.17% | | 87.97% | | ± | | 0.17% | | 76.23% | | ± | | 0.40% | | 92.90% | | | ± | 0.53% | |
|  | {YYYYY}1 | 1.07% | ± | | 0.31% | | 1.12% | | ± | | 0.01% | | 1.41% | | ± | | 0.27% | | 0.91% | | ± | | 0.27% | | 0.35% | | ± | | 0.33% | | 0.13% | | | ± | 0.23% | |
|  | {YYYYY}2 | 4.64% | ± | | 0.03% | | 3.45% | | ± | | 0.09% | | 3.48% | | ± | | 0.03% | | 4.83% | | ± | | 0.03% | | 8.46% | | ± | | 0.33% | | 3.70% | | | ± | 0.06% | |
|  | {YYYYY}3 | 1.25% | ± | | 0.04% | | 1.15% | | ± | | 0.05% | | 1.12% | | ± | | 0.11% | | 1.21% | | ± | | 0.11% | | 2.68% | | ± | | 0.51% | | 1.32% | | | ± | 0.13% | |
|  | {YYYYY}4 | 2.94% | ± | | 0.02% | | 1.16% | | ± | | 0.01% | | 1.12% | | ± | | 0.02% | | 3.03% | | ± | | 0.02% | | 7.17% | | ± | | 0.69% | | 1.06% | | | ± | 0.31% | |
|  | {11111} | 1.86% | ± | | 0.02% | | 0.56% | | ± | | 0.02% | | 0.54% | | ± | | 0.03% | | 2.04% | | ± | | 0.03% | | 5.11% | | ± | | 0.51% | | 0.89% | | | ± | 0.36% | |
| Ser-390 | {000} | 91.14% | ± | | 0.65% | | 95.78% | | ± | | 0.59% | | 91.21% | | ± | | 0.59% | | 95.58% | | ± | | 0.59% | | 72.87% | | ± | | 1.57% | | 96.06% | | | ± | 0.95% | |
|  | {YYY}1 | 2.29% | ± | | 0.47% | | 1.75% | | ± | | 0.45% | | 2.12% | | ± | | 0.34% | | 1.84% | | ± | | 0.34% | | 8.35% | | ± | | 4.29% | | 0.84% | | | ± | 1.09% | |
|  | {YYY}2 | 1.53% | ± | | 0.15% | | 1.05% | | ± | | 0.19% | | 1.38% | | ± | | 0.20% | | 1.10% | | ± | | 0.20% | | 0.51% | | ± | | 0.88% | | 0.23% | | | ± | 0.20% | |
|  | {111} | 5.04% | ± | | 0.04% | | 1.43% | | ± | | 0.04% | | 5.29% | | ± | | 0.14% | | 1.48% | | ± | | 0.14% | | 18.27% | | ± | | 4.06% | | 2.86% | | | ± | 0.70% | |
| Val-288 | {00000} | 87.69% | ± | | 0.02% | | 95.91% | | ± | | 0.10% | | 87.46% | | ± | | 0.25% | | 95.37% | | ± | | 0.25% | | 67.45% | | ± | | 1.05% | | 96.66% | | | ± | 1.77% | |
|  | {YYYYY}1 | 0.40% | ± | | 0.18% | | 0.60% | | ± | | 0.10% | | 0.33% | | ± | | 0.13% | | 0.54% | | ± | | 0.13% | | 0.95% | | ± | | 0.24% | | 0.00% | | | ± | 0.00% | |
|  | {YYYYY}2 | 1.31% | ± | | 0.12% | | 0.83% | | ± | | 0.08% | | 1.18% | | ± | | 0.05% | | 0.85% | | ± | | 0.05% | | 3.01% | | ± | | 1.03% | | 0.51% | | | ± | 0.72% | |
|  | {YYYYY}3 | 1.54% | ± | | 0.01% | | 0.92% | | ± | | 0.08% | | 1.48% | | ± | | 0.04% | | 1.03% | | ± | | 0.04% | | 2.64% | | ± | | 0.95% | | 1.27% | | | ± | 1.21% | |
|  | {YYYYY}4 | 0.63% | ± | | 0.03% | | 0.31% | | ± | | 0.02% | | 0.67% | | ± | | 0.04% | | 0.34% | | ± | | 0.04% | | 2.25% | | ± | | 0.87% | | 0.38% | | | ± | 0.65% | |
|  | {11111} | 8.44% | ± | | 0.06% | | 1.42% | | ± | | 0.05% | | 8.90% | | ± | | 0.13% | | 1.86% | | ± | | 0.13% | | 23.70% | | ± | | 0.28% | | 1.18% | | | ± | 0.55% | |
| Lys-431 | {000000} | 90.93% | ± | | 3.54% | | 97.92% | | ± | | 0.23% | | 93.71% | | ± | | 0.34% | | 98.01% | | ± | | 0.34% | | 83.11% | | ± | | 1.93% | | 96.07% | | | ± | 3.42% | |
|  | {YYYYYY}1 | 1.19% | ± | | 1.17% | | 0.15% | | ± | | 0.25% | | 0.12% | | ± | | 0.20% | | 0.00% | | ± | | 0.20% | | 1.42% | | ± | | 1.57% | | 0.00% | | | ± | 0.00% | |
|  | {YYYYYY}2 | 1.46% | ± | | 0.54% | | 0.21% | | ± | | 0.17% | | 0.98% | | ± | | 0.17% | | 0.38% | | ± | | 0.17% | | 1.73% | | ± | | 3.00% | | 0.31% | | | ± | 0.54% | |
|  | {YYYYYY}3 | 1.89% | ± | | 0.54% | | 1.04% | | ± | | 0.12% | | 1.71% | | ± | | 0.30% | | 0.95% | | ± | | 0.30% | | 0.85% | | ± | | 1.47% | | 0.73% | | | ± | 0.88% | |
|  | {YYYYYY}4 | 0.74% | ± | | 0.42% | | 0.15% | | ± | | 0.13% | | 0.46% | | ± | | 0.29% | | 0.10% | | ± | | 0.29% | | 0.00% | | ± | | 0.00% | | 0.00% | | | ± | 0.00% | |
|  | {YYYYYY}5 | 2.49% | ± | | 0.52% | | 0.32% | | ± | | 0.03% | | 2.16% | | ± | | 0.11% | | 0.35% | | ± | | 0.11% | | 2.39% | | ± | | 4.00% | | 1.80% | | | ± | 1.73% | |
|  | {111111} | 1.31% | ± | | 0.58% | | 0.21% | | ± | | 0.04% | | 0.87% | | ± | | 0.07% | | 0.21% | | ± | | 0.07% | | 10.50% | | ± | | 6.11% | | 1.09% | | | ± | 1.69% | |
|  |  | *Stm* 14028 | | | | | | | | | | | | | | | | | | | | | | | | | | | | | | | | | |  |
|  |  | wildtype | | | | Δ*uhpT*, *ptsG*, *manXYZ* | | | | | | Δ*ptsG, manXYZ* | | | | | |  Δ*uhpT* | | | | | | wildtype | | | | | | Δ*uhpT, ptsG*, *manXYZ* | | | | | |  |
| Gly-246 | {00} | 93.64% | ± | 0.07% | | 97.95% | | ± | | 0.10% | | 98.01% | | ± | | 0.10% | | 93.55% | | ± | | 0.10% | | 84.00% | | ± | | 0.22% | | 98.93% | | ± | 0.54% | | |  |
|  | {YY}1 | 0.89% | ± | 0.07% | | 0.44% | | ± | | 0.07% | | 0.40% | | ± | | 0.09% | | 0.96% | | ± | | 0.09% | | 1.87% | | ± | | 0.39% | | 0.00% | | ± | 0.00% | | |  |
|  | {11} | 5.47% | ± | 0.01% | | 1.61% | | ± | | 0.03% | | 1.59% | | ± | | 0.01% | | 5.50% | | ± | | 0.01% | | 14.14% | | ± | | 0.35% | | 1.07% | | ± | 0.54% | | |  |
| Thr-404 | {0000} | 95.72% | ± | 0.14% | | 98.57% | | ± | | 0.30% | | 95.74% | | ± | | 0.40% | | 98.60% | | ± | | 0.40% | |  | |  | |  | | 95.86% | | ± | 3.30% | | |  |
|  | {YYYY}1 | 0.09% | ± | 0.16% | | 0.18% | | ± | | 0.31% | | 0.00% | | ± | | 0.00% | | 0.32% | | ± | | 0.00% | |  | |  | |  | | 0.47% | | ± | 0.47% | | |  |
|  | {YYYY}2 | 1.42% | ± | 0.20% | | 0.96% | | ± | | 0.48% | | 1.47% | | ± | | 0.58% | | 0.94% | | ± | | 0.58% | |  | |  | |  | | 3.29% | | ± | 2.87% | | |  |
|  | {YYYY}3 | 2.18% | ± | 0.28% | | 0.15% | | ± | | 0.26% | | 2.26% | | ± | | 0.32% | | 0.00% | | ± | | 0.32% | |  | |  | |  | | 0.00% | | ± | 0.00% | | |  |
|  | {1111} | 0.59% | ± | 0.07% | | 0.14% | | ± | | 0.07% | | 0.53% | | ± | | 0.07% | | 0.14% | | ± | | 0.07% | |  | |  | |  | | 0.38% | | ± | 0.34% | | |  |
| Pro-286 | {00000} | 94.70% | ± | 0.07% | | 95.56% | | ± | | 1.12% | | 94.33% | | ± | | 0.97% | | 95.77% | | ± | | 0.97% | | 89.83% | | ± | | 2.19% | | 93.73% | | ± | 2.57% | | |  |
|  | {YYYYY}1 | 0.73% | ± | 0.06% | | 0.53% | | ± | | 0.46% | | 0.48% | | ± | | 0.46% | | 0.59% | | ± | | 0.46% | | 0.00% | | ± | | 0.00% | | 0.64% | | ± | 1.11% | | |  |
|  | {YYYYY}2 | 2.26% | ± | 0.09% | | 2.03% | | ± | | 0.33% | | 2.26% | | ± | | 0.18% | | 1.85% | | ± | | 0.18% | | 4.31% | | ± | | 0.41% | | 1.44% | | ± | 0.76% | | |  |
|  | {YYYYY}3 | 0.46% | ± | 0.02% | | 0.38% | | ± | | 0.14% | | 0.44% | | ± | | 0.05% | | 0.44% | | ± | | 0.05% | | 1.48% | | ± | | 0.38% | | 1.44% | | ± | 1.32% | | |  |
|  | {YYYYY}4 | 1.17% | ± | 0.18% | | 1.35% | | ± | | 0.44% | | 1.76% | | ± | | 0.49% | | 1.15% | | ± | | 0.49% | | 2.32% | | ± | | 2.40% | | 1.30% | | ± | 0.58% | | |  |
|  | {11111} | 0.67% | ± | 0.04% | | 0.15% | | ± | | 0.09% | | 0.73% | | ± | | 0.06% | | 0.20% | | ± | | 0.06% | | 2.05% | | ± | | 0.80% | | 1.45% | | ± | 1.31% | | |  |
| His-440 | {000000} | 97.04% | ± | 0.44% | | 98.48% | | ± | | 0.22% | | 98.96% | | ± | | 0.30% | | 96.50% | | ± | | 0.30% | | 88.00% | | ± | | 4.60% | |  | |  |  | | |  |
|  | {YYYYYY}1 | 0.43% | ± | 0.48% | | 0.58% | | ± | | 0.52% | | 0.25% | | ± | | 0.03% | | 0.17% | | ± | | 0.03% | | 0.80% | | ± | | 1.39% | |  | |  |  | | |  |
|  | {YYYYYY}2 | 0.08% | ± | 0.13% | | 0.02% | | ± | | 0.04% | | 0.00% | | ± | | 0.09% | | 0.08% | | ± | | 0.09% | | 0.66% | | ± | | 1.14% | |  | |  |  | | |  |
|  | {YYYYYY}3 | 0.00% | ± | 0.00% | | 0.00% | | ± | | 0.00% | | 0.04% | | ± | | 0.07% | | 0.08% | | ± | | 0.07% | | 0.76% | | ± | | 1.32% | |  | |  |  | | |  |
|  | {YYYYYY}4 | 0.12% | ± | 0.11% | | 0.10% | | ± | | 0.18% | | 0.02% | | ± | | 0.19% | | 0.19% | | ± | | 0.19% | | 1.95% | | ± | | 1.70% | |  | |  |  | | |  |
|  | {YYYYYY}5 | 1.25% | ± | 0.55% | | 0.76% | | ± | | 0.26% | | 0.50% | | ± | | 0.18% | | 1.76% | | ± | | 0.18% | | 5.64% | | ± | | 3.58% | |  | |  |  | | |  |
|  | {111111} | 1.08% | ± | 0.14% | | 0.05% | | ± | | 0.07% | | 0.22% | | ± | | 0.20% | | 1.22% | | ± | | 0.20% | | 2.19% | | ± | | 2.91% | |  | |  |  | | |  |
| Phe-336 | {000000000} | 97.79% | ± | 0.06% | | 98.93% | | ± | | 0.32% | | 97.37% | | ± | | 0.23% | | 99.08% | | ± | | 0.23% | | 89.98% | | ± | | 3.85% | | 90.09% | | ± | 6.70% | | |  |
|  | {YYYYYYYYY}1 | 0.00% | ± | 0.00% | | 0.07% | | ± | | 0.12% | | 0.09% | | ± | | 0.16% | | 0.20% | | ± | | 0.16% | | 0.43% | | ± | | 0.74% | | 1.23% | | ± | 2.14% | | |  |
|  | {YYYYYYYYY}2 | 0.11% | ± | 0.10% | | 0.15% | | ± | | 0.11% | | 0.04% | | ± | | 0.07% | | 0.00% | | ± | | 0.07% | | 0.48% | | ± | | 0.82% | | 1.85% | | ± | 3.10% | | |  |
|  | {YYYYYYYYY}3 | 0.09% | ± | 0.13% | | 0.02% | | ± | | 0.03% | | 0.03% | | ± | | 0.06% | | 0.04% | | ± | | 0.06% | | 0.00% | | ± | | 0.00% | | 0.00% | | ± | 0.00% | | |  |
|  | {YYYYYYYYY}4 | 0.07% | ± | 0.13% | | 0.27% | | ± | | 0.17% | | 0.20% | | ± | | 0.21% | | 0.04% | | ± | | 0.21% | | 1.55% | | ± | | 2.68% | | 2.29% | | ± | 3.96% | | |  |
|  | {YYYYYYYYY}5 | 0.04% | ± | 0.07% | | 0.01% | | ± | | 0.01% | | 0.05% | | ± | | 0.04% | | 0.07% | | ± | | 0.04% | | 0.33% | | ± | | 0.57% | | 1.56% | | ± | 2.70% | | |  |
|  | {YYYYYYYYY}6 | 0.15% | ± | 0.05% | | 0.12% | | ± | | 0.09% | | 0.21% | | ± | | 0.14% | | 0.13% | | ± | | 0.14% | | 0.66% | | ± | | 1.14% | | 0.00% | | ± | 0.00% | | |  |
|  | {YYYYYYYYY}7 | 0.14% | ± | 0.04% | | 0.09% | | ± | | 0.07% | | 0.06% | | ± | | 0.05% | | 0.09% | | ± | | 0.05% | | 0.24% | | ± | | 0.42% | | 0.00% | | ± | 0.00% | | |  |
|  | {YYYYYYYYY}8 | 0.25% | ± | 0.04% | | 0.16% | | ± | | 0.18% | | 0.35% | | ± | | 0.07% | | 0.09% | | ± | | 0.07% | | 0.92% | | ± | | 1.22% | | 2.07% | | ± | 1.81% | | |  |
|  | {111111111} | 1.36% | ± | 0.14% | | 0.18% | | ± | | 0.06% | | 1.60% | | ± | | 0.15% | | 0.27% | | ± | | 0.15% | | 5.42% | | ± | | 1.76% | | 0.91% | | ± | 1.52% | | |  |

|  |  | *Stm* 14028 | | | | | | | | | | | | | | | | | |
| --- | --- | --- | --- | --- | --- | --- | --- | --- | --- | --- | --- | --- | --- | --- | --- | --- | --- | --- | --- |
|  |  | wildtype | | | Δ*uhpT*, *ptsG*, *manXYZ* | | | Δ*ptsG, manXYZ* | | |  Δ*uhpT* | | | wildtype | | | Δ*uhpT, ptsG*, *manXYZ* | | |
| Tyr-466 | {000000000} | 99.73% | ± | 0.09% | 99.88% | ± | 0.05% | 98.58% | ± | 1.26% | 99.82% | ± | 1.26% | 82.96% | ± | 6.24% | 93.30% | ± | 7.92% |
|  | {YYYYYYYYY}1 | 0.00% | ± | 0.00% | 0.00% | ± | 0.00% | 0.20% | ± | 0.35% | 0.00% | ± | 0.35% | 0.00% | ± | 0.00% | 2.40% | ± | 4.16% |
|  | {YYYYYYYYY}2 | 0.00% | ± | 0.00% | 0.00% | ± | 0.00% | 0.00% | ± | 0.00% | 0.00% | ± | 0.00% | 3.02% | ± | 5.23% | 0.00% | ± | 0.00% |
|  | {YYYYYYYYY}3 | 0.01% | ± | 0.01% | 0.01% | ± | 0.01% | 0.06% | ± | 0.10% | 0.00% | ± | 0.10% | 0.52% | ± | 0.90% | 0.00% | ± | 0.00% |
|  | {YYYYYYYYY}4 | 0.00% | ± | 0.00% | 0.00% | ± | 0.00% | 0.00% | ± | 0.00% | 0.00% | ± | 0.00% | 0.83% | ± | 1.35% | 1.06% | ± | 1.50% |
|  | {YYYYYYYYY}5 | 0.00% | ± | 0.00% | 0.00% | ± | 0.01% | 0.00% | ± | 0.00% | 0.00% | ± | 0.00% | 2.50% | ± | 1.87% | 0.14% | ± | 0.24% |
|  | {YYYYYYYYY}6 | 0.12% | ± | 0.14% | 0.11% | ± | 0.05% | 0.20% | ± | 0.19% | 0.12% | ± | 0.19% | 2.84% | ± | 2.77% | 1.75% | ± | 3.00% |
|  | {YYYYYYYYY}7 | 0.13% | ± | 0.06% | 0.00% | ± | 0.00% | 0.22% | ± | 0.21% | 0.05% | ± | 0.21% | 0.87% | ± | 1.50% | 0.86% | ± | 1.49% |
|  | {YYYYYYYYY}8 | 0.01% | ± | 0.03% | 0.00% | ± | 0.00% | 0.09% | ± | 0.15% | 0.00% | ± | 0.15% | 3.29% | ± | 2.26% | 0.50% | ± | 0.87% |
|  | {111111111} | 0.00% | ± | 0.00% | 0.00% | ± | 0.00% | 0.66% | ± | 1.14% | 0.00% | ± | 1.14% | 3.19% | ± | 1.16% | 0.00% | ± | 0.00% |
| Ile-274 | {X00000} | 97.84% | ± | 0.14% | 98.35% | ± | 0.29% | 98.18% | ± | 0.12% | 97.83% | ± | 0.12% | 98.16% | ± | 0.86% | 98.96% | ± | 0.47% |
|  | {XYYYYY}1 | 1.49% | ± | 0.21% | 1.58% | ± | 0.30% | 1.77% | ± | 0.20% | 1.54% | ± | 0.20% | 0.00% | ± | 0.00% | 0.41% | ± | 0.48% |
|  | {XYYYYY}2 | 0.26% | ± | 0.13% | 0.00% | ± | 0.00% | 0.00% | ± | 0.17% | 0.20% | ± | 0.17% | 1.00% | ± | 0.51% | 0.28% | ± | 0.48% |
|  | {XYYYYY}3 | 0.06% | ± | 0.02% | 0.03% | ± | 0.03% | 0.02% | ± | 0.05% | 0.06% | ± | 0.05% | 0.10% | ± | 0.17% | 0.00% | ± | 0.00% |
|  | {XYYYYY}4 | 0.22% | ± | 0.02% | 0.02% | ± | 0.02% | 0.03% | ± | 0.01% | 0.24% | ± | 0.01% | 0.34% | ± | 0.31% | 0.24% | ± | 0.25% |
|  | {X11111} | 0.12% | ± | 0.02% | 0.01% | ± | 0.00% | 0.00% | ± | 0.02% | 0.13% | ± | 0.02% | 0.40% | ± | 0.68% | 0.12% | ± | 0.21% |
| Leu-200 | {X00000} | 99.32% | ± | 0.05% | 99.80% | ± | 0.04% | 99.31% | ± | 0.05% | 99.77% | ± | 0.05% | 98.36% | ± | 0.31% | 99.68% | ± | 0.21% |
|  | {XYYYYY}1 | 0.18% | ± | 0.04% | 0.13% | ± | 0.01% | 0.18% | ± | 0.02% | 0.18% | ± | 0.02% | 0.00% | ± | 0.00% | 0.00% | ± | 0.00% |
|  | {XYYYYY}2 | 0.04% | ± | 0.07% | 0.02% | ± | 0.03% | 0.04% | ± | 0.04% | 0.00% | ± | 0.04% | 0.24% | ± | 0.15% | 0.11% | ± | 0.20% |
|  | {XYYYYY}3 | 0.09% | ± | 0.01% | 0.01% | ± | 0.00% | 0.09% | ± | 0.01% | 0.00% | ± | 0.01% | 0.13% | ± | 0.12% | 0.05% | ± | 0.06% |
|  | {XYYYYY}4 | 0.06% | ± | 0.01% | 0.01% | ± | 0.00% | 0.06% | ± | 0.01% | 0.02% | ± | 0.01% | 0.21% | ± | 0.13% | 0.10% | ± | 0.18% |
|  | {X11111} | 0.31% | ± | 0.01% | 0.02% | ± | 0.01% | 0.32% | ± | 0.01% | 0.03% | ± | 0.01% | 1.06% | ± | 0.15% | 0.05% | ± | 0.09% |

|  |  | Caco-2 cells infected with *Stm* 14028 | | | | | | | | | | | | | | | | | | | | | | | | | | | | | | | | | | |
| --- | --- | --- | --- | --- | --- | --- | --- | --- | --- | --- | --- | --- | --- | --- | --- | --- | --- | --- | --- | --- | --- | --- | --- | --- | --- | --- | --- | --- | --- | --- | --- | --- | --- | --- | --- | --- |
|  |  | wildtype | | | | | Δ*uhpT, ptsG, manXYZ* | | | | | | Δ*ptsG, manXYZ* | | | | | |  Δ*uhpT* | | | | | | wildtype | | | | | | Δ*uhpT, ptsG, manXYZ* | | | | | |
| Ala-260 | {000} | 89.45% | ± | | 0.07% | | 88.86% | | ± | | 0.11% | | 89.54% | | ± | | 0.01% | | 89.18% | | ± | | 0.01% | | 83.52% | | ± | | 0.46% | | 84.74% | | | ± | 0.07% | |
|  | {YYY}1 | 0.45% | ± | | 0.02% | | 0.44% | | ± | | 0.03% | | 0.49% | | ± | | 0.06% | | 0.35% | | ± | | 0.02% | | 0.32% | | ± | | 0.13% | | 0.26% | | | ± | 0.14% | |
|  | {YYY}2 | 0.55% | ± | | 0.01% | | 0.58% | | ± | | 0.03% | | 0.52% | | ± | | 0.02% | | 0.57% | | ± | | 0.04% | | 0.84% | | ± | | 0.30% | | 0.74% | | | ± | 0.15% | |
|  | {111} | 9.55% | ± | | 0.07% | | 10.13% | | ± | | 0.07% | | 9.45% | | ± | | 0.06% | | 9.90% | | ± | | 0.02% | | 15.31% | | ± | | 0.31% | | 14.26% | | | ± | 0.16% | |
| Asp-418 | {0000} | 96.28% | ± | | 0.07% | | 95.93% | | ± | | 0.19% | | 96.48% | | ± | | 0.10% | | 96.11% | | ± | | 0.23% | | 95.10% | | ± | | 0.19% | | 95.66% | | | ± | 0.29% | |
|  | {YYYY}1 | 0.30% | ± | | 0.15% | | 0.46% | | ± | | 0.22% | | 0.15% | | ± | | 0.13% | | 0.21% | | ± | | 0.20% | | 0.31% | | ± | | 0.22% | | 0.52% | | | ± | 0.38% | |
|  | {YYYY}2 | 2.15% | ± | | 0.06% | | 2.28% | | ± | | 0.04% | | 2.08% | | ± | | 0.05% | | 2.33% | | ± | | 0.04% | | 2.71% | | ± | | 0.18% | | 1.97% | | | ± | 0.25% | |
|  | {YYYY}3 | 1.11% | ± | | 0.08% | | 1.16% | | ± | | 0.05% | | 1.13% | | ± | | 0.06% | | 1.17% | | ± | | 0.04% | | 1.68% | | ± | | 0.22% | | 1.57% | | | ± | 0.32% | |
|  | {1111} | 0.17% | ± | | 0.01% | | 0.18% | | ± | | 0.01% | | 0.16% | | ± | | 0.01% | | 0.17% | | ± | | 0.03% | | 0.20% | | ± | | 0.31% | | 0.27% | | | ± | 0.23% | |
| Glu-432 | {00000} | 88.45% | ± | | 0.42% | | 87.35% | | ± | | 0.22% | | 88.05% | | ± | | 0.29% | | 87.77% | | ± | | 0.16% | | 89.84% | | ± | | 0.09% | | 89.30% | | | ± | 0.23% | |
|  | {YYYYY}1 | 1.49% | ± | | 0.29% | | 1.85% | | ± | | 0.09% | | 1.69% | | ± | | 0.37% | | 1.62% | | ± | | 0.16% | | 0.32% | | ± | | 0.08% | | 0.35% | | | ± | 0.28% | |
|  | {YYYYY}2 | 6.42% | ± | | 0.20% | | 6.97% | | ± | | 0.18% | | 6.64% | | ± | | 0.06% | | 6.78% | | ± | | 0.11% | | 5.83% | | ± | | 0.11% | | 6.22% | | | ± | 0.22% | |
|  | {YYYYY}3 | 1.68% | ± | | 0.07% | | 1.82% | | ± | | 0.04% | | 1.73% | | ± | | 0.04% | | 1.82% | | ± | | 0.07% | | 1.67% | | ± | | 0.10% | | 1.80% | | | ± | 0.15% | |
|  | {YYYYY}4 | 1.45% | ± | | 0.03% | | 1.49% | | ± | | 0.02% | | 1.39% | | ± | | 0.02% | | 1.52% | | ± | | 0.04% | | 1.71% | | ± | | 0.03% | | 1.63% | | | ± | 0.03% | |
|  | {11111} | 0.51% | ± | | 0.02% | | 0.52% | | ± | | 0.00% | | 0.50% | | ± | | 0.01% | | 0.50% | | ± | | 0.02% | | 0.63% | | ± | | 0.05% | | 0.70% | | | ± | 0.04% | |
| Ser-390 | {000} | 97.20% | ± | | 0.78% | | 96.89% | | ± | | 0.89% | | 97.01% | | ± | | 0.57% | | 97.30% | | ± | | 0.78% | | 94.49% | | ± | | 1.24% | | 94.22% | | | ± | 1.85% | |
|  | {YYY}1 | 1.65% | ± | | 0.67% | | 1.85% | | ± | | 0.74% | | 1.79% | | ± | | 0.58% | | 1.62% | | ± | | 0.68% | | 2.50% | | ± | | 0.66% | | 3.09% | | | ± | 1.64% | |
|  | {YYY}2 | 0.59% | ± | | 0.20% | | 0.72% | | ± | | 0.22% | | 0.69% | | ± | | 0.04% | | 0.51% | | ± | | 0.15% | | 1.26% | | ± | | 0.52% | | 0.64% | | | ± | 0.45% | |
|  | {111} | 0.56% | ± | | 0.04% | | 0.54% | | ± | | 0.07% | | 0.51% | | ± | | 0.05% | | 0.57% | | ± | | 0.06% | | 1.75% | | ± | | 0.39% | | 2.05% | | | ± | 0.34% | |
| Val-288 | {00000} | 99.58% | ± | | 0.06% | | 99.62% | | ± | | 0.07% | | 99.59% | | ± | | 0.21% | | 99.66% | | ± | | 0.09% | | 98.85% | | ± | | 0.65% | | 99.05% | | | ± | 0.94% | |
|  | {YYYYY}1 | 0.37% | ± | | 0.07% | | 0.37% | | ± | | 0.07% | | 0.39% | | ± | | 0.21% | | 0.27% | | ± | | 0.06% | | 0.00% | | ± | | 0.00% | | 0.21% | | | ± | 0.34% | |
|  | {YYYYY}2 | 0.00% | ± | | 0.00% | | 0.00% | | ± | | 0.00% | | 0.00% | | ± | | 0.00% | | 0.00% | | ± | | 0.00% | | 0.40% | | ± | | 0.33% | | 0.18% | | | ± | 0.31% | |
|  | {YYYYY}3 | 0.02% | ± | | 0.02% | | 0.01% | | ± | | 0.01% | | 0.02% | | ± | | 0.01% | | 0.02% | | ± | | 0.03% | | 0.13% | | ± | | 0.22% | | 0.15% | | | ± | 0.18% | |
|  | {YYYYY}4 | 0.00% | ± | | 0.00% | | 0.00% | | ± | | 0.00% | | 0.00% | | ± | | 0.00% | | 0.00% | | ± | | 0.00% | | 0.25% | | ± | | 0.19% | | 0.29% | | | ± | 0.50% | |
|  | {11111} | 0.03% | ± | | 0.00% | | 0.00% | | ± | | 0.00% | | 0.00% | | ± | | 0.00% | | 0.05% | | ± | | 0.02% | | 0.38% | | ± | | 0.11% | | 0.12% | | | ± | 0.18% | |
| Lys-431 | {000000} | 99.67% | ± | | 0.10% | | 99.71% | | ± | | 0.09% | | 99.69% | | ± | | 0.07% | | 99.70% | | ± | | 0.12% | | 95.41% | | ± | | 2.16% | | 99.16% | | | ± | 1.46% | |
|  | {YYYYYY}1 | 0.06% | ± | | 0.10% | | 0.04% | | ± | | 0.07% | | 0.14% | | ± | | 0.21% | | 0.00% | | ± | | 0.00% | | 1.10% | | ± | | 1.90% | | 0.02% | | | ± | 0.04% | |
|  | {YYYYYY}2 | 0.00% | ± | | 0.00% | | 0.00% | | ± | | 0.00% | | 0.00% | | ± | | 0.00% | | 0.00% | | ± | | 0.00% | | 0.74% | | ± | | 0.65% | | 0.00% | | | ± | 0.00% | |
|  | {YYYYYY}3 | 0.26% | ± | | 0.03% | | 0.25% | | ± | | 0.03% | | 0.13% | | ± | | 0.14% | | 0.24% | | ± | | 0.05% | | 0.53% | | ± | | 0.92% | | 0.00% | | | ± | 0.00% | |
|  | {YYYYYY}4 | 0.00% | ± | | 0.00% | | 0.00% | | ± | | 0.00% | | 0.00% | | ± | | 0.00% | | 0.00% | | ± | | 0.00% | | 0.00% | | ± | | 0.00% | | 0.00% | | | ± | 0.00% | |
|  | {YYYYYY}5 | 0.00% | ± | | 0.00% | | 0.00% | | ± | | 0.00% | | 0.00% | | ± | | 0.00% | | 0.00% | | ± | | 0.00% | | 0.85% | | ± | | 1.48% | | 0.82% | | | ± | 1.42% | |
|  | {111111} | 0.01% | ± | | 0.02% | | 0.00% | | ± | | 0.00% | | 0.04% | | ± | | 0.06% | | 0.07% | | ± | | 0.08% | | 1.37% | | ± | | 1.28% | | 0.00% | | | ± | 0.00% | |
|  |  | Caco-2 cells infected with *Stm* 14028 | | | | | | | | | | | | | | | | | | | | | | | | | | | | | | | | | |  |
|  |  | wildtype | | | | Δ*uhpT, ptsG, manXYZ* | | | | | | Δ*ptsG, manXYZ* | | | | | |  Δ*uhpT* | | | | | | wildtype | | | | | | Δ*uhpT, ptsG, manXYZ* | | | | | |  |
| Gly-246 | {00} | 98.94% | ± | 0.07% | | 98.75% | | ± | | 0.03% | | 98.82% | | ± | | 0.08% | | 98.80% | | ± | | 0.13% | | 97.38% | | ± | | 0.11% | | 97.38% | | ± | 0.26% | | |  |
|  | {YY}1 | 0.14% | ± | 0.05% | | 0.21% | | ± | | 0.02% | | 0.20% | | ± | | 0.07% | | 0.20% | | ± | | 0.10% | | 0.07% | | ± | | 0.11% | | 0.42% | | ± | 0.12% | | |  |
|  | {11} | 0.92% | ± | 0.02% | | 1.05% | | ± | | 0.02% | | 0.99% | | ± | | 0.02% | | 1.00% | | ± | | 0.04% | | 2.55% | | ± | | 0.00% | | 2.20% | | ± | 0.38% | | |  |
| Thr-404 | {0000} | 99.39% | ± | 0.04% | | 99.21% | | ± | | 0.05% | | 99.29% | | ± | | 0.08% | | 99.41% | | ± | | 0.13% | | 98.20% | | ± | | 1.68% | | 91.54% | | ± | 9.22% | | |  |
|  | {YYYY}1 | 0.00% | ± | 0.00% | | 0.09% | | ± | | 0.16% | | 0.00% | | ± | | 0.00% | | 0.00% | | ± | | 0.00% | | 0.00% | | ± | | 0.00% | | 2.72% | | ± | 2.17% | | |  |
|  | {YYYY}2 | 0.61% | ± | 0.04% | | 0.70% | | ± | | 0.11% | | 0.71% | | ± | | 0.08% | | 0.59% | | ± | | 0.13% | | 0.28% | | ± | | 0.48% | | 0.38% | | ± | 0.65% | | |  |
|  | {YYYY}3 | 0.00% | ± | 0.00% | | 0.00% | | ± | | 0.00% | | 0.00% | | ± | | 0.00% | | 0.00% | | ± | | 0.00% | | 0.68% | | ± | | 1.18% | | 3.60% | | ± | 6.24% | | |  |
|  | {1111} | 0.00% | ± | 0.00% | | 0.00% | | ± | | 0.00% | | 0.00% | | ± | | 0.00% | | 0.00% | | ± | | 0.00% | | 0.83% | | ± | | 1.44% | | 1.77% | | ± | 2.47% | | |  |
| Pro-286 | {00000} | 96.24% | ± | 0.47% | | 95.67% | | ± | | 0.53% | | 96.41% | | ± | | 0.61% | | 96.03% | | ± | | 0.94% | | 95.01% | | ± | | 0.66% | | 93.23% | | ± | 0.53% | | |  |
|  | {YYYYY}1 | 0.52% | ± | 0.05% | | 0.56% | | ± | | 0.14% | | 0.55% | | ± | | 0.06% | | 0.64% | | ± | | 0.27% | | 0.41% | | ± | | 0.27% | | 0.60% | | ± | 0.29% | | |  |
|  | {YYYYY}2 | 2.08% | ± | 0.23% | | 2.10% | | ± | | 0.17% | | 1.89% | | ± | | 0.28% | | 1.98% | | ± | | 0.25% | | 2.67% | | ± | | 0.36% | | 2.56% | | ± | 0.87% | | |  |
|  | {YYYYY}3 | 0.35% | ± | 0.02% | | 0.35% | | ± | | 0.02% | | 0.24% | | ± | | 0.04% | | 0.17% | | ± | | 0.08% | | 0.89% | | ± | | 0.15% | | 0.85% | | ± | 0.91% | | |  |
|  | {YYYYY}4 | 0.75% | ± | 0.29% | | 1.25% | | ± | | 0.47% | | 0.92% | | ± | | 0.24% | | 1.14% | | ± | | 0.46% | | 1.02% | | ± | | 0.46% | | 2.13% | | ± | 1.17% | | |  |
|  | {11111} | 0.06% | ± | 0.03% | | 0.08% | | ± | | 0.02% | | 0.00% | | ± | | 0.00% | | 0.05% | | ± | | 0.09% | | 0.01% | | ± | | 0.01% | | 0.62% | | ± | 0.84% | | |  |
| His-440 | {000000} | 99.47% | ± | 0.26% | | 99.23% | | ± | | 0.42% | | 99.29% | | ± | | 0.16% | | 99.72% | | ± | | 0.13% | | 98.07% | | ± | | 1.84% | | 92.81% | | ± | 2.00% | | |  |
|  | {YYYYYY}1 | 0.47% | ± | 0.27% | | 0.76% | | ± | | 0.43% | | 0.54% | | ± | | 0.18% | | 0.00% | | ± | | 0.00% | | 0.31% | | ± | | 0.54% | | 0.13% | | ± | 0.23% | | |  |
|  | {YYYYYY}2 | 0.00% | ± | 0.00% | | 0.00% | | ± | | 0.00% | | 0.00% | | ± | | 0.00% | | 0.00% | | ± | | 0.00% | | 0.11% | | ± | | 0.19% | | 0.89% | | ± | 1.54% | | |  |
|  | {YYYYYY}3 | 0.00% | ± | 0.00% | | 0.00% | | ± | | 0.00% | | 0.00% | | ± | | 0.00% | | 0.02% | | ± | | 0.03% | | 0.52% | | ± | | 0.48% | | 0.11% | | ± | 0.19% | | |  |
|  | {YYYYYY}4 | 0.00% | ± | 0.00% | | 0.00% | | ± | | 0.00% | | 0.00% | | ± | | 0.00% | | 0.06% | | ± | | 0.06% | | 0.00% | | ± | | 0.00% | | 3.79% | | ± | 1.33% | | |  |
|  | {YYYYYY}5 | 0.05% | ± | 0.02% | | 0.00% | | ± | | 0.00% | | 0.17% | | ± | | 0.05% | | 0.18% | | ± | | 0.10% | | 0.58% | | ± | | 0.93% | | 0.96% | | ± | 0.80% | | |  |
|  | {111111} | 0.01% | ± | 0.02% | | 0.02% | | ± | | 0.02% | | 0.00% | | ± | | 0.00% | | 0.02% | | ± | | 0.03% | | 0.41% | | ± | | 0.63% | | 1.32% | | ± | 1.75% | | |  |
| Phe-336 | {000000000} | 99.88% | ± | 0.04% | | 99.76% | | ± | | 0.06% | | 99.80% | | ± | | 0.15% | | 99.74% | | ± | | 0.07% | | 94.97% | | ± | | 0.85% | | 95.03% | | ± | 1.62% | | |  |
|  | {YYYYYYYYY}1 | 0.00% | ± | 0.00% | | 0.07% | | ± | | 0.12% | | 0.00% | | ± | | 0.00% | | 0.06% | | ± | | 0.10% | | 0.40% | | ± | | 0.52% | | 0.00% | | ± | 0.00% | | |  |
|  | {YYYYYYYYY}2 | 0.05% | ± | 0.04% | | 0.14% | | ± | | 0.15% | | 0.09% | | ± | | 0.13% | | 0.07% | | ± | | 0.09% | | 0.19% | | ± | | 0.33% | | 1.55% | | ± | 1.50% | | |  |
|  | {YYYYYYYYY}3 | 0.00% | ± | 0.01% | | 0.00% | | ± | | 0.00% | | 0.03% | | ± | | 0.05% | | 0.02% | | ± | | 0.02% | | 0.28% | | ± | | 0.49% | | 0.00% | | ± | 0.00% | | |  |
|  | {YYYYYYYYY}4 | 0.01% | ± | 0.01% | | 0.00% | | ± | | 0.00% | | 0.02% | | ± | | 0.04% | | 0.07% | | ± | | 0.06% | | 0.35% | | ± | | 0.60% | | 0.72% | | ± | 0.76% | | |  |
|  | {YYYYYYYYY}5 | 0.00% | ± | 0.00% | | 0.00% | | ± | | 0.00% | | 0.00% | | ± | | 0.00% | | 0.00% | | ± | | 0.00% | | 0.87% | | ± | | 1.08% | | 0.59% | | ± | 0.75% | | |  |
|  | {YYYYYYYYY}6 | 0.02% | ± | 0.01% | | 0.00% | | ± | | 0.01% | | 0.03% | | ± | | 0.03% | | 0.02% | | ± | | 0.04% | | 0.78% | | ± | | 1.35% | | 0.76% | | ± | 0.70% | | |  |
|  | {YYYYYYYYY}7 | 0.01% | ± | 0.02% | | 0.01% | | ± | | 0.01% | | 0.02% | | ± | | 0.04% | | 0.00% | | ± | | 0.00% | | 0.06% | | ± | | 0.10% | | 0.00% | | ± | 0.00% | | |  |
|  | {YYYYYYYYY}8 | 0.00% | ± | 0.00% | | 0.00% | | ± | | 0.00% | | 0.00% | | ± | | 0.01% | | 0.00% | | ± | | 0.00% | | 0.51% | | ± | | 0.78% | | 0.15% | | ± | 0.13% | | |  |
|  | {111111111} | 0.02% | ± | 0.03% | | 0.01% | | ± | | 0.01% | | 0.01% | | ± | | 0.01% | | 0.03% | | ± | | 0.02% | | 1.59% | | ± | | 0.89% | | 1.20% | | ± | 1.50% | | |  |

|  |  | Caco-2 cells infected with *Stm* 14028 | | | | | | | | | | | | | | | | | |
| --- | --- | --- | --- | --- | --- | --- | --- | --- | --- | --- | --- | --- | --- | --- | --- | --- | --- | --- | --- |
|  |  | wildtype | | | Δ*uhpT, ptsG, manXYZ* | | | Δ*ptsG, manXYZ* | | |  Δ*uhpT* | | | wildtype | | | Δ*uhpT, ptsG, manXYZ* | | |
| Tyr-466 | {000000000} | 99.07% | ± | 0.97% | 63.50% | ± | 55.13% | 88.17% | ± | 10.99% | 90.95% | ± | 5.93% |  |  |  |  |  |  |
|  | {YYYYYYYYY}1 | 0.00% | ± | 0.00% | 0.13% | ± | 0.22% | 5.58% | ± | 2.97% | 0.01% | ± | 0.02% |  |  |  |  |  |  |
|  | {YYYYYYYYY}2 | 0.00% | ± | 0.00% | 0.02% | ± | 0.04% | 0.00% | ± | 0.00% | 3.67% | ± | 6.35% |  |  |  |  |  |  |
|  | {YYYYYYYYY}3 | 0.00% | ± | 0.00% | 1.08% | ± | 1.53% | 2.83% | ± | 4.00% | 0.00% | ± | 0.00% |  |  |  |  |  |  |
|  | {YYYYYYYYY}4 | 0.00% | ± | 0.00% | 0.00% | ± | 0.00% | 1.52% | ± | 2.15% | 0.00% | ± | 0.00% |  |  |  |  |  |  |
|  | {YYYYYYYYY}5 | 0.00% | ± | 0.00% | 0.07% | ± | 0.12% | 1.33% | ± | 1.88% | 0.00% | ± | 0.00% |  |  |  |  |  |  |
|  | {YYYYYYYYY}6 | 0.03% | ± | 0.05% | 2.11% | ± | 3.29% | 0.28% | ± | 0.40% | 3.18% | ± | 3.65% |  |  |  |  |  |  |
|  | {YYYYYYYYY}7 | 0.90% | ± | 0.97% | 0.09% | ± | 0.15% | 0.29% | ± | 0.41% | 2.19% | ± | 3.79% |  |  |  |  |  |  |
|  | {YYYYYYYYY}8 | 0.00% | ± | 0.00% | 4.27% | ± | 7.39% | 0.00% | ± | 0.00% | 0.00% | ± | 0.00% |  |  |  |  |  |  |
|  | {111111111} | 0.00% | ± | 0.00% | 28.73% | ± | 49.77% | 0.00% | ± | 0.00% | 0.00% | ± | 0.00% |  |  |  |  |  |  |
| Ile-274 | {X00000} | 98.12% | ± | 0.13% | 98.09% | ± | 0.08% | 98.14% | ± | 0.07% | 98.33% | ± | 0.13% | 99.71% | ± | 0.15% | 99.57% | ± | 0.38% |
|  | {XYYYYY}1 | 1.83% | ± | 0.18% | 1.88% | ± | 0.06% | 1.75% | ± | 0.12% | 1.64% | ± | 0.12% | 0.00% | ± | 0.00% | 0.00% | ± | 0.00% |
|  | {XYYYYY}2 | 0.03% | ± | 0.05% | 0.02% | ± | 0.03% | 0.09% | ± | 0.07% | 0.00% | ± | 0.00% | 0.13% | ± | 0.11% | 0.21% | ± | 0.23% |
|  | {XYYYYY}3 | 0.01% | ± | 0.02% | 0.00% | ± | 0.01% | 0.01% | ± | 0.01% | 0.02% | ± | 0.02% | 0.09% | ± | 0.15% | 0.04% | ± | 0.07% |
|  | {XYYYYY}4 | 0.00% | ± | 0.01% | 0.01% | ± | 0.02% | 0.01% | ± | 0.01% | 0.00% | ± | 0.01% | 0.06% | ± | 0.11% | 0.10% | ± | 0.10% |
|  | {X11111} | 0.00% | ± | 0.00% | 0.00% | ± | 0.00% | 0.00% | ± | 0.00% | 0.01% | ± | 0.01% | 0.00% | ± | 0.00% | 0.07% | ± | 0.09% |
| Leu-200 | {X00000} | 99.79% | ± | 0.06% | 99.81% | ± | 0.05% | 99.79% | ± | 0.07% | 99.86% | ± | 0.04% | 99.80% | ± | 0.04% | 99.85% | ± | 0.10% |
|  | {XYYYYY}1 | 0.20% | ± | 0.06% | 0.18% | ± | 0.05% | 0.21% | ± | 0.07% | 0.13% | ± | 0.04% | 0.00% | ± | 0.00% | 0.00% | ± | 0.00% |
|  | {XYYYYY}2 | 0.00% | ± | 0.00% | 0.00% | ± | 0.00% | 0.00% | ± | 0.00% | 0.00% | ± | 0.00% | 0.09% | ± | 0.04% | 0.05% | ± | 0.06% |
|  | {XYYYYY}3 | 0.00% | ± | 0.00% | 0.00% | ± | 0.00% | 0.00% | ± | 0.00% | 0.00% | ± | 0.00% | 0.03% | ± | 0.06% | 0.04% | ± | 0.04% |
|  | {XYYYYY}4 | 0.00% | ± | 0.00% | 0.00% | ± | 0.00% | 0.00% | ± | 0.00% | 0.00% | ± | 0.00% | 0.03% | ± | 0.04% | 0.00% | ± | 0.00% |
|  | {X11111} | 0.01% | ± | 0.00% | 0.01% | ± | 0.01% | 0.00% | ± | 0.01% | 0.01% | ± | 0.01% | 0.04% | ± | 0.03% | 0.05% | ± | 0.08% |
