# Supplementary material for: Carbon Metabolism of Enterobacterial Human Pathogens Growing in Epithelial Colorectal Adenocarcinoma (Caco-2) Cells
Source: PLoS One. 2010 May 11;5(5):e10586. doi: 10.1371/journal.pone.0010586 (PMC2868055; doi:10.1371/journal.pone.0010586)
Supplement: Table S5 — 13C-Excess (%) per C-atom in amino acids from experiments a-n, for details see Figure 4. (0.04 MB DOC) [file pone.0010586.s007.doc]

**Table S5**: ^13^C-Excess (%) per C-atom in amino acids from experiments a-n, for details see Figure 4

|  | Glucose labelling | | Prelabelled host cells | | | | | | | | | | | |
| --- | --- | --- | --- | --- | --- | --- | --- | --- | --- | --- | --- | --- | --- | --- |
|  | Caco-2 cells | | Bacteria | | | | | | Caco-2 infected with | | | | | |
|  |  |  | *EIEC* HN280 | | *EIEC* 4608-58 | | *Stm* 14028 | | *EIEC* HN280 | | *EIEC* 4608-58 | | *Stm* 14028 | |
|  | a | b | c | d | e | f | g | h | i | j | k | l | m | n |
| Ala | 11.44 % | 11.72 % | 17.06 % | 26.47 % | 57.42 % | 54.38 % | 35.88 % | 40.42 % | 48.64 % | 47.31 % | 50.36 % | 52.07 % | 51.15 % | 51.05 % |
| Asp | 2.62 % | 2.04 % | 4.67 % | 7.09 % | 17.05 % | 15.98 % | 12.45 % | 14.04 % | 15.31 % | 15.18 % | 17.01 % | 18.04 % | 18.06 % | 18.76 % |
| Glu | 4.15 % | 3.08 % | 6.75 % | 10.01 % | 21.63 % | 21.20 % | 15.93 % | 17.65 % | 15.93 % | 15.37 % | 16.55 % | 18.57 % | 18.22 % | 18.52 % |
| Gly | 2.02 % | 2.23 % | 4.69 % | 5.65 % | 9.54 % | 8.97 % | 7.94 % | 9.10 % | 9.55 % | 9.78 % | 8.49 % | 9.43 % | 9.87 % | 10.90 % |
| Pro | 1.63 % | 0.55 % | 4.34 % | 4.69 % | 11.06 % | 7.80 % | 9.16 % | 10.35 % | 8.94 % | 8.73 % | 11.64 % | 12.54 % | 12.80 % | 13.96 % |
| Ser | 2.24 % | 3.26 % | 5.96 % | 8.59 % | 13.81 % | 13.59 % | 11.53 % | 13.03 % | 14.19 % | 13.67 % | 13.13 % | 13.68 % | 14.09 % | 15.02 % |
